# A systematic review of biomarkers for disease progression in Parkinson’s disease

**DOI:** 10.1186/1471-2377-13-35

**Published:** 2013-04-12

**Authors:** David JM McGhee, Pamela L Royle, Paul A Thompson, David E Wright, John P Zajicek, Carl E Counsell

**Affiliations:** 1 Division of Applied Health Sciences, University of Aberdeen, Polwarth Building, Foresterhill, Aberdeen, AB25 2ZD, UK; 2 Division of Health Sciences, University of Warwick, Coventry, CV4 7AL, UK; 3 Centre for Health and Environmental Statistics, Plymouth University, ITTC Building, Tamar Science Park, Plymouth, PL6 8BX, UK; 4 Clinical Neurology Research Group, Peninsula College of Medicine and Dentistry, University of Plymouth, Tamar Science Park, Plymouth, PL6 8BX, UK

**Keywords:** Parkinson disease, Biomarkers, Disease progression, Clinical trials, Neuroprotective agents

## Abstract

**Background:**

Using surrogate biomarkers for disease progression as endpoints in neuroprotective clinical trials may help differentiate symptomatic effects of potential neuroprotective agents from true disease-modifying effects. A systematic review was undertaken to determine what biomarkers for disease progression in Parkinson’s disease (PD) exist.

**Methods:**

MEDLINE and EMBASE (1950–2010) were searched using five search strategies. Abstracts were assessed to identify papers meriting review in full. Studies of participants with idiopathic PD diagnosed by formal criteria or clearly described clinical means were included. We made no restriction on age, disease duration, drug treatment, or study design. We included studies which attempted to draw associations between any tests used to investigate disease progression and any clinical measures of disease progression. The electronic search was validated by hand-searching the two journals from which most included articles came.

**Results:**

183 studies were included: 163 (89%) cross-sectional, 20 (11%) longitudinal. The electronic search strategy had a sensitivity of 71.4% (95% CI 51.1-86.0) and a specificity of 97.1% (95% CI 96.5-97.7). In longitudinal studies median follow-up was 2.0 years (IQR 1.1-3.5). Included studies were generally poor quality - cross-sectional with small numbers of participants, applying excessive inclusion/exclusion criteria, with flawed methodologies and simplistic statistical analyses.

**Conclusion:**

We found insufficient evidence to recommend the use of any biomarker for disease progression in PD clinical trials, which may simply reflect the poor quality of research in this area. We therefore present a provisional ‘roadmap’ for conducting future disease progression biomarker studies, and recommend new quality criteria by which future studies may be judged.

## Background

Parkinson’s disease (PD) is the second most common progressive neurodegenerative disorder, with an annual incidence of around 16–19 cases per 100,000 [1]. As drug therapy in PD is currently symptomatic in nature, a key aim of PD research is the development of drugs which slow or even halt neurodegeneration and, therefore, clinical progression. However, clinical trials in neurodegenerative disorders have struggled to separate out symptomatic effects of potential therapeutic agents (e.g. due to increased striatal dopamine) from true disease-modifying effects. In PD, it is currently not possible to directly measure the number of remaining dopaminergic neurons in vivo and, therefore, alternative approaches are required. Clinical assessments in PD using scales to measure motor impairment, disability, quality of life, or disease stage scales are affected by symptomatic effects of therapy and are unable to differentiate this effect from disease-modification, at least in the short-term.

Various clinical trial designs have been developed to try to adjust for symptomatic effects of putative neuroprotective agents and, therefore, allow clinical rating scales to be used as endpoints. These include long-term follow up studies of placebo-treated and active-agent treated patients looking for ongoing divergence, measuring outcomes following a wash-out period [2,3], and delayed start trial design [4]. However, limitations and flaws in these trial designs have as yet restricted their use. An alternative approach, the focus of much primary research, is the use of a surrogate outcome biomarker as an endpoint in neuroprotective clinical trials.

Surrogate outcome biomarkers are objectively measured characteristics of a disease, which act as indicators of the underlying pathogenic process responsible for disease progression, including the change in that process following a therapeutic intervention [5,6]. To allow their use in clinical trials surrogate outcome biomarkers must have a strong association with a clinical endpoint or outcome known to measure the effect of a therapeutic intervention on disease progression, for which the biomarker can act as a substitute. Surrogate biomarkers for disease progression in PD could shorten the duration of phase III trials and thereby reduce the cost and time required to get a drug to market. Unfortunately at present there is not a single accepted surrogate outcome biomarker for any neurodegenerative disorder. Table 1 outlines the ideal characteristics of a surrogate biomarker for disease progression in PD.

**Table 1:** **Study quality questionnaire based on the assay methods and study design sections of the REMARK reporting recommendations for prognostic tumour markers **[18]

**Question**	**Yes = 1**	**No = 0**
(1) Was the study prospective?	The study reports that patients and the performed test result were collected before the development of an outcome.	No report or clearly retrospective (e.g. patients with poor prognosis collected before biomarker measurement).
(2) Was evaluation of prognostic marker blinded to patient outcome?	The study reports an attempt to blind the person measuring the biomarker to patient outcome.	There is no such report, or assessor clearly not blinded.
(3) Was there a defined time period during which patients were enrolled?	Study defines time period, end of follow-up period, and median follow-up time.	Does not define criteria.
(4) Were there precisely defined clinical outcomes at the start of the study?	Study defines which clinical end points are to be measured.	No such definition.
(5) Were the methods for measuring the prognostic marker adequately described and referenced?	Clearly described and referenced	Not clearly described and referenced
(6) Cases unselected and unbiased?	No attempt to select patients with exclusion criteria	Only a subset of patients enter the study

Much has been written about what features biomarkers for disease progression in Parkinson’s disease should possess [7,8]. The ideal surrogate biomarker should:

(1) Change with neurodegeneration (i.e. degeneration of the nigrostriatal dopaminergic system);

(2) Show an association with the clinical phenotype arising secondary to this degenerative process;

(3) Have a direct association with disease progression, without intermediate variables;

(4) Have robust longitudinal data linking it to disease progression;

(5) Not be influenced by symptomatic treatment, but only by a true change in the neurodegenerative process;

(6) Predict long-term changes in disease progression by short-term changes in the biomarker;

(7) Be generalisable to people with differing characteristics (e.g. age, gender, race);

(8) Be continually variable (ideally linearly for simplicity);

(9) Be sensitive, reflecting small changes in disease progression;

(10) Be quick and cheap to measure, and amenable to blinded assessment;

(11) Be suitable for measurement reliably across different centres;

(12) Be suitable for repeated measurement in the same patient;

(13) Be safe and tolerable to the patient.

PD is a complex neurodegenerative disorder in which many different pathophysiological processes have been identified in the nigrostriatal pathways and beyond, such as protein aggregation, oxidative damage, mitochondrial dysfunction, lysosomal dysfunction and inflammation. It is, therefore, not surprising that many different candidate biomarkers for disease progression in PD have been studied. However, the literature in this area has never been brought together systematically. We, therefore, aimed to undertake a systematic review to assess what potential surrogate biomarkers for disease progression (motor and non-motor) in PD exist, whether any meet the criteria for use in clinical trials, and if not which looks most promising. We did not aim to review the literature for diagnostic biomarkers (i.e. those designed to aid early diagnosis in the pre-motor or motor phase) or prognostic biomarkers (i.e. those aimed at identifying patients who progress at different rates). In addition, we aimed to critique data from identified disease progression biomarker studies relating to study design, participant characteristics, and statistical analyses undertaken, in order to produce guidelines for future studies.

## Methods

Following the development of a review protocol equivalent to the methodology described below, literature searches were conducted in the databases MEDLINE (1950 to August 2010) and Embase (1980 to August 2010), using the OVID search interface. Five separate search strategies, developed by an experienced information scientist, were run in each database. The first four were based on free-text words identified through background reading of relevant review articles. These searches included potential ([1]) blood, ([2]) urine or cerebrospinal fluid (CSF), ([3]) imaging and ([4]) neurophysiological biomarkers. A fifth search using generic terms for biomarkers based on index headings was also run in both databases. For details of the search strategy please see Additional file 1.

The searches were limited to human studies. Only English language articles were included, due to lack of resources for translation. Reference lists of included articles and relevant review articles were checked to identify any studies which the electronic search may have missed.

### Validation of the electronic search strategy

The electronic search strategy was validated by hand searching five years of the two journals from which most of the included articles came: Movement Disorders (2002–2006) and Journal of the Neurological Sciences (1992–1996). The number of relevant and irrelevant articles identified by hand searching and by the electronic search, was used to calculate the sensitivity and specificity for the electronic search strategy.

### Study selection

A single reviewer examined abstracts retrieved by the electronic search to identify articles meriting review in full. Full length articles were then reviewed before data were extracted from relevant papers. In both stages the inclusion and exclusion criteria detailed below were applied.

Only studies of participants with idiopathic Parkinson’s disease diagnosed by formal criteria [9-12], or clearly described clinical means (the presence of at least two out of four of the cardinal clinical signs of PD and an attempt to exclude atypical syndromes), were included. No restriction was made on the grounds of participant’s age, disease duration, or drug treatment.

Studies which investigated the efficacy of using a biomarker, including (but not restricted to) imaging, blood tests, tests of CSF and neurophysiological tests, to investigate disease progression in Parkinson’s disease were included. To qualify for inclusion there must have been an attempt to examine for an association between the biomarker and a clinical measure of disease progression. Acceptable measures included measures of motor or cognitive impairment, disability, handicap, quality of life, and duration of survival.

Only studies exploring associations between a biomarker and the total score from a clinical rating scale, rather than its subsections, were included. The subsections of most clinical measures would never be acceptable outcome measures for neuroprotective trials and, therefore, developing surrogate biomarkers for these was felt not to be relevant. The exception was the Unified Parkinson’s disease rating scale (UPDRS) [13], where studies examining for relationships to its main constituent parts were included. Studies only investigated the relationship between a biomarker and individual symptoms (e.g. bradykinesia or rigidity), or olfactory function were excluded for similar reasons.

Only studies examining for associations between putative biomarkers and global measures of cognition (e.g. Mini-mental state examination (MMSE) [14], Cambridge cognitive examination (CAMCOG [15])), rather than individual neuropsychological tests, were included. It is unlikely that improvement in a single neuropsychological test would be a suitable outcome measure for a neuroprotective trial. As depression in PD is not linked to overall disease progression [16] and may be commoner at the time of diagnosis [17], studies only investigating the relationship between a biomarker and depression were excluded.

Studies examining the relationship between a biomarker and treatment status, the presence or severity of complications related to therapy, or duration of illness were excluded. Studies of static predictive biomarkers (e.g. genetic markers) which try to anticipate the future rate of disease progression were excluded. A useful biomarker for clinical trials needs to be dynamic - changing with disease progression. Therefore, these studies were not relevant.

As we aimed to produce a comprehensive review and detect any evidence of the utility of a putative biomarker, we set no study quality threshold. We, therefore, included small cross-sectional studies, in which an association between a biomarker and clinical measures of disease progression were analysed at a single time point across groups of patients with different disease severities.

### Data extraction

Study methods and results were extracted by a single reviewer, and for accuracy this was performed twice. Data were extracted using a data extraction sheet (Additional file 2) relating to the following: (1) study design including restrictiveness of criteria for entry into the study; (2) setting; (3) study population, including number of participants, gender ratio, disease duration at baseline, baseline measures of disease severity and baseline treatment status; (4) specific biomarkers investigated; (5) statistical analyses performed; (6) results of statistical analyses of the associations between the biomarkers and clinical measures of disease severity and how completely these results were reported; (7) analysis of the effect of drug treatment on the biomarker; (8) economic analysis of using the biomarker; (9) measures of suitability and acceptability of the test to patients.

The restrictiveness of the inclusion and exclusion criteria applied in each study was graded as: none, explicit statement that only criteria to exclude atypical parkinsonian syndromes were applied; mild, ≤ 3 criteria applied (except those described under moderate); moderate, 4–5 criteria applied or evidence of an attempt to limit by age, gender, cognitive state, disease stage, drug therapy for PD (e.g. all de-novo); severe, ≥ 6 criteria applied; not detailed, no mention of whether criteria were applied.

### Methodological quality

No validated tool to measure the quality of studies investigating surrogate biomarkers as outcome measures exists. An attempt was, therefore, made to assess study quality using an adapted quality questionnaire, illustrated in Table 1, based on the assay methods and study design sections of the Reporting recommendations for tumor MARKer prognostic studies (REMARK) reporting recommendations for prognostic tumour markers [18]. This measure of study quality was also used to assess whether there was any bias in terms of the quality of studies included.

### Data synthesis

Given the likelihood that included studies would examine the relationship of multiple different putative biomarkers with multiple different clinical measures of disease severity, we were aware that any data synthesis would be qualitative in nature.

## Results

**Figure 1:**
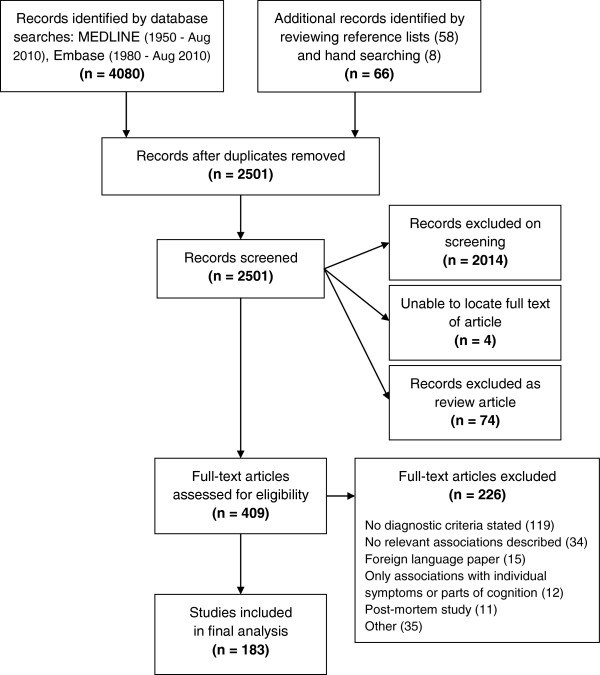
**Flow diagram outlying the selection procedure to identify 183 articles included in the systematic review of biomarkers for disease progression in PD.** Note that of the 58 articles identified by reviewing reference lists, 41 were excluded, one article could not be located and 16 were included in the final qualitative synthesis. All eight articles identified by hand searching (all cross-sectional) were included in the final qualitative synthesis.

As shown in Figure 1, the electronic search identified 4080 records. After removing duplicates, 2435 unique records identified by the electronic search were screened, in addition to a further 66 records identified whilst performing the hand search or on reviewing reference lists of relevant review articles and included articles. The full-text articles of 409 records were then assessed for eligibility, and of those 226 articles were excluded. Finally data were extracted from a total of 183 articles.

### Hand searching

Hand searching to validate the electronic search strategy revealed a sensitivity of 71.4% (95% CI 51.1-86.0) and a specificity of 97.1% (95% CI 96.5-97.7).

### Characteristics of included articles

The majority of included articles (n=156, 85%) had a cross-sectional study design. The remaining 27 articles had a longitudinal design, but only 20 (11%) of these attempted to examine for relevant longitudinal relationships between a change in a biomarker and a change in a clinical measure of disease severity. The other seven (4%) simply detailed cross-sectional analyses, and results from these have been included with data from cross-sectional studies.

The UK Parkinson’s Disease Society (UKPDS) Brain Bank Criteria [9] was most commonly used to make the diagnosis of PD (Table 2), although a large proportion of studies simply used the presence of the cardinal clinical features of PD. Over half of included articles did not describe the setting of their study, but the majority of those who did were based in outpatient departments. Similarly, a third of studies failed to mention whether inclusion and exclusion criteria were applied. Of those providing this information over half applied moderately to severely restrictive inclusion/exclusion criteria. No significant differences in study characteristics were noted between longitudinal and cross-sectional studies.

**Table 2:** Study characteristics of the 183 included articles

	**All studies (n=183)**		**Longitudinal studies (n=20)**		**Cross-sectional studies (n=163)**		**P value**
	**n**	**%**	**n**	**%**	**n**	**%**	
**Diagnostic criteria**							
UKPDS Brain Bank criteria	89	48.6	11	55.0	78	47.9	0.472
Clinical features	53	29.0	8	40.0	45	27.6	
Calne criteria	16	8.7	1	5.0	15	9.2	
National Institute of Neurological Disorders and Stroke criteria	11	6.0	0	0.0	11	6.7	
Weiner and Lang criteria	9	4.9	0	0.0	9	5.5	
Other	5	2.7	0	0.0	5	3.1	
**Setting**							
Outpatient	86	47.0	10	50.0	76	46.6	0.859
Inpatient	2	1.1	0	0.0	2	1.2	
Not detailed	95	51.9	10	50.0	85	52.2	
**Inclusion/Exclusion criteria**							
None	1	0.5	0	0.0	1	0.6	0.213
Mildly restrictive	22	12.0	1	5.0	21	12.9	
Moderately restrictive	65	35.5	11	55.0	54	33.1	
Severely restrictive	35	19.1	1	5.0	34	20.9	
Not detailed	60	32.8	7	35.0	53	32.5	

The P value column relates to association between study type (longitudinal and cross-sectional studies) and 1) Diagnostic criteria, 2) Setting, 3) Inclusion/exclusion criteria using Pearson’s chi squared tests.

All of the included studies used an impairment or disability scale as the clinical measure of disease progression used to test for an association with a biomarker. None of the studies used duration of survival, or measures of quality of life or handicap as a clinical outcome measure.

### Characteristics of study participants

As illustrated in Table 3 the median number of study participants was low at 32 (interquartile range (IQR) 21 to 53). With regards to representativeness of all those with PD, those included were fairly young, with a mean age of 63.5 (standard deviation (SD) 5.6) years of age, particularly considering that the median duration of disease at study entry was 5.7 (IQR 3.6 to 7.4) years. In keeping with this fairly long duration of disease most included patients were already on treatment at baseline, and had moderate disease severity scores. Cognition was only mildly impaired, with many studies excluding patients with dementia.

**Table 3:** Baseline characteristics of study participants in the 183 included articles

	**All studies (n=183)**		**Longitudinal studies (n=20)**		**Cross-sectional studies (n=163)**		**P value**
**Baseline demographics**							
Median number of patients	32	(21 to 53)	31	(16 to 36)	34	(22 to 55)	0.058
Mean age (years)	63.5	(5.6)	63.7	(6.7)	63.4	(5.5)	0.771
Mean percentage male	58.7	(13.9)	67.3	(15.6)	57.7	(13.3)	**0.002**
Median disease duration (years)	5.7	(3.6 to 7.4)	3.2	(1.7 to 5.9)	5.9	(4.1 to 7.6)	**0.005**
Median percentage treated	76.8	(51.2 to 88.8)	40.6	(0.0 to 71.1)	78.5	(56.7 to 90.2)	**0.005**
**Baseline disease severity**							
Median total UPDRS	33.3	(29.4 to 40.6)	31.8	(30.1 to 38.7)	33.3	(29.4 to 40.6)	0.790
Median UPDRS (II)	14.7	(11.6 to 16.3)	9.1	(Insufficient data for IQR)	14.8	(11.9 to 16.6)	**0.023**
Median UPDRS (III)	21.0	(16.6 to 26.3)	22.4	(16.3 to 28.5)	20.9	(16.8 to 25.8)	0.864
Median Hoehn & Yahr	2.5	(2.0 to 2.8)	1.8	(1.6 to 2.0)	2.5	(2.1 to 2.9)	**<0.001**
Median MMSE	25.8	(23.0 to 28.1)	25.5	(22.3 to 28.3)	25.8	(23.0 to 28.1)	1.000

Means are presented with standard deviations, and medians with interquartile ranges (IQR). The P value column relates to comparisons made between longitudinal and cross-sectional studies using the Mann–Whitney test.

Patients included in longitudinal studies had a significantly shorter disease duration at baseline (P = 0.005) and were less likely to be on treatment (P = 0.005) than those included in cross-sectional studies. In keeping with this, they also had milder disease at baseline, with significantly better UPDRS (II) scores and lower Hoehn and Yahr stages than those in cross-sectional studies.

### Quality criteria

The median total score produced by applying the adapted quality questionnaire to each of the included studies was 4.0 (IQR 3.0 to 4.0) out of a possible six. There was no significant difference in the total score achieved by longitudinal (median 4.0 (IQR 3.0 to 4.8)) and cross-sectional (median 4.0 (IQR 3.0 to 4.0) studies (P = 0.125, Mann Whitney test). Unfortunately three of the six questions (questions one, four and five) had almost no discriminating value, being rated in the affirmative for almost every included article.

### Types of biomarkers investigated

A broad spectrum of different biomarker modalities were investigated in the studies included in this systematic review (Table 4). Brain single-photon emission tomography (SPECT) and positron emission tomography (PET) imaging, along with tests of blood and its constituents, and cerebrospinal fluid formed the majority of biomarkers investigated. Longitudinal studies mainly examined the potential of PET and SPECT imaging as a biomarker of disease progression.

**Table 4:** Types of putative biomarkers for disease progression investigated in the 183 included articles

	**All studies (n=183)**		**Longitudinal studies (n=20)**		**Cross-sectional studies (n=163)**	
**Biomarker modality**	**Number of studies examining biomarker**	**%**	**Number of studies examining biomarker**	**%**	**Number of studies examining biomarker**	**%**
Serum/plasma/blood	51	27.9	0	0.0	51	31.3
Brain SPECT	41	22.4	9	45.0	32	19.6
Brain PET	31	16.9	8	40.0	23	14.1
CSF	29	15.8	0	0.0	29	17.8
Brain MRI	15	8.2	1	5.0	14	8.6
Cardiac ^123^I-MIBG scintigraphy	9	4.9	0	0.0	9	5.5
Electrophysiology	9	4.9	1	5.0	8	4.9
Ultrasound	7	3.8	0	0.0	7	4.3
Urine	5	2.7	0	0.0	5	3.1
Brain MRS	2	1.1	0	0.0	2	1.2
Other	3	1.6	1	5.0	2	1.2

19 cross-sectional studies examined for a relationship between two different biomarker modalities and a clinical measure of disease progression. (SPECT, single-photon emission computed tomography; PET, positron emission tomography; CSF, cerebrospinal fluid; MRI, magnetic resonance imaging; ^123^I-MIBG, ^123^I-metaiodobenzylguanidine; MRS, magnetic resonance spectroscopy).

### Longitudinal studies

In the 20 studies which examined for relevant longitudinal associations, the mean number of longitudinal time points (including the baseline time point) was 2.2 (SD 0.4), with a median time period from baseline to the final time point of 2.0 (IQR 1.1 to 3.5) years.

Additional file 3 details the biomarkers examined in the 20 longitudinal studies and their relationship with clinical measures of disease severity. Nine undertook brain SPECT imaging, eight brain PET imaging, one brain MRI, one electrophysiological studies, and one acoustical analysis following a four sentence reading task. Among other parameters the imaging studies examined the relationship between clinical measures (total UPDRS, UPDRS (II), UPDRS (III), Hoehn and Yahr stage [19], MMSE and CAMCOG) and uptake in the caudate, putamen and striatum. A variety of different PET and SPECT ligands were used. These included ligands which provide a measure of cerebral glucose metabolism ([^18^F]-2-fluoro-2-deoxyglucose [FDG]) and cerebral blood flow (N-isopropyl-P[^123^I]-iodoamphetamine; [^99m^Tc]-hexamethylpropylene amine oxidase [HMPAO]), although the majority were ligands used to measure pre-synaptic dopaminergic function, either relating to fluorodopamine synthesis and storage ([^18^F]6-fluoro-L-3,4-dihydroxyphenylalanine) [FDOPA]) or dopamine active transporter (DAT) function ([^18^F]-2β-carbomethoxy-3β-(4-iodophenyl)-N-(3-fluoropropyl)-N-tropane [[^18^F]FP-CIT]; [^123^I]-2β-carbomethoxy-3β-(4-iodophenyl)-N-(3-fluoropropyl)-N-tropane [[^123^I]FP-CIT]; [^123^I]-2β-carbomethoxy-3β-(4-iodophenyl tropane) [[^123^I]β-CIT]; 2β-carbomethoxy-3β-(4-[^18^F])-fluorophenyl) tropane [[^18^F]CFT]). This marked heterogeneity in the studies unfortunately made quantitative synthesis of the extracted data impossible.

It is clear from the data presented in Additional file 3 that there is no evidence to support the use of acoustic analysis or electrophysiological tests as biomarkers for disease progression in PD. The majority of the imaging studies found no relationship between imaging parameters and clinical measures of disease severity, as illustrated in Table 5. Those which did report a significant relationship were single studies, examining specific brain regions, with results which have not been replicated by another group. In addition they involved small numbers of participants.

**Table 5:** Comparison of the number of included longitudinal studies investigating a given biomarker modality with the number reporting a significant association between the biomarker modality and a clinical measure of disease progression

**Biomarker modality**	**Number of studies investigating biomarker modality**	**Number of studies reporting a significant association between biomarker modality and a clinical measure of disease progression**
FDOPA brain PET	5	0
DAT brain PET or SPECT	8	3
FDG brain PET	2	2
SPECT to investigate cerebral blood flow	3	2
MRI brain	1	0
Electrophysiological tests	1	0
Other	1	0

PET and SPECT brain imaging studies which used ligands to examine dopamine active transporter function are grouped together. SPECT studies which use ligands to investigate cerebral blood flow are also grouped together. (SPECT, single-photon emission computed tomography; PET, positron emission tomography; MRI, magnetic resonance imaging; FDOPA, [^18^F]6-fluoro-L-3,4-dihydroxyphenylalanine; FDG, [^18^F]-2-fluoro-2-deoxyglucose; DAT, dopamine active transporter).

No studies reported an economic analysis of using the biomarker in question, and nor did any report on the acceptability of the test to individual patients. Whilst eight (40%) longitudinal studies did not detail the treatment status of patients at baseline, three studies [20-22] did look at the effect of drug therapy on the biomarker under examination.

In several longitudinal imaging studies it was impossible to tell from the text whether the values derived from specific brain regions which were used to draw associations with clinical measures, were mean values (values from left and right hemispheric structures summed and divided by two) or total values (values from left and right hemispheric structures simply added together). This issue was however far more prolific when examining data from cross-sectional studies.

### Cross-sectional studies

Additional file 4 demonstrates the vast array of putative biomarkers which have been examined in cross-sectional studies, and the large number of clinical measures used to try to draw associations with them. Putative blood and CSF biomarkers examined include structural proteins, neurotransmitters, free-radicals and markers of oxidative stress, amino acids and their metabolites, catecholamines, metals and their transfer proteins, vitamins and their carrier proteins, and cytokines. This heterogeneity made quantitative synthesis of the extracted data impossible. Looking at the data however, no single biomarker investigated stands out amongst the others as a good candidate for further investigation in a longitudinal study.

Similar to the longitudinal studies, in many cross-sectional SPECT and PET studies it was impossible to determine whether the uptake values used to draw associations with clinical measures, were average or total values for structures present in both hemispheres.

Unfortunately none of the 163 studies treated as cross-sectional in this review included an analysis of the acceptability of the test in question to the patients. Regarding the influence of anti-parkinsonian drug therapy on the biomarker examined, 25 cross-sectional studies did examine this issue to some extent.

### Statistics

Correlation analysis, a basic statistical method which can be used to examine for a relationship between two variables, was solely used in 75% (15/20) of longitudinal studies. Only one study [23] used a relevant regression analysis. This study was not designed as a biomarker study, but rather as a drug study to compare the rate of dopamine neuron degeneration after initial treatment with either pramipexole or levodopa in early PD using [^123^I]β-CIT SPECT. The association between change from baseline in UPDRS score (dependent variable) and the percentage change from baseline in [^123^I]β-CIT uptake (independent variable) was examined using a multiple regression model that adjusted for initial treatment (pramipexole, levodopa) and baseline UPDRS score. The paper states that the analysis was performed separately for each time point (22, 34 and 46 months) at which patients had a follow-up scan. However, despite stating that this occurred, details of the outcome are not given in the paper; correlations are simply described with a correlation coefficient (r value) and the level of significance (P value). There was no analysis that used data from all time points in a single statistical analysis, rather than analysing the change from baseline to each time point separately.

Similarly the majority of cross-sectional studies simply used basic correlation analysis rather than more complex statistical techniques.

Overall the standard of reporting results from statistical analyses in the included longitudinal and cross-sectional studies was poor. 83.1% of longitudinal correlations (examined by Pearson’s or Spearman’s correlation analyses), and 58.8% of cross-sectional correlations, described neither a correlation coefficient nor the direction of association. Furthermore in 81.4% of longitudinal correlations and 61.0% of cross-sectional correlations a P value was not given. No study included in this review detailed confidence intervals for a given correlation coefficient.

## Discussion

We found insufficient evidence to support the use of any biomarker to measure motor or non-motor disease progression in PD clinical trials. It has also proven difficult to determine which of the current candidate biomarkers merits selection over the others for further investigation. Whilst some of the putative imaging biomarkers examined longitudinally (e.g. FDG PET) may look promising given the evidence presented in Table 5, unfortunately this is not really the case. In the case of FDG PET two papers examined this biomarker and both found a significant association between the change in a FDG PET measure and a change in the motor UPDRS. However, both studies only included 15 patients, which is a relatively small number of participants from which to draw conclusions. They also both examined different FDG expression patterns and only found that some (four out of six) of these patterns showed a significant association with a change in the motor UPDRS. Given the differences between the studies it was not possible to meta-analyse their results in a meaningful way. As with other imaging modalities we, therefore, felt there was insufficient evidence to single out FDG PET for further investigation.

**Figure 2:**
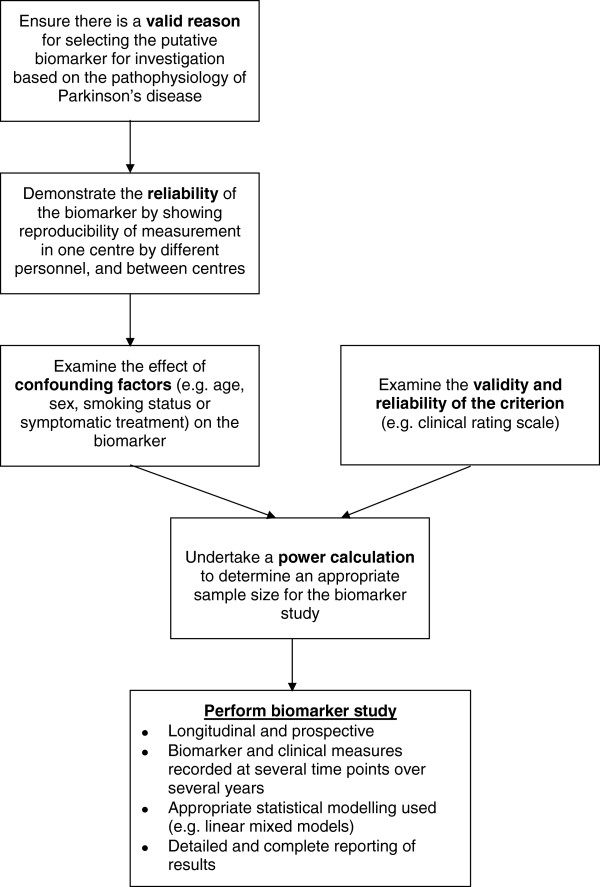
Flow diagram outlying a provisional ‘roadmap’ for conducting a study to determine whether a given biomarker is a suitable surrogate for a clinical measure of disease progression.

It is possible that the lack of a current biomarker is because no suitable biomarker exists or, at least, no single biomarker given the complexity of the disease. However, at present, it probably also reflects the very poor quality of studies which have investigated biomarkers for disease progression in PD. In order to improve future studies, we therefore suggest a provisional ‘roadmap’ for conducting biomarker studies in this area, detailed in Figure 2. In addition, we recommend new quality criteria supported by evidence from this review, detailed in Table 6, by which future studies may be judged.

**Table 6:** New quality criteria to assess studies examining surrogate biomarkers for disease progression

	**Question**	**Yes**	**No**
(1)	Was the primary aim of the study to validate a biomarker for disease progression?		
(2)	Did the study detail a scientifically valid reason for choosing the given biomarker for investigation?		
(3)	Has the reproducibility of measuring the biomarker in the same centre by different trained personnel, and between centres, been evaluated?		
(4)	Has an assessment of the effect of likely confounding factors (e.g. age, gender, smoking status, and being on symptomatic PD treatment) on the measurement of the biomarker been made?		
(5)	Has an assessment of the validity and reliability of the criterion (e.g. clinical rating scale) used been made?		
(6a)	Was a power calculation undertaken to determine the required number of participants?		
(6b)	If a power calculation was undertaken, was the number of participants included appropriate?		
(7)	Was the study longitudinal?		
(8)	Was the study prospective?		
(9)	Was there a sufficient period of follow-up?		
(10)	Were the biomarker and clinical measures of disease severity measured on ≥3 occasions?		
(11)	Was measurement of the biomarker blind to participant characteristics?		
(12)	Did ≥ 75% of participants entering the study complete the full follow-up period?		
(13)	Were cases unselected/unbiased (no exclusion criteria)?		
(14)	Were associations between the biomarker and clinical measures of disease severity examined for using appropriate statistical modelling (e.g. linear mixed modelling) with adjustment for confounding factors, rather than simply correlation analysis?		
(15)	Were results of statistical analyses reported in sufficient detail to allow the inclusion of the study results in a meta-analysis?		

The starting point for any biomarker study must be a valid reason for selecting the specific biomarker for investigation, based on the pathophysiology of PD. In many studies included in this systematic review the reasons for selecting putative biomarkers for investigation were rather tenuous. Just because a substance can be measured does not mean an attempt should be made to find an association between that substance and clinical measures of disease severity. This approach, as demonstrated by this systematic review, is likely to be fruitless. Moreover, the identification of a biomarker was not the primary aim of many studies included in this review but rather a by-product of another study. Disease progression biomarker studies need carefully planned specific designs as highlighted by our ‘roadmap’.

Secondly, the reliability of a putative biomarker must be established by demonstrating the reproducibility of its measurement in a single centre by different personnel, and between different centres. This can be a particular issue for CSF proteins and for imaging biomarkers, where a change in a small area of the brain is often measured. Before proceeding it must be confirmed that such specific imaging biomarkers can be measured with a reliable degree of consistency across different centres, which may have different imaging equipment and software, otherwise the putative biomarker is likely to be of limited use.

Thirdly, an evaluation of the effect of confounding factors on the biomarker (e.g. age, gender, smoking status or being on symptomatic treatment for PD) should be undertaken. An understanding of the influence of confounding factors on the biomarker will aid sample size calculations, and allow a rigorous analysis of the final study results by adjusting for these factors. This hopefully should avoid erroneous results which reflect a confounding factor rather than a true relationship between a biomarker and a clinical measure of disease progression.

Ideally to be a useful biomarker for neuroprotective clinical trials the biomarker should not be influenced by symptomatic treatment. This would simplify trial design meaning patients on and off treatment could be recruited and would mean those going on to treatment would not have to be censored. However, where symptomatic treatment has a limited effect on a given biomarker then knowledge of this may still allow inclusion of patients on treatment in clinical trials with appropriate adjustment of their results. Nonetheless the measurement of biomarkers which will be significantly influenced by symptomatic PD therapy (e.g. plasma dopamine or FDOPA PET uptake) is unlikely to be helpful, as the biomarker will have very limited clinical utility.

In parallel to this pre-study ‘work-up’ of the biomarker, the validity, reliability, and responsiveness, including to clinical change, of the selected criterion (e.g. clinical rating scale) against which a biomarker will be examined, must be explored. Much work has been undertaken in assessing the validity and reliability of psychometric instruments [24], and we would suggest a similar approach here. Maximising the scientific rigor of the selected criterion is central to improving the chance of coming to the correct conclusion about the efficacy of a biomarker for disease progression, and will have implications for biomarker study sample size calculations.

Following completion of all these preliminary steps it should then be possible to undertake a power calculation to determine an appropriate sample size for the biomarker study. Unfortunately no studies included in this review performed a power calculation, and the small number of participants (median 32 (IQR 21 to 53)) in these studies is of concern. As studies get smaller it becomes increasingly likely that potentially significant associations will not be detected, and limits the number of variables that can be included in multivariate analyses without significantly increasing the risk of spurious findings. In a heterogeneous disease such as PD, small sample sizes also mean the cohort is unlikely to be representative of all patients with PD.

Whilst cross-sectional studies formed the majority of articles included in this systematic review, a cross-sectional design is not suitable to examine for a relationship between a change in a clinical measure and the change in a biomarker over time within individual patients with PD. The longitudinal studies included in this review had a median follow-up duration of 2.0 (IQR 1.1 to 3.5) years. There is currently no evidence to suggest what the minimum duration of a surrogate outcome biomarker study should be. Undoubtedly it needs to be long enough for a clinically significant change in the criterion, used to draw associations with the putative biomarker, to be observed. However, if a short-term change in a biomarker is to be associated with a long-term change in a clinical outcome measure then clearly a longer period of follow-up is required. In the included longitudinal studies the biomarker and clinical measures were generally only measured twice (mean 2.2 (SD 0.4) time points), which is clearly insufficient to allow the differentiation of a linear from a non-linear association. Future biomarker studies must be longitudinal, and measure the biomarker and clinical measures at several time points (at least three) over a sufficient follow-up period, which is more likely to be measured in years than months, as only this design will provide sufficient evidence of a biomarkers potential validity.

Parkinson’s disease is a clinically heterogeneous disorder, with patients varying significantly in terms of age of onset, presentation and progression of motor and non-motor features. Ideally, biomarker studies should be large enough and use broad enough inclusion criteria to capture this heterogeneity and identify the utility of the biomarker in different subgroups of patients. The use of moderately to severely restrictive inclusion and exclusion criteria in most of the studies included in this review has influenced the characteristics of the study participants. The mean age of participants in included studies was only 63.5 (SD 5.6) years. Given that the median duration of disease at study entry was 5.7 (IQR 3.6 to 7.4) years, these patients have fairly early onset disease. As PD is mainly a disorder of the elderly [25] these cohorts of patients may, therefore, not be representative of the majority of patients with PD. Future studies should justify their inclusion and exclusion criteria, and try to minimise the latter to avoid limiting the generalisability of their results or highlight their limited generalisability.

The general reporting of results in the included studies was poor, making data extraction and interpretation difficult. This was particularly true of many imaging studies, where there was often ambiguity as to whether stated values were mean values or total values for structures represented in both hemispheres. Similarly there was often a failure to report important methodological features, such as the study setting, or whether inclusion and exclusion criteria were applied.

The reporting of the statistical analyses was also inadequate. In both correlation and regression analyses, hypothesis testing can be undertaken to determine whether a relationship exists in the population as a whole, and confidence intervals calculated to indicate the strength of that relationship. Whilst significance testing was undertaken in most included studies, they failed to report confidence intervals, thereby limiting the ease of interpretation of analyses. Many studies also omitted to report precise significance values, instead simply giving results descriptive in the text. If due to pressure of space the actual results of statistical analyses cannot be fully included in a journal article then data should be provided as an additional online resource. Disappointingly several studies even failed to detail what statistical techniques they used. Without clear reporting of the study methodology, results, and the outcome of statistical analyses, investigators devalue their study and risk it being excluded from future systemic reviews or meta-analyses.

The statistical techniques applied in the included studies were in many cases inappropriate, and more often than not too simplistic. Unfortunately it appeared that many of the studies included in this review had been conducted without input from an experienced statistician. Whilst a detailed review of the appropriate statistical techniques to use in disease progression biomarker studies is beyond the scope of this review, we wish to make a few key points.

The majority of included cross-sectional studies used correlation analysis to examine for a relationship between clinical outcome measures and a given biomarker. However, correlation is a limited technique to use for this purpose as it only indicates the strength and direction of a relationship between two variables [26]. Furthermore correlation does not allow more complex assessment of the influence of possible confounders, and does not allow the value of one variable to be predicted when the other is known.

Several included studies measured a large number of variables and then calculated multiple individual correlation coefficients and significance values. This sequential testing of multiple variables makes it more likely that a significant correlation will be found simply by chance. Multivariate analysis is the appropriate technique to use when the relationship between a single dependent variable and several explanatory variables is examined at the same time, to avoid the pitfalls of multiple testing. It also has the advantage of allowing the inclusion of factors which may affect the relationship between the variables of interest, and adjusts for these potential confounders. Therefore, whilst correlation analysis can be viewed as a basic statistical technique which can in certain circumstances be used to generate hypotheses, higher levels of statistical analysis should be used to test hypotheses [27].

Use of correlation analysis in longitudinal studies, where repeated measurements are performed, is inappropriate as this takes no account of the possible covariance between measurements recorded in the same individual. However the use of regression is limited to situations where variables are measured twice in the same individual. In these cases regression can be used either by including baseline measurements as a covariant in the model, or by converting the pairs of measurements recorded in each individual into the actual change or percentage change from baseline, before using these calculated values in the regression model.

Where variables are measured three or more times in individuals in longitudinal studies, it is possible to undertake separate analyses to compare variables at baseline and each subsequent time point individually. However, this again introduces repeated testing, increasing the likelihood of finding a significant association by chance. It also takes no account of missing data, common in longitudinal studies due to deaths, loss to follow-up or withdrawal from the study. It is therefore not an approach we would recommend.

A potential solution to these problems is the use higher levels of statistical modelling, for example linear mixed models which can be used to test for linear relationships [28]. In the analysis of repeated measurements from longitudinal studies, these types of models take into account patients who drop-out early when estimates for later time points are made. However, the models also ensure that those with incomplete data do not influence the results as greatly as those with complete data. Using these models, a time effect on the repeated measure, independent from disease progression, is allowed for. In addition as disease progression may differ across time points these types of model can allow for this by introducing a ‘disease progression-by-time interaction’. The non-independence of the repeated measures can be accounted for by simply assuming there is a constant correlation for all pairs of measurements in the same individual or by, for example, varying this correlation as measurements become more widely separated by time.

Therefore, we would strongly recommend that future biomarker studies incorporate a range of analyses, rather than simply correlation, in order to explore the validity of more advanced statistical methods. The use of appropriate statistical techniques should reduce the chance of type I and type II errors, and thereby allow sensible conclusions to be drawn about the efficacy of biomarkers for disease progression. Such analyses should be conducted by experienced statisticians, who therefore, need to be involved in the planning and conduct of future studies.

We attempted to group blood and CSF biomarkers into categories with shared characteristics (e.g. structural proteins, free radicals and markers of oxidative damage, antioxidants, pro-inflammatory cytokines, metals, amino acids, and vitamins) to see if this would allow any sensible sub-grouping of the results. However, the biomarkers did not easily fit into a simple categorical structure and hence we abandoned this approach.

It could be argued that the failure of included longitudinal studies to detect associations between the change in clinical measures of disease progression and the change in a given biomarker reflects a lack of sensitivity to change in the clinical markers of disease progression used, rather than a failure of the biomarker to change with disease progression. However, in the majority of included longitudinal studies there was a clear change in the clinical measures used between time points, making this hypothesis unlikely.

One potential criticism of our systematic review is that we included studies whose primary aim was not to develop a biomarker for disease progression in PD. We did so to ensure that our review was as inclusive as possible and did not miss potential biomarkers. It does, however, raise the question of the appropriateness of studies with an alternative primary aim (for example, drug development) of undertaking these additional, usually cross-sectional, analyses to produce information regarding such associations. As we have highlighted the development of biomarkers for disease progression in PD requires studies with a primary objective of biomarker validation, exemplified by a careful study design. Such studies could be run alongside other studies (e.g. long-term prognostic studies or clinical trials) but only if appropriately planned and funded. Conflicts of interest and issues around the subsequent ownership and availability of biomarkers in the public domain may arise if biomarkers are developed in clinical trials funded by pharmaceutical companies and these issues need to be considered beforehand.

Some of the lessons of this systematic review, in particular the necessity for longitudinal studies measuring putative biomarkers and clinical measures several times over several years, have begun to be realised by some researchers and have started to be put into practice. In PD the Parkinson’s Progression Markers Initiative (PPMI) [29] and the Parkinson’s Disease Biomarkers Program (PDBP) [30] both aim to measure various putative CSF, blood, and imaging biomarkers over several years. Their work, like that of the longitudinal Alzheimer’s Disease Neuroimaging Initiative (ADNI) [31] in Alzheimer’s disease, should mark a major shift in the quality of studies of biomarkers for disease progression, and hopefully lead to advances in this important field.

## Conclusions

This extensive systematic review has highlighted the methodological, statistical and reporting flaws of studies examining biomarkers for disease progression in Parkinson’s disease. It is clear that continuing to publish small cross-sectional studies in this area is unlikely to yield significant results. We have suggested methodological guidelines which we hope will provide a better chance of making progress in this area, and would value feedback on them.

## Abbreviations

[18F]CFT: 2β-carbomethoxy-3β-(4-[^18^F])-fluorophenyl) tropane; [18F]FP-CIT: [^18^F]-2β-carbomethoxy-3β-(4-iodophenyl)-N-(3-fluoropropyl)-N-tropane; [123I]β-CIT: [^123^I]-2β-carbomethoxy-3β-(4-iodophenyl tropane; [123I]FP-CIT: [I]-2β-carbomethoxy-3β-(4-iodophenyl)-N-(3-fluoropropyl)-N-tropane; 123I-MIBG: ^123^I-metaiodobenzylguanidine; CAMCOG: Cambridge cognitive examination; CI: Confidence interval; CSF: Cerebrospinal fluid; DAT: Dopamine active transporter; FDG: [^18^F]-2-fluoro-2-deoxyglucose; FDOPA: [^18^F]6-fluoro-L-3,4-dihydroxyphenylalanine; HMPAO: [^99m^Tc]-hexamethylpropylene amine oxidase; IQR: Interquartile range; MMSE: Mini-mental state examination; MRI: Magnetic resonance imaging; MRS: Magnetic resonance spectroscopy; NMDA: N-methyl-d-aspartate; PD: Parkinson’s disease; PET: Positron emission tomography; REMARK: Reporting recommendations for tumor MARKer prognostic studies; SD: Standard deviation; SPECT: Single-photon emission computed tomography; UKPDS: UK Parkinson’s disease society; UPDRS: Unified Parkinson’s disease rating scale; UPDRS (I): Mention behaviour and mood subsection of the UPDRS; UPDRS (II): Activities of daily living subsection of the UPDRS; UPDRS (III): Motor subsection of the UPDRS

## Competing interests

There are no conflicts of interest for any of the authors in relation to this manuscript.

## Authors’ contributions

DJMM reviewed the abstracts identified by the electronic search, extracted data from the full text of articles, and carried out the hand search. DJMM also carried out all data analyses and wrote the paper. PLR designed the electronic search strategy and edited the manuscript. PAT and DEW provided statistical expertise with regards to the recommendations for future biomarker studies, and edited the manuscript. JPZ is the Principal Investigator for the overall programme grant, of which this study is a part, had input into study design, and edited the manuscript. CEC leads Parkinson’s disease aspects of the programme grant, had input into study design, supervised DJMM, and edited the manuscript. All authors read and approved the final manuscript.

## Pre-publication history

The pre-publication history for this paper can be accessed here:

http://www.biomedcentral.com/1471-2377/13/35/prepub

## Supplementary Material

Additional file 1 Electronic search strategy. Click here for file

Additional file 2 Data collection sheet. Click here for file

Additional file 3 Biomarkers examined in longitudinal studies and their relationship with clinical measures of disease severity. Click here for file

Additional file 4 Biomarkers examined in cross-sectional studies and their relationship with clinical measures of disease severity. Click here for file
