# Supplementary material for: A systematic review of biomarkers for disease progression in Parkinson’s disease
Source: BMC Neurol. 2013 Apr 12;13:35. doi: 10.1186/1471-2377-13-35 (PMC3637496; doi:10.1186/1471-2377-13-35)
Supplement: Additional file 1 — Electronic search strategy. [file 1471-2377-13-35-S1.docx]

# Additional file 1 Electronic search strategy

## 1 PD and Blood (MEDLINE and Embase)

1. (parkinson* disease or parkinsonism or parkinsonian).ti.
2. (blood or plasma or platelets).ti.
3. monamine oxidase.ti.
4. (phenylethylamine or lymphocyte* or oxidate stress or malondialdehyde or superoxide radical*).ti.
5. (coenzyme q10 or co-enzyme q10).ti.
6. hydroxyguanosine.ti.
7. (hydroxy adj guanosine).ti.
8. (copper or zinc superoxide dismutase).ti.
9. homovanillic.ti.
10. synuclein.tw.
11. fatty acid binding protein*.ti.
12. glutathione.ti.
13. (uric acid or urate).ti.
14. DNA.ti.
15. s100b.ti.
16. c-tau.ti.
17. neuron specific enolase.tw.
18. nse.ti.
19. noradrenaline.ti.
20. octopamine.ti.
21. (tyrosine hydroxylase or tyrosine decarboxylase).ti.
22. trace amines.ti.
23. 2 or 3 or 4 or 5 or 6 or 7 or 8 or 9 or 10 or 11 or 12 or 13 or 14 or 15 or 16 or 17 or 18 or 19 or 20 or 21 or 22
24. 1 and 23
25. (prognosis or progression).tw.
26. exp prognosis/
27. exp disease progression/
28. exp Biological Markers/
29. (biomarker* or bio-marker* or marker*).tw.
30. exp epidemiologic studies/
31. (cases and controls).ab.
32. ((matched and group*) or (control* and group*)).ab.
33. (cohort or prospective or retrospective or follow-up or longitudinal).tw.
34. 25 or 26 or 27 or 28 or 29 or 30 or 31 or 33
35. (mouse or mice or murine or rat or rats or animal or rodent or monkey or primate or drosophila).ti.
36. 34 not 35
37. 24 and 36

**Total retrieved = 931**

## 2 PD and Urine or CSF (MEDLINE and Embase)

1. (parkinson* disease or parkinsonism or parkinsonian).ti.
2. urine.ti.
3. isoprostaneF2alpha.ti.
4. (isoprostane adj F2alpha).ti.
5. cerebrospinal fluid.ti.
6. csf.ti.
7. hydroxyguanosine.ti.
8. (hydroxy adj deoxy adj guanosine).ti.
9. (hydroxy adj guanosine).ti.
10. Malondialdehyde.ti.
11. phenylethylamine.ti.
12. orexin.ti.
13. indoleacetic acid.ti.
14. Acetylcholinesterase.ti.
15. Ache.ti.
16. (dopamine adj metabolite*).ti.
17. complex 1.ti.
18. (oxidative stress adj marker*).ti.
19. hydroxydeoxyguanosine.ti.
20. 2 or 3 or 4 or 5 or 6 or 7 or 8 or 9 or 10 or 11 or 12 or 13 or 14 or 15 or 16 or 17 or 18 or 19
21. 1 and 20
22. (mouse or mice or murine or rat or rats or animal or rodent or monkey or primate or drosophila).ti.
23. 21 not 22

**Total retrieved = 571**

## 3 PD and Imaging (MEDLINE and Embase)

1. (parkinson* disease or parkinsonism or parkinsonian).ti.
2. imaging.ti.
3. neuroimaging.ti.
4. ultrasound.ti.
5. ultrasonography.ti.
6. sonography.ti.
7. ultrasound.ti.
8. serotonin.tw.
9. nicotine.ti.
10. mibg.ti.
11. Metaiodobenzylguanidine.ti.
12. tomography.ti.
13. (TCS or CT or MRI).ti.
14. (HRRT or spect or pib-pet).ti.
15. FDG.ti.
16. amyloid.ti.
17. (hyperechogenicity or Echogenicity).ti.
18. interleukin.ti.
19. IL.ti.
20. tGF.ti.
21. (tumor growth factor or tumour growth factor).ti.
22. tnf.ti.
23. spectroscopy.ti.
24. Neuromelanin.ti.
25. Metalloproteinase.ti.
26. spectroscopy.ti.
27. ((nicotinic or d2 or opioid) and receptor*).ti.
28. 2 or 3 or 4 or 5 or 6 or 7 or 8 or 9 or 10 or 11 or 12 or 13 or 14 or 15 or 16 or 17 or 18 or 19 or 20 or 21 or 22 or 23 or 24 or 25 or 26 or 27
29. 1 and 28
30. (prognosis or progression).tw.
31. exp prognosis/
32. exp disease progression/
33. exp Biological Markers/
34. (biomarker* or bio-marker* or marker*).tw.
35. exp epidemiologic studies/
36. (cases and controls).ab.
37. ((matched and group*) or (patients and control*) or (control* and group*)).ab.
38. (cohort or prospective or retrospective or follow-up or longitudinal).tw.
39. 30 or 31 or 32 or 33 or 34 or 35 or 36 or 38
40. 29 and 39
41. (mouse or mice or murine or rat or rats or animal or rodent or monkey or primate or drosophila).ti.
42. 40 not 41

**Total retrieved = 1068**

## 4 PD and Neurophysiology (MEDLINE and Embase)

1. (parkinson* disease or parkinsonism or parkinsonian).ti.
2. EMG.ti.
3. Electromyography.ti.
4. EEG.ti.
5. ECG.ti.
6. Electroencephalography.ti.
7. Electrocardiogram.ti.
8. Accelerometer.ti.
9. Transcranial magnetic stimulation.ti.
10. "Somatosensory evoked potential*".ti.
11. proteomics.ti.
12. metabolomics.ti.
13. neurochemical.ti.
14. transcriptomics.ti.
15. 2 or 3 or 4 or 5 or 6 or 7 or 8 or 9 or 10 or 11 or 12 or 13 or 14
16. 1 and 15
17. (mouse or mice or murine or rat or rats or animal or rodent or monkey or primate or drosophila).ti.
18. 16 not 17

**Total retrieved = 496**

## 5. PD and Biomarkers

### 5.1 MEDLINE

1. *Parkinson Disease/bl, cf, ri, us, ur [Blood, Cerebrospinal Fluid, Radionuclide Imaging, Ultrasonography, Urine]
2. (prognosis or progression).tw.
3. *prognosis/
4. exp disease progression/
5. (cases and controls).ab.
6. ((matched and group*) or (patients and control*) or (control* and group*)).ab.
7. (cohort or prospective or retrospective or follow-up or longitudinal).tw.
8. 2 or 3 or 4 or 5 or 6 or 7
9. 1 and 8
10. exp Parkinson Disease/
11. "parkinson* disease".ti.
12. 10 or 11
13. exp Biological Markers/
14. (biomarker * or bio-marker* or marker*).ti.
15. 13 or 14
16. exp Disease Progression/
17. progression.tw.
18. 16 or 17
19. 12 and 15 and 18
20. 9 or 19
21. (mouse or mice or murine or rat or rats or animal or rodent or monkey or primate or drosophila).ti.
22. 20 not 21

**Total retrieved = 751**

### 5.2 Embase

1. *Parkinson Disease/
2. (parkinson* disease or parkinsonism or parkinsonian).ti.
3. 1 or 2
4. exp Biological Markers/
5. (biomarker* or marker* or bio-marker*).ti.
6. 4 or 5
7. Disease course/
8. progression.tw.
9. 7 or 8
10. 3 and 6 and 9
11. (mouse or mice or murine or rat or rats or animal or rodent or monkey or primate or drosophila).ti.
12. exp longitudinal study/
13. exp follow up/
14. (progression or prognosis).tw.
15. 12 or 13
16. 2 and 14 and 15
17. 10 or 16
18. 17 not 11

**Total retrieved = 263**
