# Supplementary material for: A systematic review of biomarkers for disease progression in Parkinson’s disease
Source: BMC Neurol. 2013 Apr 12;13:35. doi: 10.1186/1471-2377-13-35 (PMC3637496; doi:10.1186/1471-2377-13-35)
Supplement: Additional file 2 — Data collection sheet. [file 1471-2377-13-35-S2.pdf]

Record No.		Collector	Date

General Information

Article Title	
Author	
Journal	
Citation	
Country	
Source of Funding	
How PD Diagnosed?	

Participant Numbers

	PD Group	Control Group
Number Eligible		
Number Enrolled		
Number of Withdrawals		
Number of Exclusions		
Number Lost to FU		

Study Characteristics

Aims and Objectives<br>Study Design	
Inclusion/ Exclusion Criteria	
Recruitment (including randomization)	

Participant Baseline Characteristics

		PD Group
Age		
Sex	Male	
	Female	
Disease Characteristics or Stage		
Treated		
Untreated		

Intervention

Record No.	

Description of test performed	
How often repeated and time periods	
Theoretical basis	

Outcome Data *(correlation with impairment, handicap, disability, survival and statistic analysis used)*

Outcome Data *(influence of drug therapy on marker)*

•Details of any economical analysis

Record No.	

•Details of any assessment of test suitability and acceptability to the patient

Quality Questionnaire

(Based on REMARK [Reporting recommendations for tumour MARKer prognostic studies])

(1) Was the study prospective?

YES	The study reports that patients and the performed test result were collected before the development of an outcome.
NO	No report or clearly retrospective (eg, patients with poor prognosis collected before biomarker measurement).

(2) Was evaluation of prognostic marker blinded to patient outcome?

YES	The study reports an attempt to blind the person measuring the level of biomarker to patient outcome.
NO	There is no such report.

(3) Was there a defined time period during which patients were enrolled?

YES	Study define time period, end of follow-up period, and median follow-up time.
NO	Does not define above criteria.

(4) Were there precisely defined clinical outcomes at the start of the study?

YES	Study defines which clinical end points are to be measured.
NO	No such definition.

(5) Were the methods for measuring the prognostic marker adequately described and referenced?

YES	
NO	

(6) Cases unselected/unbiased?

YES	No attempt to select patients with exclusion criteria
NO	Only a subset of patients with Parkinson’s disease enter the study
