# Supplementary material for: A systematic review of biomarkers for disease progression in Parkinson’s disease
Source: BMC Neurol. 2013 Apr 12;13:35. doi: 10.1186/1471-2377-13-35 (PMC3637496; doi:10.1186/1471-2377-13-35)
Supplement: Additional file 3 — Biomarkers examined in longitudinal studies and their relationship with clinical measures of disease severity. [file 1471-2377-13-35-S3.docx]

**Additional file 3 Longitudinal studies**

**Associations between putative biomarkers and clinical measures of disease severity, in longitudinal studies included in the systemic review of biomarkers for disease progression in Parkinson’s disease**

| **Contents** | | |
| --- | --- | --- |
|  |  |  |
| Brain PET | 2 |  |
| Brain SPECT | 4 |  |
| Brain MRI | 6 |  |
| Electrophysiology | 7 |  |
| Other | 7 |  |
| Overall key | 8 |  |
| References | 10 |  |

| **PET biomarkers – Putamen (PU)** | | | |  |  | **Association of change in ligand binding with change in:** | | | | | |
| --- | --- | --- | --- | --- | --- | --- | --- | --- | --- | --- | --- |
| **PET ligand** | **Region in which change in binding measured** | **Reference**  **(first author, year)** | **n at baseline** | **Number of scans** | **Time between first and last scan (years)** | **Total UPDRS** | **UPDRS (III)** | **UPDRS (II)** | **H&Y** | **MMSE** | **Total CAMCOG** |
| FDOPA | PU (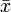) | Morrish*,* 1996 [1] | 17 | 2 | 1.4 | NSA | NSA |  |  |  |  |
|  | PU (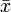)† | Morrish*,* 1998 [2] | 32 | 2 | 1.5 | NSA |  |  |  |  |  |
|  | PU (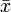) normalising ratio† | Morrish*,* 1998 [2] | 32 | 2 | 1.5 | NSA |  |  |  |  |  |
| [^18^F]FP-CIT | PU (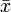) | Huang*,* 2007 [3] | 15 | 3 | 3.9 |  | r = -0.59** |  |  |  |  |
| [^18^F]CFT | Ant PU (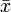) | Nurmi*,* 2003 [4] | 12 | 2 | 2.2 |  | NSA |  |  |  |  |
|  | Post PU (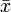) | Nurmi*,* 2003 [4] | 12 | 2 | 2.2 |  | NSA |  |  |  |  |

| **PET biomarkers – Caudate Nucleus (CN)** | | | | | | **Association of change in ligand binding with change in:** | | | | | |
| --- | --- | --- | --- | --- | --- | --- | --- | --- | --- | --- | --- |
| **PET ligand** | **Region in which change in binding measured** | **Reference**  **(first author, year)** | **n at baseline** | **Number of scans** | **Time between first and last scan (years)** | **Total UPDRS** | **UPDRS (III)** | **UPDRS (II)** | **H&Y** | **MMSE** | **Total CAMCOG** |
| FDOPA | CN (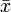)†  CN (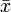) normalising ratio† | Morrish*,*1998 [2] | 32 | 2 | 1.5 | NSA |  |  |  |  |  |
|  |  | Morrish*,*1998 [2] | 32 | 2 | 1.5 | NSA |  |  |  |  |  |
| [^18^F]FP-CIT | CN (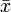) | Huang*,* 2007 [3] | 15 | 3 | 3.9 |  | r = -0.60** |  |  |  |  |
| [^18^F]CFT | CN (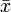) | Nurmi*,* 2003 [4] | 12 | 2 | 2.2 |  | NSA |  |  |  |  |
|  | | | | | |  | | | | | |

| **PET biomarkers – Striatum (ST)** | | | | | | **Association of change in ligand binding with change in:** | | | | | |
| --- | --- | --- | --- | --- | --- | --- | --- | --- | --- | --- | --- |
| **PET ligand** | **Region in which change in binding measured** | **Reference**  **(first author, year)** | **n at baseline** | **Number of scans** | **Time between first and last scan (years)** | **Total UPDRS** | **UPDRS (III)** | **UPDRS (II)** | **H&Y** | **MMSE** | **Total CAMCOG** |
| FDOPA | ST (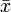) | Schweitzer*,* 2006 [5] | 16 | 2 | 5.5 |  | NSA |  |  |  |  |
|  |  | Morrish*,*1998 [2] | 32 | 2 | 1.5 | NSA |  |  |  |  |  |
|  | ST | Bruck*,* 2006 [6] | 31 | 2 | 2.1 |  | NSA |  |  |  |  |
|  |  | Bruck*,* 2009 [7] | 16 | 3 | 5.5 |  | NSA |  |  |  |  |

| **PET biomarkers – Other** | | | | | | **Association of change in ligand binding with change in:** | | | | | |
| --- | --- | --- | --- | --- | --- | --- | --- | --- | --- | --- | --- |
| **PET ligand** | **Region in which change in binding measured** | **Reference**  **(first author, year)** | **n at baseline** | **Number of scans** | **Time between first and last scan (years)** | **Total UPDRS** | **UPDRS (III)** | **UPDRS (II)** | **H&Y** | **MMSE** | **Total CAMCOG** |
| FDG | PD-related cognitive pattern (PDCP) | Huang*,* 2007 [3] | 15 | 3 | 3.9 |  | NSA |  |  |  |  |
|  | PD-related spatial covariance pattern (PDRP) | Huang*,* 2007 [3] | 15 | 3 | 3.9 |  | r = 0.62** |  |  |  |  |
|  | Subnetwork expression  Positive subnetwork | Ma*,* 2009 [8] | 15 | 3 | 3.9 |  | r = 0.62** |  |  |  |  |
|  | Negative subnetwork | Ma*,* 2009 [8] | 15 | 3 | 3.9 |  | r = 0.53* |  |  |  |  |
|  | Early network | Ma*,* 2009 [8] | 15 | 3 | 3.9 |  | r = 0.59** |  |  |  |  |
|  | Global metabolic rate | Ma*,* 2009 [8] | 15 | 3 | 3.9 |  | r = -0.32◘ |  |  |  |  |

| **SPECT biomarkers – Putamen (PU)** | | | |  |  | **Association of change in ligand binding with change in:** | | | | | |
| --- | --- | --- | --- | --- | --- | --- | --- | --- | --- | --- | --- |
| **SPECT ligand** | **Region in which change in binding measured** | **Reference**  **(first author, year)** | **n at baseline** | **Number of scans** | **Time between first and last scan (years)** | **Total UPDRS** | **UPDRS (III)** | **UPDRS (II)** | **H&Y** | **MMSE** | **Total CAMCOG** |
| [^123^I]FP-CIT | Ant PU (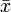) | Colloby*,* 2005 [9] | 20‡ | 2 | 1 |  | NSA |  |  |  | r = -0.40◘ |
|  |  | Colloby*,* 2005 [9] | 15‡ | 2 | 1 |  | r = -0.52◘ |  |  |  | NSA |
|  | Post PU (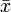) | Colloby*,* 2005 [9] | 20‡ | 2 | 1 |  | r = -0.07◘ |  |  |  | NSA |
|  |  | Colloby*,* 2005 [9] | 15‡ | 2 | 1 |  | r = 0.17◘ |  |  |  | NSA |
| [^123^I]β-CIT | PU | Marek*,* 2001 [10] | 32 | 2§ | 2.3 | NSA | NSA |  |  |  |  |
|  |  | PSG, 2002 [11] | 82 | 2• | 1.8 | r = -0.03◘ |  |  |  |  |  |
|  |  | PSG, 2002 [11] | 82 | 2• | 2.8 | r = -0.39** |  |  |  |  |  |
|  |  | PSG, 2002 [11] | 82 | 2• | 3.8 | r = -0.39** |  |  |  |  |  |

| **SPECT biomarkers – Caudate nucleus (CN)** | | | |  |  | **Association of change in ligand binding with change in:** | | | | | |
| --- | --- | --- | --- | --- | --- | --- | --- | --- | --- | --- | --- |
| **SPECT ligand** | **Region in which change in binding measured** | **Reference**  **(first author, year)** | **n at baseline** | **Number of scans** | **Time between first and last scan (years)** | **Total UPDRS** | **UPDRS (III)** | **UPDRS (II)** | **H&Y** | **MMSE** | **Total CAMCOG** |
| [^123^I]FP-CIT | CN (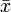) | Colloby*,* 2005 [9] | 20‡ | 2 | 1 |  | NSA |  |  |  | NSA |
|  |  | Colloby*,* 2005 [9] | 15‡ | 2 | 1 |  | r =-0.71** |  |  |  | r = 0.13◘ |
| [^123^I]β-CIT | CN | Marek*,* 2001 [10] | 32 | 2§ | 2.3 | NSA | NSA |  |  |  |  |
|  |  | PSG, 2002 [11] | 82 | 2• | 1.8 | r = 0.02◘ |  |  |  |  |  |
|  |  | PSG, 2002 [11] | 82 | 2• | 2.8 | r = -0.20◘ |  |  |  |  |  |
|  |  | PSG, 2002 [11] | 82 | 2• | 3.8 | r = -0.35◘ |  |  |  |  |  |

| **SPECT biomarkers – Striatum (ST)** | | | | |  | **Association of change in ligand binding with change in:** | | | | | |
| --- | --- | --- | --- | --- | --- | --- | --- | --- | --- | --- | --- |
| **SPECT ligand** | **Region in which change in binding measured** | **Reference**  **(first author, year)** | **n at baseline** | **Number of scans** | **Time between first and last scan (years)** | **Total UPDRS** | **UPDRS (III)** | **UPDRS (II)** | **H&Y** | **MMSE** | **Total CAMCOG** |
| [^123^I]FP-CIT | ST (Whole) | Winogrodzka*,* 2001 [12] | 20 | 2 | 1.06 |  | NSA |  |  |  |  |
|  | Ipsilateral ST | Winogrodzka*,* 2001 [12] | 20 | 2 | 1.06 |  | NSA |  |  |  |  |
|  | Contralateral ST | Winogrodzka*,* 2001 [12] | 20 | 2 | 1.06 |  | NSA |  |  |  |  |
| [^123^I]β-CIT | ST (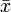) | Pirker*,* 2002 [13] | 36 | 2 | 2.16 |  | r = -0.10 ◘ | r = 0.11◘ | r = 0.14◘ |  |  |
|  | ST | Marek*,* 2001 [10] | 32 | 2§ | 2.3 | NSA | NSA |  |  |  |  |
|  |  | Pirker*,* 2003 [14] | 21 ^Φ^ | 3 | 5.3 |  | NSA | NSA | NSA |  |  |
|  |  | PSG, 2002 [11] | 82 | 2• | 1.8 | r = -0.01◘ |  |  |  |  |  |
|  |  | PSG, 2002 [11] | 82 | 2• | 2.8 | r = -0.30* |  |  |  |  |  |
|  |  | PSG, 2002 [11] | 82 | 2• | 3.8 | r = -0.40** |  |  |  |  |  |
| HMPAO | ST | Firbank*,* 2005 [15] | 36 | 2 | 1.03 |  | POS |  |  |  |  |

| **SPECT biomarkers – Other** | | | | | | **Association of change in ligand binding with change in:** | | | | | |
| --- | --- | --- | --- | --- | --- | --- | --- | --- | --- | --- | --- |
| **SPECT ligand** | **Region in which change in binding measured** | **Reference**  **(first author, year)** | **n at baseline** | **Number of scans** | **Time between first and last scan (years)** | **Total UPDRS** | **UPDRS (III)** | **UPDRS (II)** | **H&Y** | **MMSE** | **Total CAMCOG** |
| [^123^I]IMP | Parietal cortex | Tachibana*,* 1995 [16] | 30 | 2 | 1 |  |  |  |  | r = 0.58*** |  |
| HMPAO | SPM99 map of areas of reduced cerebral perfusion over one year | Firbank*,* 2005 [17] | 38 | 2 | 1.06 |  | NSA |  |  |  | NSA |
|  | Cerebellar vermis | Firbank*,* 2005 [15] | 36 | 2 | 1.06 |  | POS |  |  |  |  |

| **Magnetic Resonance Imaging (MRI) biomarkers** | | | | | | **Association of change in ligand binding with change in:** | | | | | |
| --- | --- | --- | --- | --- | --- | --- | --- | --- | --- | --- | --- |
| **Modality** | **Change in feature measured** | **Reference**  **(first author, year)** | **n at baseline** | **Number of scans** | **Time between first and last scan (Years)** | **Total UPDRS** | **UPDRS (III)** | **UPDRS (II)** | **H&Y** | **MMSE** | **Total CAMCOG** |
| T1-weighted 3D volumetric MRI | Shift in brain/CSF boundary (measure of brain atrophy rate) | Burton*,* 2005 [18] | 18^¥^ | 2 | 1.1 |  |  |  |  |  | NSA |
|  |  | Burton*,* 2005 [18] | 13 ^¥^ | 2 | 1.1 |  |  |  |  |  | NSA◘ |

| **Electrophysiological biomarkers** | |  |  |  |  | **Association of change in feature measured with change in:** | | | | | |
| --- | --- | --- | --- | --- | --- | --- | --- | --- | --- | --- | --- |
| **Modality** | **Feature measured** | **Reference**  **(first author, year)** | **n at baseline** | **Number of time points** | **Time between first and last time points (years)** | **Total UPDRS** | **UPDRS (III)** | **UPDRS (II)** | **H&Y** | **MMSE** | **Total CAMCOG** |
| Electroencephalography (EEG) all night polysomnographic study | Phasic activity | Garcia-Borreguero*,* 2002 [19] | 15 | 2 | 0.50 | NSA | NSA |  |  |  |  |
|  | Tonic activity | Garcia-Borreguero*,* 2002 [19] | 15 | 2 | 0.50 | NSA | NSA |  |  |  |  |

| **Other biomarkers** | |  |  |  |  | **Association of change in feature measured with change in:** | | | | | |
| --- | --- | --- | --- | --- | --- | --- | --- | --- | --- | --- | --- |
| **Modality** | **Feature measured** | **Reference**  **(first author, year)** | **n at baseline** | **Number of time points** | **Time between first and last time points (years)** | **Total UPDRS** | **UPDRS (III)** | **UPDRS (II)** | **H&Y** | **MMSE** | **Total CAMCOG** |
| Acoustical analysis following four sentence reading task | Mean fundamental frequency | Skodda*,* 2009 [20] | 27ф | 2 | 2.1 |  | NSA |  |  |  |  |
|  |  | Skodda*,* 2009 [20] | 23ф | 2 | 2.1 |  | NSA |  |  |  |  |
|  | Fundamental frequency  variation range | Skodda*,* 2009 [20] | 27ф | 2 | 2.1 |  | NSA |  |  |  |  |
|  |  | Skodda*,* 2009 [20] | 23ф | 2 | 2.1 |  | NSA |  |  |  |  |
|  | Total speech rate | Skodda*,* 2009 [20] | 27ф | 2 | 2.1 |  | NSA |  |  |  |  |
|  |  | Skodda*,* 2009 [20] | 23ф | 2 | 2.1 |  | NSA |  |  |  |  |
|  | Net speech rate | Skodda*,* 2009 [20] | 27ф | 2 | 2.1 |  | NSA |  |  |  |  |
|  |  | Skodda*,* 2009 [20] | 23ф | 2 | 2.1 |  | NSA |  |  |  |  |
|  | Percentual pause ratio | Skodda*,* 2009 [20] | 27ф | 2 | 2.1 |  | NSA |  |  |  |  |
|  |  | Skodda*,* 2009 [20] | 23ф | 2 | 2.1 |  | NSA |  |  |  |  |
|  | Ratio of intra-word | Skodda*,* 2009 [20] | 27ф | 2 | 2.1 |  | NSA |  |  |  |  |
|  | pauses | Skodda*,* 2009 [20] | 23ф | 2 | 2.1 |  | NSA |  |  |  |  |

**Key**

Numbers in square brackets correspond to the list of references

† 13 of the patients with Parkinson’ disease were given the peripheral catechol-O-methyltransferase inhibitor entacapone one hour before each of their scans. When analysing the scans, results from left and right hemispheres were averaged. A normal mean was calculated for each structure, however entacapone changes the influx constant and the ratio calculated using occipital counts as input function. Therefore the results of the 13 patients scanned with entacapone as premedication were normalised by multiplying them by the ratio of the normal mean with entacapone to the normal mean without entacapone.

‡ Study examined patients with Parkinson’s disease (n=20) and Parkinson’s disease with dementia (n=15) separately

§ Paper states that 15 Parkinson’s disease patients had more than 2 scans

• In this longitudinal study patients underwent 4 scans: (1) Baseline, (2) 22 months, (3) 34 months, and (4) 46 months. The paper gives correlation coefficients for the change in uptake between the baseline scan and each subsequent scan separately.

^Φ^ This study is an extension of Pirker, 2002, in which 24 early stage PD patients were scanned twice. Of these early stage patients 21 were scanned for a third time in this follow up study, and longitudinal data analysed for those 21 patients.

¥ Study examined patients with Parkinson’s disease (n=18) and Parkinson’s disease with dementia (n=13) separately.

ф Study examined for Associations between each factor and UPDRS (III) in males (n=27) and females (n=23) separately.


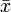
 Where this symbol is show then the value given is the average of left and right hemispheric structures. If not shown then it is unclear from the text whether the value represents an average or a total (left and right hemispheric structures combined) value.

**Correlations**

In many cases it was unclear whether a given correlation coefficient was a Pearson’s or Spearman’s correlation coefficient. Therefore, for simplicity all correlation coefficients are simply denoted by ‘r’.

NSA No significant association No symbol: P not significant, but actual value not stated

POS Significant positive association ◘ P ≥ 0.05

NEG Significant negative association ^(^*^)^ P significant, but actual value not stated

* P < 0.05

** P < 0.01

*** P < 0.001

**Clinical Rating Scales**

H&Y Hoehn and Yahr staging scale [21]

MMSE Mini-Mental State Examination [22]

Total CAMCOG The cognitive and self-contained part of the Cambridge Examination for Mental disorders of the Elderly [23]

Total UPDRS Total score derived from the Unified Parkinson’s Disease Rating Scale [24]

UPDRS (II) Activities of daily living component of Unified Parkinson’s Disease Rating Scale

UPDRS (III) Motor component of Unified Parkinson’s Disease Rating Scale

**Areas of the brain**

CN Caudate Nucleus

PU Putamen

ST Striatum

**PET ligands**

[^18^F]CFT 2β-carbomethoxy-3β-(4-[^18^F])-fluorophenyl)tropane

[^18^F]FP-CIT [^18^F]-2β-carbomethoxy-3β-(4-iodophenyl)-N-(3-fluoropropyl)-N-tropane

FDG [^18^F]-2-fluoro-2-deoxyglucose

FDOPA [^18^F]6-fluoro-L-3,4-dihydroxyphenylalanine

**SPECT ligands**

[^123^I]FP-CIT [^123^I]-2β-carbomethoxy-3β-(4-iodophenyl)-N-(3-fluoropropyl)-N-tropane

[^123^I]IMP N-isopropyl-P[^123^I]-iodoamphetamine

[^123^I]β-CIT [^123^I]-2β-carbomethoxy-3β-(4-iodophenyl tropane)

HMPAO [^99m^Tc]-hexamethylpropylene amine oxid

**References**

1. Morrish PK, Sawle GV, Brooks DJ: **An [18F]dopa-PET and clinical study of the rate of progression in Parkinson's disease.** *Brain* 1996, **119(2):**585-591.

2. Morrish PK, Rakshi JS, Bailey DL, Sawle GV, Brooks DJ: **Measuring the rate of progression and estimating the preclinical period of Parkinson's disease with [18F]dopa PET.** *J Neurol Neurosurg Psychiatry* 1998, **64(3):**314-319.

3. Huang C, Tang C, Feigin A, Lesser M, Ma Y, Pourfar M, Dhawan V, Eidelberg D: **Changes in network activity with the progression of Parkinson's disease.** *Brain* 2007, **130(7)**:1834-1846.

4. Nurmi E, Bergman J, Eskola O, Solin O, Vahlberg T, Sonninen P, Rinne JO: **Progression of dopaminergic hypofunction in striatal subregions in Parkinson's disease using [18F]CFT PET.** *Synapse* 2003, **48(3):**109-115.

5. Schweitzer KJ, Hilker R, Walter U, Burghaus L, Berg D: **Substantia nigra hyperechogenicity as a marker of predisposition and slower progression in Parkinson's disease.** *Mov Disord*  2006, **21(1):**94-98.

6. Bruck A, Aalto S, Nurmi E, Vahlberg T, Bergman J, Rinne JO: **Striatal subregional 6-[18F]fluoro-L-dopa uptake in early Parkinson's disease: a two-year follow-up study.** *Mov Disord*  2006, **21(7):**958-963.

7. Bruck A, Aalto S, Rauhala E, Bergman J, Marttila R, Rinne JO: **A follow-up study on 6-[18F]Fluoro-L-dopa uptake in early Parkinson's disease shows nonlinear progressionin the putamen.** *Mov Disord*  2009, **24(7):**1009-1015.

8. Ma Y, Tang C, Moeller JR, Eidelberg D: **Abnormal regional brain function in Parkinson's disease: truth or fiction?** *Neuroimage* 2009, **45(2):**260-266.

9. Colloby SJ, Williams ED, Burn DJ, Lloyd JJ, McKeith IG, O'Brien JT: **Progression of dopaminergic degeneration in dementia with Lewy bodies and Parkinson's disease with and without dementia assessed using 123I-FP-CIT SPECT.** *Eur J Nucl Med Mol Imaging* 2005, **32(10):**1176-1185.

10. Marek K, Innis R, van DC, Fussell B, Early M, Eberly S, Oakes D, Seibyl J: **[123I]beta-CIT SPECT imaging assessment of the rate of Parkinson's disease progression.** *Neurology* 2001, **57(11):**2089-2094.

11. Parkinson Study Group: **Dopamine transporter brain imaging to assess the effects of pramipexole vs levodopa on Parkinson disease progression.** *JAMA* 2002, **287(13):**1653-1661.

12. Winogrodzka A, Bergmans P, Booij J, van Royen EA, Janssen AG, Wolters EC: **[123I]FP-CIT SPECT is a useful method to monitor the rate of dopaminergic degeneration in early-stage Parkinson's disease.** *J Neural Transm* 2001, **108(8-9):**1011-1019.

13. Pirker W, Djamshidian S, Asenbaum S, Gerschlager W, Tribl G, Hoffmann M, Brucke T: **Progression of dopaminergic degeneration in Parkinson's disease and atypical parkinsonism: a longitudinal beta-CIT SPECT study.** *Mov Disord*  2002, **17(1):**45-53.

14. Pirker W, Holler I, Gerschlager W, Asenbaum S, Zettinig G, Brucke T: **Measuring the rate of progression of Parkinson's disease over a 5-year period with beta-CIT SPECT.** *Mov Disord*  2003, **18(11):**1266-1272.

15. Firbank MJ, Burn DJ, McKeith IG, O'Brien JT: **Longitudinal study of cerebral blood flow SPECT in Parkinson's disease with dementia, and dementia with Lewy bodies.** *Int J Geriatr Psychiatry* 2005, **20(8):**776-782.

16. Tachibana H, Tomino Y, Kawabata K, Sugita M, Fukuchi M: **Twelve-month follow-up study of regional cerebral blood flow in Parkinson's disease.** *Dementia* 1995, **6(2):**89-93.

17. Firbank MJ, Molloy S, McKeith IG, Burn DJ, O'Brien JT: **Longitudinal change in 99mTcHMPAO cerebral perfusion SPECT in Parkinson's disease over one year.** *J Neurol Neurosurg Psychiatry* 2005, **76(10):**1448-1451.

18. Burton EJ, McKeith IG, Burn DJ, O'Brien JT: **Brain atrophy rates in Parkinson's disease with and without dementia using serial magnetic resonance imaging.** *Mov Disord*  2005, **20(12):**1571-1576.

19. Garcia-Borreguero D, Caminero AB, De La Llave Y, Larrosa O, Barrio S, Granizo JJ, Pareja JA: **Decreased phasic EMG activity during rapid eye movement sleep in treatment-naive Parkinson's disease: effects of treatment with levodopa and progression of illness.** *Mov Disord*  2002, **17(5):**934-941.

20. Skodda S, Rinsche H, Schlegel U: **Progression of dysprosody in Parkinson's disease over time - A longitudinal study.** *Mov Disord*  2009, **24(5):**716-722.

21. Goetz CG, Poewe W, Rascol O, Sampaio C, Stebbins GT, Counsell C, Giladi N, Holloway RG, Moore CG, Wenning GK, Yahr MD, Seidl L: **Movement Disorder Society Task Force report on the Hoehn and Yahr staging scale: status and recommendations.** *Mov Disord* 2004, **19(9)**:1020-1028.

22. Folstein MF, Folstein SE, McHugh PR: **"Mini-mental state". A practical method for grading the cognitive state of patients for the clinician.** *J Psychiatr Res* 1975, **12(3):**189-198.

23. Roth M, Tym E, Mountjoy CQ, Huppert FA, Hendrie H, Verma S, Goddard R: **CAMDEX. A standardised instrument for the diagnosis of mental disorder in the elderly with special reference to the early detection of dementia.** *Br J Psychiatry* 1986, **149**:698-709.

24. Fahn S, Eton RL, UPDRS Development Committee: **The Unified Parkinson's Disease Rating Scale.** In *Recent Developments in Parkinson's Disease. Volume 2.* Edited by Fahn S, Marsden CD, Goldstein M, Calne DB. Florham Park, New Jersey: Macmillan Healthcare Information; 1987:153-163.
