# Supplementary material for: A systematic review of biomarkers for disease progression in Parkinson’s disease
Source: BMC Neurol. 2013 Apr 12;13:35. doi: 10.1186/1471-2377-13-35 (PMC3637496; doi:10.1186/1471-2377-13-35)
Supplement: Additional file 4 — Biomarkers examined in cross-sectional studies and their relationship with clinical measures of disease severity. [file 1471-2377-13-35-S4.docx]

**Additional file 4 Cross-sectional studies**

**Associations between putative blood, plasma and serum biomarkers and clinical measures of disease severity, in**

**cross-sectional studies included in the systemic review of biomarkers for disease progression in Parkinson’s disease**

| **Contents** | | | |
| --- | --- | --- | --- |
|  | |  |  |
| 1: Blood/plasma/serum | 2 |  |  |
| 2: Cerebrospinal fluid (CSF) | 13 |  |  |
| 3: Urine | 19 |  |  |
| 4: Cranial ultrasound | 20 |  |  |
| 5: Cardiac 123I-MIBG scintigraphy | 23 |  |  |
| 6: Brain MRI and Brain MRS | 24 |  |  |
| 7: Brain SPECT | 33 |  |  |
| 8: Brain PET | 43 |  |  |
| 9: Electrophysiology | 51 |  |  |
| 10: Other | 55 |  |  |
| 11: Overall key | 56 |  |  |
| References | | 58 |  |

**Additional file 3  *1: Blood/plasma/serum***

**Associations between putative blood, plasma and serum biomarkers and clinical measures of disease severity, in**

**cross-sectional studies included in the systemic review of biomarkers for disease progression in Parkinson’s disease**

| **1.1 Structural proteins** | | | **Association of concentration of substance measured with:** | | | | | | | | | |
| --- | --- | --- | --- | --- | --- | --- | --- | --- | --- | --- | --- | --- |
| **Substance measured** | **Reference**  **(first author, year)** | **n** | **Total UPDRS** | **UPDRS**  **(III)** | **UPDRS**  **(II)** | **UPDRS**  **(I)** | **H&Y** | **MMSE** | **Blessed IMC** | **DRS** | **CURS** | **S&E** |
| Alpha-synuclein | Shi*,* 2010^101^ | 117 |  |  |  |  | NSA |  |  |  |  |  |
| DJ-1 | Shi*,* 2010^101^ | 117 |  |  |  |  | NSA |  |  |  |  |  |
|  | Waragai*,* 2007^102^ | 104 |  |  |  |  | Level higher in H&Y 3-4 than 1-2 |  |  |  |  |  |

| **1.2 Neurotransmitters** | | | **Association of concentration of substance measured with:** | | | | | | | | | |
| --- | --- | --- | --- | --- | --- | --- | --- | --- | --- | --- | --- | --- |
| **Substance measured** | **Reference**  **(first author, year)** | **n** | **Total UPDRS** | **UPDRS**  **(III)** | **UPDRS**  **(II)** | **UPDRS**  **(I)** | **H&Y** | **MMSE** | **Blessed IMC** | **DRS** | **CURS** | **S&E** |
| Dihydroxyphenylacetic acid (DOPAC) | Eldrup*,* 1995^103^ | 16 |  |  |  |  | NSA |  |  |  |  |  |
| 3,4-dihydroxyphenylalanine (DOPA) | Eldrup*,* 1995^103^ | 16 |  |  |  |  | NSA |  |  |  |  |  |
| Dopamine | Eldrup*,* 1995^103^ | 16 |  |  |  |  | NSA |  |  |  |  |  |

| **1.3 Free-radicals** | | | **Association of concentration of substance measured with:** | | | | | | | | | |
| --- | --- | --- | --- | --- | --- | --- | --- | --- | --- | --- | --- | --- |
| **Substance measured** | **Reference**  **(first author, year)** | **n** | **Total UPDRS** | **UPDRS**  **(III)** | **UPDRS**  **(II)** | **UPDRS**  **(I)** | **H&Y** | **MMSE** | **Blessed IMC** | **DRS** | **CURS** | **S&E** |
| Erythrocyte Glutathione peroxidise (GPx) | Chen*,* 2009 [4] | 211 |  |  |  |  | NSA |  |  |  |  |  |
| Glutathione reductase activity (erythrocyte haemolysate) | Ilic, 1999 [5] | 34 |  |  |  |  | NSA |  |  |  |  |  |
| High sensitivity C-reactive protein (Hs-CRP) | Seet*,* 2010 [6] | 61 |  |  |  |  | POS |  |  |  |  |  |
|  | Song*,* 2009 [7] | 212 |  |  |  |  | No significant difference (H&Y 1, 2, 3 or 4) |  |  |  |  |  |
| Hydroxyeicosatetraenoic acid products (HETEs) | Seet*,* 2010 [6] | 61 |  |  |  |  | NEG |  |  |  |  |  |
| Hydroxyl radical | Ihara*,* 1999 [8] | 48 |  |  |  |  | Level higher in H&Y 4-5 than 1-3 |  |  |  |  |  |
| Isoprostane iP2_α_-IV (F2A) | Irizarry*,* 2007 [9] | 47 |  |  |  |  |  |  | NSA |  |  |  |
| 8,12-isoprostaneF_2α_-VI (iP) | Connolly*,* 2008 [10] | 36 |  | NSA | NSA |  |  | NSA |  |  |  |  |
| F2-isoprostanes (F2-IsoPs) [includes 8-OHdG] | Seet*,* 2010 [6] | 61 |  |  |  |  | NEG |  |  |  |  |  |
| Leucocyte 8-hydroxy-2-deoxygyanosine  (8-OHdG) | Chen*,* 2009 [4] | 211 |  |  |  |  | POS |  |  |  |  |  |
| Malondialdehyde (MDA) | Chen,, 2009 [4] | 211 |  |  |  |  | NEG |  |  |  |  |  |
|  | Sanyal*,* 2009 [11] | 80 | NSA |  |  |  | NSA |  |  |  |  |  |
|  | Molina*,* 1992 [12] | 37 | NSA | NSA | NSA |  | NSA |  |  |  |  |  |
|  | Ilic, 1999 [5] | 34 |  |  |  |  | NSA |  |  |  |  |  |

| **1.3 Free-radicals (continued)** | | | **Association of concentration of substance measured with:** | | | | | | | | | |
| --- | --- | --- | --- | --- | --- | --- | --- | --- | --- | --- | --- | --- |
| **Substance measured** | **Reference**  **(first author, year)** | **n** | **Total UPDRS** | **UPDRS**  **(III)** | **UPDRS**  **(II)** | **UPDRS**  **(I)** | **H&Y** | **MMSE** | **Blessed IMC** | **DRS** | **CURS** | **S&E** |
| Nitrate | Sanyal*,* 2010 [13] | 80 | r = 0.41 |  |  |  | r = 0.41 |  |  |  |  |  |
|  | Molina*,* 1994 [14] | 68 | NSA | NSA | NSA |  | NSA |  |  |  |  |  |
|  | Molina*,* 1996 [15] | 31 | NSA | NSA | NSA |  | NSA |  |  |  |  |  |
|  | Molina*,* 1996 [15] | 31 | NSA | NSA | NSA |  | NSA |  |  |  |  |  |
| CSF/Plasma Nitrate Ratio | Molina*,* 1996 [15] | 31 | NSA | NSA | NSA |  | NSA |  |  |  |  |  |
| Platelet Electron Transfer Complexes (ETC: I, III and IV) | Benecke*,* 1993 [16] | 27 |  |  |  |  | NSA (H&Y 2 to 4) |  |  |  |  |  |
| Platelet Monoamine oxidase-B (MAO-B) activity | Gotz*,* 2000 [17] | 58 |  |  |  |  | NSA |  |  |  |  |  |
|  | Husain*,* 2009 [18] | 26 |  |  |  |  | NSA |  |  |  |  |  |
|  | Bongioanni*,* 1996 [19] | 30 | r = 0.76 |  |  |  |  |  |  |  |  |  |
| Superoxide anion radical | Ilic*,* 1999 [5] | 34 |  |  |  |  | NSA |  |  |  |  |  |
| Superoxide dismutase (SOD) activity/SOD1 protein in RBC  (RBC-SOD/SOD1) | Ihara, 1999 [8] | 48 |  |  |  |  | No significant difference (H&Y 1-3 versus 4-5) |  |  |  |  |  |
| Superoxide dismutase (SOD) activity in plasma | Ihara, 1999 [8] | 48 |  |  |  |  | No significant difference (H&Y 1-3 versus 4-5) |  |  |  |  |  |
| Superoxide dismutase (SOD) 1 protein/total RBC protein (SOD1/RBC) | Ihara, 1999 [8] | 48 |  |  |  |  | No significant difference (H&Y 1-3 versus 4-5) |  |  |  |  |  |

| **1.3 Free-radicals (continued)** | |  | **Association of concentration of substance measured with:** | | | | | | | | | |
| --- | --- | --- | --- | --- | --- | --- | --- | --- | --- | --- | --- | --- |
| **Substance measured** | **Reference**  **(first author, year)** | **n** | **Total UPDRS** | **UPDRS**  **(III)** | **UPDRS**  **(II)** | **UPDRS**  **(I)** | **H&Y** | **MMSE** | **Blessed IMC** | **DRS** | **CURS** | **S&E** |
| Superoxide dismutase (SOD) 1 protein (SOD1/plasma) in plasma | Ihara, 1999 [8] | 48 |  |  |  |  | No significant difference (H&Y 1-3 versus 4-5) |  |  |  |  |  |
| Superoxide dismutase (SOD) 2 protein (SOD2/plasma) in plasma | Ihara, 1999 [8] | 48 |  |  |  |  | No significant difference (H&Y 1-3 versus 4-5) |  |  |  |  |  |
| Cu, and Zn-Superoxide dismutase activity (erythrocyte haemolysate) | Ilic, 1999 [5] | 34 |  |  |  |  | NSA |  |  |  |  |  |
| Whole Blood Monoamine oxidase-B (MAO-B) activity | Kuiper*,* 1993 [20] | 50 |  |  |  |  |  |  |  |  | NSA |  |

| **1.4 Catecholamines** | | | **Association of concentration of substance measured with:** | | | | | | | | | |
| --- | --- | --- | --- | --- | --- | --- | --- | --- | --- | --- | --- | --- |
| **Substance measured** | **Reference**  **(first author, year)** | **n** | **Total UPDRS** | **UPDRS**  **(III)** | **UPDRS**  **(II)** | **UPDRS**  **(I)** | **H&Y** | **MMSE** | **Blessed IMC** | **DRS** | **CURS** | **S&E** |
| Epinephrine | Eldrup, 1995 [3] | 16 |  |  |  |  | NSA |  |  |  |  |  |
| Noradrenaline | D'Andrea*,* 2010 [21] | 84 | Levels lower in UPDRS 18-36 than UPDRS ≤12* |  |  |  |  |  |  |  |  |  |
|  | Eldrup, 1995 [3] | 16 |  |  |  |  | NSA |  |  |  |  |  |
| Octopamine | D'Andrea*,* 2010 [21] | 84 | Levels lower in UPDRS ≤12 than UPDRS 18-36 and UPDRS >38^φ^ |  |  |  |  |  |  |  |  |  |

| **1.5 Amino acid and metabolites** | | | **Association of concentration of substance measured with:** | | | | | | | | | |
| --- | --- | --- | --- | --- | --- | --- | --- | --- | --- | --- | --- | --- |
| **Substance measured** | **Reference**  **(first author, year)** | **n** | **Total UPDRS** | **UPDRS**  **(III)** | **UPDRS**  **(II)** | **UPDRS**  **(I)** | **H&Y** | **MMSE** | **Blessed IMC** | **DRS** | **CURS** | **S&E** |
| Arginine | Molina, 1996 [15] | 31 | r = -0.42 | r = -0.54 |  |  | r = -0.42 |  |  |  |  |  |
| CSF/Plasma Arginine Ratio | Molina, 1996 [15] | 31 | NSA | NSA | NSA |  | NSA |  |  |  |  |  |
| Free carnitine | Jimenez-Jimenez*,* 1997 [22] | 29 | NSA | NSA | NSA |  | NSA |  |  |  |  |  |
| Acyl-carnitine esters | Jimenez-Jimenez*,* 1997 [22] | 29 | NSA | NSA | NSA |  | NSA |  |  |  |  |  |
| Citrulline | Molina, 1996 [15] | 31 | NSA | NSA | NSA |  | NSA |  |  |  |  |  |
| CSF/Plasma Citrulline Ratio | Molina, 1996 [15] | 31 | NSA | NSA | NSA |  | NSA |  |  |  |  |  |
| Glycine | Jimenez-Jimenez*,* 1996 [23] | 31 | r = -0.40 | r = -0.41 |  |  | r = -0.43 |  |  |  |  |  |
| Homocysteine | Camicioli*,* 2009 [24] | 47 |  | r = 0.11 |  |  |  |  |  | NSA |  |  |
|  | Hassin-Baer*,* 2006 [25] | 72 |  | NSA |  | NSA | NSA | NSA |  |  |  |  |
|  | O'Suilleabhain*,* 2004 [26] | 96 |  | NSA† | NSA† |  |  | NEG‡ |  |  |  |  |
|  | Rodriguez-Oroz*,* 2009 [27] | 89 |  |  |  |  |  | NSA |  |  |  |  |
|  | Irizarry*,* 2005 [28] | 90 |  |  |  |  | POS |  | r = 0.23 |  |  |  |
| Uric acid | Andreadou*,* 2009 [29] | 43 |  | NSA |  |  | Level in H&Y 2 higher than 2.5 or 3 |  |  |  |  |  |

| **1.6 Elements and transfer proteins** | | | **Association of concentration of substance measured with:** | | | | | | | | | | |
| --- | --- | --- | --- | --- | --- | --- | --- | --- | --- | --- | --- | --- | --- |
| **Substance measured** | **Reference**  **(first author, year)** | **n** | **Total UPDRS** | **UPDRS**  **(III)** | **UPDRS**  **(II)** | **UPDRS**  **(I)** | **H&Y** | | **MMSE** | **Blessed IMC** | **DRS** | **CURS** | **S&E** |
| Aluminium | Hegde*,* 2004 [30] | 52 |  |  |  |  | Level in H&Y ≥2 less than H&Y 1 | |  |  |  |  |  |
| Calcium | Hegde*,* 2004 [30] | 52 |  |  |  |  | Level in H&Y ≥2 less than H&Y 1 | |  |  |  |  |  |
| Copper | Arnal*,* 2010 [31] | 87 |  |  |  |  |  | | r = -0.95 |  |  |  |  |
|  | Hegde*,* 2004 [30] | 52 |  |  |  |  | Level in H&Y 1 higher than H&Y ≥ 2 | |  |  |  |  |  |
|  | Jimenez-Jimenez*,* 1992 [32] | 39 | NSA | NSA | NSA |  | NSA | |  |  |  |  |  |
|  | Jimenez-Jimenez*,* 1998 [33] | 37 | NSA | NSA | NSA |  | NSA | |  |  |  |  |  |
| Non-Ceruloplasmin bound Copper (NCBC) | Arnal, 2010 [31] | 87 |  |  |  |  |  | | r = -0.97 |  |  |  |  |
| Ceruloplasmin | Arnal, 2010 [31] | 87 |  |  |  |  |  | | r = -0.92 |  |  |  |  |
|  | Torsdottir*,* 2006 [34] | 28 |  |  |  |  | No significant difference (H&Y 2-3 versus 4-5) | |  |  |  |  |  |
|  | Jimenez-Jimenez*,* 1992 [32] | 39 | NSA | NSA | NSA |  | NSA | |  |  |  |  |  |
| Copper/Ceruloplasmin molar ratio | Jimenez-Jimenez*,* 1992 [32] | 39 | NSA | NSA | NSA |  | NSA | |  |  |  |  |  |
| Ceruloplasmin oxidative activity | Torsdottir*,* 2006 [34] | 28 |  |  |  |  | No significant difference (H&Y 2-3 versus 4-5) | |  |  |  |  |  |
| Ceruloplasmin specific oxidative activity | Torsdottir*,* 2006 [34] | 28 |  |  |  |  | No significant difference (H&Y 2-3 versus 4-5) | |  |  |  |  |  |
| **1.6 Elements and transfer proteins (continued)** | | | **Association of concentration of substance measured with:** | | | | | | | | | | |
| **Substance measured** | **Reference**  **(first author, year)** | **n** | **Total UPDRS** | **UPDRS**  **(III)** | **UPDRS**  **(II)** | **UPDRS**  **(I)** | **H&Y** | **MMSE** | | **Blessed IMC** | **DRS** | **CURS** | **S&E** |
| Metallothioneins (MTs) | Arnal, 2010 [31] | 87 |  |  |  |  |  | NSA | |  |  |  |  |
| Iron | Hegde, 2004 [30] | 52 |  |  |  |  | Level in H&Y ≥2 less than H&Y 1 |  | |  |  |  |  |
|  | Cabrera-Valdivia*,* 1994 [35] | 61 | NSA | NSA | NSA |  | NSA |  | |  |  |  |  |
|  | Jimenez-Jimenez, 1998 [33] | 37 | NSA | NSA | NSA |  | NSA |  | |  |  |  |  |
| Transferrin | Cabrera-Valdivia*,* 1994 [35] | 64 | NSA | NSA | NSA |  | NSA |  | |  |  |  |  |
| Ferritin | Cabrera-Valdivia*,* 1994 [35] | 62 | NSA | NSA | NSA |  | NSA |  | |  |  |  |  |
| Magnesium | Hegde, 2004 [30] | 52 |  |  |  |  | No significant difference (H&Y 1 versus ≥2) |  | |  |  |  |  |
| Manganese | Jimenez-Jimenez, 1998 [33] | 37 | NSA | NSA | NSA |  | NSA |  | |  |  |  |  |
| Phosphorus | Hegde, 2004 [30] | 52 |  |  |  |  | Level in H&Y ≥2 less than H&Y 1 |  | |  |  |  |  |
| Potassium | Hegde, 2004 [30] | 52 |  |  |  |  | Level in H&Y ≥2 less than H&Y 1 |  | |  |  |  |  |
| Selenium | Aguilar*,* 1998 [36] | 28 | NSA | NSA | NSA |  | NSA |  | |  |  |  |  |
| Sodium | Hegde, 2004 [30] | 52 |  |  |  |  | No significant difference (H&Y 1 versus ≥2) |  | |  |  |  |  |
| Sulphur | Hegde, 2004 [30] | 52 |  |  |  |  | Level in H&Y ≥2 less than H&Y 1 |  | |  |  |  |  |
| **1.6 Elements and transfer proteins (continued)** | | | **Association of concentration of substance measured with:** | | | | | | | | | | |
| **Substance measured** | **Reference**  **(first author, year)** | **n** | **Total UPDRS** | **UPDRS**  **(III)** | **UPDRS**  **(II)** | **UPDRS**  **(I)** | **H&Y** | **MMSE** | | **Blessed IMC** | **DRS** | **CURS** | **S&E** |
| Zinc | Hegde, 2004 [30] | 52 |  |  |  |  | Level in H&Y ≥2 less than H&Y 1 |  | |  |  |  |  |
|  | Jimenez-Jimenez, 1992 [32] | 32 | NSA | NSA | NSA |  | NSA |  | |  |  |  |  |
|  | Jimenez-Jimenez, 1998 [33] | 37 | NSA | NSA | NSA |  | NSA |  | |  |  |  |  |
| Albumin | Jimenez-Jimenez, 1992 [32] | 32 | NSA | NSA | NSA |  | NSA |  | |  |  |  |  |
| Zinc/albumin molar ratio | Jimenez-Jimenez, 1992 [32] | 32 | NSA | NSA | NSA |  | NSA |  | |  |  |  |  |

| **1.7 Vitamins and carrier proteins** | | | **Association of concentration of substance measured with:** | | | | | | | | | |
| --- | --- | --- | --- | --- | --- | --- | --- | --- | --- | --- | --- | --- |
| **Substance measured** | **Reference**  **(first author, year)** | **n** | **Total UPDRS** | **UPDRS**  **(III)** | **UPDRS**  **(II)** | **UPDRS**  **(I)** | **H&Y** | **MMSE** | **Blessed IMC** | **DRS** | **CURS** | **S&E** |
| Vitamin A | Jimenez-Jimenez, 1992 [37] | 42 | NSA | NSA | NSA |  | NSA |  |  |  |  |  |
|  | King*,* 1992 [38] | 27 |  |  |  |  | NSA |  |  |  |  |  |
| Vitamin A/Retinol-binding protein ratio | Jimenez-Jimenez, 1992 [37] | 42 | NSA | NSA | NSA |  | NSA |  |  |  |  |  |
| Retinol-binding protein (RBP) | Jimenez-Jimenez, 1992 [37] | 42 | NSA | NSA | NSA |  | NSA |  |  |  |  |  |
| Transthyretin | Jimenez-Jimenez, 1992 [37] | 42 | NSA | NSA | NSA |  | NSA |  |  |  |  |  |
| α-carotene | Jimenez-Jimenez, 1993 [39] | 61 | NSA | NSA | NSA |  | NSA |  |  |  |  |  |
| β-carotene | Jimenez-Jimenez, 1993 [39] | 61 | NSA | NSA | NSA |  | NSA |  |  |  |  |  |
| Lycopene | Jimenez-Jimenez, 1993 [39] | 61 | NSA | NSA | NSA |  | NSA |  |  |  |  |  |
| Retinol/β-carotene ratio | Jimenez-Jimenez, 1993 [39] | 61 | NSA | NSA | NSA |  | NSA |  |  |  |  |  |

| **1.7 Vitamins and carrier proteins (continued)** | | | **Association of concentration of substance measured with:** | | | | | | | | | |
| --- | --- | --- | --- | --- | --- | --- | --- | --- | --- | --- | --- | --- |
| **Substance measured** | **Reference**  **(first author, year)** | **n** | **Total UPDRS** | **UPDRS**  **(III)** | **UPDRS**  **(II)** | **UPDRS**  **(I)** | **H&Y** | **MMSE** | **Blessed IMC** | **DRS** | **CURS** | **S&E** |
| Vitamin B12 | Camicioli, 2009 [24] | 47 |  | NSA |  |  |  |  |  | NSA |  |  |
| Folate | Camicioli, 2009 [24] | 47 |  | NSA |  |  |  |  |  | NSA |  |  |
| Vitamin C (Serum) | Fernandez-Calle*,* 1993 [40] | 63 | NSA | NSA | NSA |  | NSA |  |  |  |  |  |
| Vitamin C (Leucocyte) | King, 1992 [38] | 27 |  |  |  |  | NSA |  |  |  |  |  |
| Vitamin E | Chen, 2009 [4] | 211 |  |  |  |  | NSA |  |  |  |  |  |
|  | Fernandez-Calle*,* 1992 [41] | 42 | NSA | NSA | NSA |  | NSA |  |  |  |  |  |
|  | King*,* 1992 [38] | 27 |  |  |  |  | NSA |  |  |  |  |  |
|  | Molina*,* 1997 [42] | 34 | NSA | NSA | NSA |  | NSA |  |  |  |  |  |
| Vitamin E/Cholesterol ratio | King*,* 1992 [38] | 27 |  |  |  |  | NSA |  |  |  |  |  |
| Coenzyme Q10 | Jimenez-Jimenez*,* 2000 [43] | 33 | NSA | NSA | NSA |  | NSA |  |  |  |  |  |
| Coenzyme Q10/cholesterol ratio | Jimenez-Jimenez*,* 2000 [43] | 33 | r = -0.39 | r = -0.45 |  |  |  |  |  |  |  |  |

| **1.8 Cytokines** | | | **Association of concentration of Substance measured with:** | | | | | | | | | |
| --- | --- | --- | --- | --- | --- | --- | --- | --- | --- | --- | --- | --- |
| **Substance measured** | **Reference**  **(first author, year)** | **n** | **Total UPDRS** | **UPDRS**  **(III)** | **UPDRS**  **(II)** | **UPDRS**  **(I)** | **H&Y** | **MMSE** | **Blessed IMC** | **DRS** | **CURS** | **S&E** |
| Interleukin 6 (IL-6) | Hofmann*,* 2009 [44] | ??º |  |  |  |  | NSA |  |  |  |  |  |
|  | Hofmann*,* 2009 [44] | 16º |  |  |  |  |  |  |  |  |  | r = -0.46 |
| Interleukin 10 (IL-10) | Rentzos*,* 2009 [45] | 41 |  | NSA |  |  | NSA |  |  |  |  |  |
| Interleukin 12 (IL-12) | Rentzos*,* 2009 [45] | 41 |  | NSA |  |  | NSA |  |  |  |  |  |
| Interleukin 15 (IL-15) | Rentzos*,* 2007 [46] | 41 |  |  |  |  | NSA |  |  |  |  |  |
| Regulated upon activation of normal T cell expressed and secreted (RANTES) | Rentzos*,* 2007 [46] | 41 |  | r = 0.42 |  |  | NSA |  |  |  |  |  |

| **1.9 Other** | | | **Association of concentration of Substance measured with:** | | | | | | | | | |
| --- | --- | --- | --- | --- | --- | --- | --- | --- | --- | --- | --- | --- |
| **Substance measured** | **Reference**  **(first author, year)** | **n** | **Total UPDRS** | **UPDRS**  **(III)** | **UPDRS**  **(II)** | **UPDRS**  **(I)** | **H&Y** | **MMSE** | **Blessed IMC** | **DRS** | **CURS** | **S&E** |
| Computed half-life of levodopa (t1/2_eq_) after a standardized oral levodopa test | Contin*,* 1998 [47] | 34 |  |  |  |  | r = -0.65 |  |  |  |  |  |
| Creatine kinase (CK) | Takubo*,* 2003 [48] | 84 |  |  |  |  | NSA |  |  |  |  |  |
| Cyclic guanosine 3'5'-monophosphate (cGMP) | Navarro*,* 1998 [49] | 22 | NSA | NSA | NSA |  | NSA |  |  |  |  |  |
| D3 dopamine receptor mRNA /β-actin mRNA (D3R/β-actin) expression in peripheral blood lymphocytes | Nagai*,* 1996 [50] | 45 |  |  |  |  | r = -0.69 |  |  |  |  |  |

| **1.9 Other (continued)** | | | **Association of concentration of substance measured with:** | | | | | | | | | |
| --- | --- | --- | --- | --- | --- | --- | --- | --- | --- | --- | --- | --- |
| **Substance measured** | **Reference**  **(first author, year)** | **n** | **Total UPDRS** | **UPDRS**  **(III)** | **UPDRS**  **(II)** | **UPDRS**  **(I)** | **H&Y** | **MMSE** | **Blessed IMC** | **DRS** | **CURS** | **S&E** |
| Neuron-specific enolase (NSE) | Schaf*,* 2005 [51] | 40 |  |  |  |  | NSA |  |  |  |  | NSA |
| Platelet Count | Gotz, 2000 [17] | 58 |  |  |  |  | NSA |  |  |  |  |  |
| Platelet Protein | Gotz, 2000 [17] | 58 |  |  |  |  | NSA |  |  |  |  |  |
| S100B | Schaf*,* 2005 [51] | 40 |  |  |  |  | r = 0.37 |  |  |  |  | r = -0.43 |

Values for serum and plasma are grouped together for simplicity.

**Key**

† This study calculated and combined z scores for UPDRS (II) and UPDRS (III) as a measure of physical state. Physical state was not significantly worse in those with higher homocysteine levels.

‡ This study calculated and combined z scores for 11 tests of cognition, including MMSE, as a measure of global cognition. Cognitive outcome was significantly worse in those with higher homocysteine levels.

* Patients were split into three groups:

(1) De novo - non-fluctuating, symptoms less than one year, not on treatment, UPDRS ≤12;

(2) Non-fluctuating - UPDRS 18-36, treated with levodopa and carbidopa (therapy effective);

(3) Fluctuating - UPDRS > 38, treated with levodopa and a dopamine agonists with incomplete response to therapy.

Noradrenaline plasma levels were significantly (P=0.02) reduced in the non-fluctuating group compared to the de novo group.

^φ^ Patients were split into three groups:

(1) De novo - non-fluctuating, symptoms less than one year, not on treatment, UPDRS ≤12;

(2) Non-fluctuating - UPDRS 18-36, treated with levodopa and carbidopa (therapy effective);

(3) Fluctuating - UPDRS > 38, treated with levodopa and a dopamine agonists with incomplete response to therapy.

Octopamine plasma levels were significantly lower in the de novo patient group than the non-fluctuating (P <0.001) and fluctuating groups (P <0.001).

º The Association between IL-6 and Schwab and England was examined using data from patients treated with levodopa (n=23). However, it is unclear from the text how many patients were used to undertake the analysis with regards to Hoehn and Yahr stage.

**Additional file 3 *2: Cerebrospinal fluid (CSF)***

**Associations between putative cerebrospinal fluid (CSF) biomarkers and clinical measures of disease severity, in**

**cross-sectional studies included in the systemic review of biomarkers for disease progression in Parkinson’s disease**

| **2.1 Structural proteins** | | | **Association of concentration of substance measured with:** | | | | | | | | | |
| --- | --- | --- | --- | --- | --- | --- | --- | --- | --- | --- | --- | --- |
| **Substance measured** | **Reference**  **(first author, year)** | **n** | **Total UPDRS** | **UPDRS**  **(III)** | **UPDRS**  **(II)** | **H&Y** | **CURS** | **MMSE** | **GDS** | **HDSR** | **WDS** | **NUDS** |
| α-synuclein | Hong*,* 2010 [52] | 117 |  |  |  | NSA |  |  |  |  |  |  |
| β-amyloid | Compta*,* 2009 [53] | 40 |  | NSA |  | NSA |  |  |  |  |  |  |
|  | Mollenhauer*,* 2006 [54] | 73* |  | NSA |  | NSA |  | NSA |  |  |  |  |
|  | Mollenhauer*,* 2006 [54] | 41* |  | NSA |  | NSA |  | NSA |  |  |  |  |
| DJ-1 | Hong*,* 2010 [52] | 117 |  |  |  | NSA |  |  |  |  |  |  |
|  | Waragai*,* 2006 [55] | 40 |  |  |  | Level in H&Y 1-2 higher than 3-4 |  |  |  |  |  |  |
| Neural thread protein | Yamada*,* 1993 [56] | 11 |  |  |  | NSA |  | r = 0.96^φ^ |  |  |  |  |
| Phospho-Tau | Compta*,* 2009 [53] | 40 |  | NSA |  | NSA |  |  |  |  |  |  |
| Tau | Compta*,* 2009 [53] | 40 |  | NSA |  | NSA |  |  |  |  |  |  |
|  | Mollenhauer*,* 2006 [54] | 73* |  | NSA |  | NSA |  | NSA |  |  |  |  |
|  | Mollenhauer*,* 2006 [54] | 41* |  | NSA |  | NSA |  | NSA |  |  |  |  |
|  | Molina*,* 1997 [57] | 26 | NSA | NSA | NSA | NSA |  |  |  |  |  |  |

| **2.2 Neurotransmitters** | | | **Association of concentration of substance measured with:** | | | | | | | | | |
| --- | --- | --- | --- | --- | --- | --- | --- | --- | --- | --- | --- | --- |
| **Substance measured** | **Reference**  **(first author, year)** | **n** | **Total UPDRS** | **UPDRS**  **(III)** | **UPDRS**  **(II)** | **H&Y** | **CURS** | **MMSE** | **GDS** | **HDSR** | **WDS** | **NUDS** |
| Acetylcholinesterase activity | Konings*,* 1995 [58] | 85‡ |  |  |  |  | NSA |  |  |  |  |  |
|  | Konings*,* 1995 [58] | 18‡ |  |  |  |  | NSA |  |  |  |  |  |
| Acetylcholinesterase immunoreactivity | Konings*,* 1995 [58] | 85‡ |  |  |  |  | NSA |  |  |  |  |  |
|  | Konings*,* 1995 [58] | 18‡ |  |  |  |  | NSA |  |  |  |  |  |
| Acetylcholinesterase homospecific activity | Konings*,* 1995 [58] | 85‡ |  |  |  |  | NSA |  |  |  |  |  |
|  | Konings*,* 1995 [58] | 18‡ |  |  |  |  | NSA |  |  |  |  |  |
| Dihydroxyphenylacetic acid (DOPAC) | Lunardi, 2009 [59] | 45 | r = -0.42 |  |  | r = -0.47 |  |  |  |  |  |  |
|  | Eldrup, 1995 [3] | 10† |  |  |  | r = -0.71 |  |  |  |  |  |  |
|  | Eldrup, 1995 [3] | 6† |  |  |  | NSA |  |  |  |  |  |  |
| 3,4-dihydroxyphenylalanine (DOPA) | Eldrup, 1995 [3] | 10† |  |  |  | NSA |  |  |  |  |  |  |
|  | Eldrup, 1995 [3] | 6† |  |  |  | NSA |  |  |  |  |  |  |
| Dopamine | Lunardi*,* 2009 [59] | 45 | r = -0.45 |  |  | r = -0.47 |  |  |  |  |  |  |
|  | Eldrup, 1995 [3] | 10† |  |  |  | NSA |  |  |  |  |  |  |
|  | Eldrup, 1995 [3] | 6† |  |  |  | NSA |  |  |  |  |  |  |
|  | Tohgi*,* 1990 [60] | 21 |  |  |  | NEG |  |  |  |  |  |  |
|  | Tohgi*,* 1993 [61] | 39 |  |  |  | NEG |  |  |  |  |  |  |
|  | Tohgi*,* 1997 [62] | 16 |  |  |  | r = -0.53 |  |  |  | NSA |  |  |
| 5-hydroxyindoleacetic acid (5-HIAA) | Strittmatter*,* 1996 [63] | 35 |  |  |  | NSA |  | NSA | NSA |  | NSA |  |
|  | Tohgi, 1993 [61] | 39 |  |  |  | Level in H&Y 2 higher than 1 |  |  |  |  |  |  |
| 5-hydroxytryptamine  (5-HT) | Tohgi, 1997 [62] | 16 |  |  |  | r = -0.74 |  |  |  | NSA |  |  |

| **2.3 Free-radicals** | | | **Association of concentration of substance measured with:** | | | | | | | | | |
| --- | --- | --- | --- | --- | --- | --- | --- | --- | --- | --- | --- | --- |
| **Substance measured** | **Reference**  **(first author, year)** | **n** | **Total UPDRS** | **UPDRS**  **(III)** | **UPDRS**  **(II)** | **H&Y** | **CURS** | **MMSE** | **GDS** | **HDSR** | **WDS** | **NUDS** |
| Homovanillic acid | Lunardi, 2009 [59] | 45 | NSA |  |  | r = -0.36 |  |  |  |  |  |  |
|  | Strittmatter*,* 1996 [63] | 35 |  |  |  | NSA |  | NSA | NSA |  | NSA |  |
|  | Tohgi, 1993 [61] | 39 |  |  |  | NEG |  |  |  |  |  |  |
| Malondialdehyde (MDA) | Shukla, 2006 [64] | 21 |  | NSA |  | NSA |  |  |  |  |  |  |
|  | Ilic, 1999 [5] | 34 |  |  |  | NSA |  |  |  |  |  |  |
| Nitrates | Molina,,1996 [15] | 31 | NSA | NSA | NSA | NSA |  |  |  |  |  |  |
| CSF/Plasma nitrate ratio | Molina,,1996 [15] | 31 | NSA | NSA | NSA | NSA |  |  |  |  |  |  |
| Nitrite | Shukla*,* 2006 [64] | 21 |  | NSA |  | NSA |  |  |  |  |  |  |

| **2.4 Catecholamines** | | | **Association of concentration of substance measured with:** | | | | | | | | | |
| --- | --- | --- | --- | --- | --- | --- | --- | --- | --- | --- | --- | --- |
| **Substance measured** | **Reference**  **(first author, year)** | **n** | **Total UPDRS** | **UPDRS**  **(III)** | **UPDRS**  **(II)** | **H&Y** | **CURS** | **MMSE** | **GDS** | **HDSR** | **WDS** | **NUDS** |
| Epinephrine | Eldrup, 1995 [3] | 10† |  |  |  | NSA |  |  |  |  |  |  |
|  | Eldrup, 1995 [3] | 6† |  |  |  | NSA |  |  |  |  |  |  |
|  | Tohgi, 1990 [60] | 21 |  |  |  | NSA |  |  |  |  |  |  |
| 3-methoxytyramine-4-hydroxyphenylglycol (MHPG) | Tohgi, 1993 [61] | 39 |  |  |  | Level higher in H&Y 2 than 1 |  |  |  |  |  |  |
| Noradrenaline | Eldrup, 1995 [3] | 10† |  |  |  | NSA |  |  |  |  |  |  |
|  | Eldrup, 1995 [3] | 6† |  |  |  | NSA |  |  |  |  |  |  |
|  | Tohgi, 1990 [60] | 21 |  |  |  | NSA |  |  |  |  |  |  |
|  | Tohgi, 1993 [61] | 39 |  |  |  | NEG |  |  |  |  |  |  |
|  | Tohgi, 1997 [62] | 16 |  |  |  | r = -0.68 |  |  |  | NSA |  |  |

| **2.5 Amino acids and metabolites** | | | **Association of concentration of substance measured with:** | | | | | | | | | |
| --- | --- | --- | --- | --- | --- | --- | --- | --- | --- | --- | --- | --- |
| **Substance measured** | **Reference**  **(first author, year)** | **n** | **Total UPDRS** | **UPDRS**  **(III)** | **UPDRS**  **(II)** | **H&Y** | **CURS** | **MMSE** | **GDS** | **HDSR** | **WDS** | **NUDS** |
| Arginine | Molina, 1996 [15] | 31 | NSA | NSA | NSA | NSA |  |  |  |  |  |  |
| CSF/Plasma Arginine ratio | Molina, 1996 [15] | 31 | NSA | NSA | NSA | NSA |  |  |  |  |  |  |
| Asparagine | Jimenez-Jimenez, 1996 [23] | 31 | NSA | NSA | NSA | NSA |  |  |  |  |  |  |
| Aspartate | Jimenez-Jimenez, 1996 [23] | 31 | NSA | NSA | NSA | NSA |  |  |  |  |  |  |
| Free Carnitine | Jimenez-Jimenez, 1997 [22] | 29 | NSA | NSA | NSA | r = -0.41 |  |  |  |  |  |  |
| Acyl-carnitine esters | Jimenez-Jimenez, 1997 [22] | 29 | NSA | NSA | NSA | NSA |  |  |  |  |  |  |
| Citrulline | Molina, 1996 [15] | 31 | NSA | NSA | NSA | NSA |  |  |  |  |  |  |
| CSF/Plasma Citrulline ratio | Molina, 1996 [15] | 31 | NSA | NSA | NSA | NSA |  |  |  |  |  |  |
| Gamma-aminobutyric acid (GABA) | Jimenez-Jimenez, 1996 [23] | 31 | NSA | NSA | NSA | NSA |  |  |  |  |  |  |
| Glutamate | Jimenez-Jimenez, 1996 [23] | 31 | NSA | NSA | NSA | NSA |  |  |  |  |  |  |
| Glutamine | Jimenez-Jimenez, 1996 [23] | 31 | NSA | NSA | NSA | NSA |  |  |  |  |  |  |
| Glycine | Jimenez-Jimenez, 1996 [23] | 31 | NSA | NSA | NSA | NSA |  |  |  |  |  |  |

| **2.6 Elements and transfer proteins** | | | **Association of concentration of substance measured with:** | | | | | | | | | |
| --- | --- | --- | --- | --- | --- | --- | --- | --- | --- | --- | --- | --- |
| **Substance measured** | **Reference**  **(first author, year)** | **n** | **Total UPDRS** | **UPDRS**  **(III)** | **UPDRS**  **(II)** | **H&Y** | **CURS** | **MMSE** | **GDS** | **HDSR** | **WDS** | **NUDS** |
| Copper | Jimenez-Jimenez, 1998 [33] | 37 | NSA | NSA | NSA | NSA |  |  |  |  |  |  |
| Phenanthroline-copper | Pall*,* 1987 [65] | 28 |  |  |  |  |  |  |  |  | r =0.47 | POS |
| Iron | Jimenez-Jimenez, 1998 [33] | 37 | NSA | NSA | NSA | NSA |  |  |  |  |  |  |
| Manganese | Jimenez-Jimenez, 1998 [33] | 37 | NSA | NSA | NSA | NSA |  |  |  |  |  |  |
| Selenium | Aguilar, 1998 [36] | 28 | NSA | NSA | NSA | NSA |  |  |  |  |  |  |
| Zinc | Jimenez-Jimenez, 1998 [33] | 37 | NSA | NSA | NSA | NSA |  |  |  |  |  |  |

| **2.7 Vitamins and carrier proteins** | | | **Association of concentration of substance measured with:** | | | | | | | | | |
| --- | --- | --- | --- | --- | --- | --- | --- | --- | --- | --- | --- | --- |
| **Substance measured** | **Reference**  **(first author, year)** | **n** | **Total UPDRS** | **UPDRS**  **(III)** | **UPDRS**  **(II)** | **H&Y** | **CURS** | **MMSE** | **GDS** | **HDSR** | **WDS** | **NUDS** |
| Total thiamine | Jimenez-Jimenez*,* 1999 [66] | 24 | NSA | NSA | NSA | NSA |  |  |  |  |  |  |
| Free thiamine | Jimenez-Jimenez*,* 1999 [66] | 24 | NSA | NSA | NSA | NSA |  |  |  |  |  |  |
| Thiamine diphosphate | Jimenez-Jimenez*,* 1999 [66] | 24 | NSA | NSA | NSA | NSA |  |  |  |  |  |  |
| Thiamine monophosphate | Jimenez-Jimenez*,* 1999 [66] | 24 | NSA | NSA | NSA | NSA |  |  |  |  |  |  |
| Vitamin E | Molina, 1997 [42] | 34 | NSA | NSA | NSA | NSA |  |  |  |  |  |  |

| **2.8 Cytokines** | | | **Association of concentration of substance measured with:** | | | | | | | | | |
| --- | --- | --- | --- | --- | --- | --- | --- | --- | --- | --- | --- | --- |
| **Substance measured** | **Reference**  **(first author, year)** | **n** | **Total UPDRS** | **UPDRS**  **(III)** | **UPDRS**  **(II)** | **H&Y** | **CURS** | **MMSE** | **GDS** | **HDSR** | **WDS** | **NUDS** |
| Interleukin 6 (IL-6) | Muller*,* 1998 [67] | 12 | r = -0.65 | r = -0.65 | r =-0.69 |  |  |  |  |  |  |  |

| **2.9 Other** | | | **Association of concentration of substance measured with:** | | | | | | | | | |
| --- | --- | --- | --- | --- | --- | --- | --- | --- | --- | --- | --- | --- |
| **Substance measured** | **Reference**  **(first author, year)** | **n** | **Total UPDRS** | **UPDRS**  **(III)** | **UPDRS**  **(II)** | **H&Y** | **CURS** | **MMSE** | **GDS** | **HDSR** | **WDS** | **NUDS** |
| Alpha-melanocyte stimulating hormone-like immunoreactivity (α-MSH-LI) | Rainero*,* 1988 [68] | 9 |  |  |  |  | NSA |  |  |  |  |  |
| Angiotensin converting enzyme (ACE) | Konings*,* 1994 [69] | 88˚ |  |  |  |  | NSA |  |  |  |  |  |
|  | Konings*,* 1994 [69] | 18˚ |  |  |  |  | NSA |  |  |  |  |  |
| Cyclic guanosine 3'5'-monophosphate(cGMP) | Navarro,1998 [49] | 22 | NSA | NSA | NSA | NSA |  |  |  |  |  |  |
| Insulin | Jimenez-Jimenez*,* 2000 [70] | 24 | NSA | NSA | NSA | NSA |  |  |  |  |  |  |
| Somatostatin-like immunoreactivity (SLI) | Strittmatter, 1996 [63] | 35 |  |  |  |  |  |  | NSA |  |  |  |
| Tissue transglutaminase (tTG) | Vermes*,* 2004 [71] | 54 |  |  |  |  |  | NSA |  |  |  |  |

**Key**

* Examined for correlations in patients with Parkinson’s disease with dementia (n=73) and Parkinson’s disease without dementia (n=41) separately

† Examined for correlations in patients on anti-parkinsonian treatment (n=10) and those untreated (n=6) separately

‡ Examined for correlations in patients with Parkinson’s disease with dementia (n=18) and Parkinson’s disease without dementia (n=85) separately

˚ Examined for correlations in patients with Parkinson’s disease with dementia (n=18) and Parkinson’s disease without dementia (n=88) separately

^φ^ Examined for correlation to MMSE based on grouping patients into MMSE categories: (1) Non-demented, MMSE >23; (2) Mild dementia, MMSE 23-15; (3) Moderate dementia, MMSE 14-5; (4) severe dementia, MMSE <5.

**Additional file 3 *3: Urine***

**Associations between putative urine biomarkers and clinical measures of disease severity, in cross-sectional studies included in the systemic review of biomarkers for disease progression in Parkinson’s disease**

|  | | | **Association of concentration of substance measured with:** | | | | |
| --- | --- | --- | --- | --- | --- | --- | --- |
| **Substance measured** | **Reference**  **(first author, year)** | **n** | **Total UPDRS** | **UPDRS**  **(III)** | **UPDRS**  **(II)** | **H&Y** | **MMSE** |
| 8-hydroxy-2-deoxygyanosine (8-OHdG) | Seet, 2010 [6] | 61 |  |  |  | NEG |  |
| 8-hydroxy-2-deoxygyanosine/creatinine ratio (8-OHdG/creatinine ratio) | Sato*,* 2005 [72] | 72 |  |  |  | Ratio increases as H&Y increases. (Several comparisons: H&Y 1 v 2, 2 v 4, 4 v 5) |  |
| 8,12-isoprostane F_2α_-VI (iP) | Connolly, 2008 [10] | 26 |  | NSA | NSA |  | NSA |
| N-methyl-norsalsolinol | Scholz*,* 2004 [73] | 47 |  | NSA |  |  | NSA |
| Norsalsolinol | Scholz*,* 2004 [73] | 47 |  | NSA |  |  | NSA |
| Salsolinol | Scholz*,* 2004 [73] | 47 |  | NSA |  |  | NSA |
| 24 hour urinary iron excretion following a single dose of desferrioxamine | Cabrera-Valdivia, 1994 [35] | 68 | NSA | NSA | NSA | NSA |  |

**Additional file 3 *4: Cranial ultrasound***

**Associations between putative cranial ultrasound biomarkers and clinical measures of disease severity, in**

**cross-sectional studies included in the systemic review of biomarkers for disease progression in Parkinson’s disease**

|  |  |  | **Association of ultrasound feature measured with:** | | | | | |
| --- | --- | --- | --- | --- | --- | --- | --- | --- |
| **Ultrasound feature measured** | **Reference**  **(first author, year)** | **n** | **Total UPDRS** | **UPDRS (III)** | **Contralateral UPDRS (III)** | **UPDRS**  **(I)** | **H&Y** | **CURS** |
| Mean area of bilateral substantia nigra hyperechogenicity | Kim*,* 2007 [74] | 35 |  | NSA |  |  | NSA |  |
|  | Weise*,* 2009 [75] | 50 |  |  |  |  | r = 0.28 |  |
| Unilateral area of substantia nigra hyperechogenicity | Kim*,* 2007 [74] | 35 |  |  | NSA |  |  |  |
|  | Berg*,* 2005 [76] | 27* | r = 0.25 | r = 0.31 |  |  |  |  |
|  | Berg*,* 2005 [76] | ?* | r = 0.33 | r = 0.34 |  |  |  |  |
|  | Berg*,* 2001 [77] | 103 |  |  |  |  | No significant difference in H&Y between 2 groups† | No significant difference in CURS between 2 groups† |
| Larger unilateral area of substantia nigra hyperechogenicity | Walter*,* 2007 [78] | 97 |  | NSA |  |  |  |  |
| Ratio of mean area of bilateral substantia nigra hyperechogenicity to midbrain area | Kim*,* 2007 [74] | 35 |  | NSA |  |  | NSA |  |
| Ratio of unilateral area of substantia nigra hyperechogenicity to midbrain area | Kim*,* 2007 [74] | 35 |  |  | NSA |  |  |  |

|  |  |  | **Association of ultrasound feature measured with:** | | | | | |
| --- | --- | --- | --- | --- | --- | --- | --- | --- |
| **Ultrasound feature measured** | **Reference**  **(first author, year)** | **n** | **Total UPDRS** | **UPDRS (III)** | **Contralateral UPDRS (III)** | **UPDRS**  **(I)** | **H&Y** | **CURS** |
| Brainstem raphe (BR) echogenicity | Walter*,* 2007 [78] | 97 |  | No significant difference in UPDRS (III) between those with reduced (n=24) and normal (n=73) BR echogenicity |  |  |  |  |
| Caudate nucleus (CN) hyperechogenicity | Walter*,* 2007 [78] | 89 |  | UPDRS (III) higher in those with CN hyperechogenicity (n=45) than without (n=44). |  |  |  |  |
| Lenticular nucleus (LN) hyperechogenicity | Walter*,* 2007 [78] | 88 |  | No significant difference in UPDRS (III) between those with LN hyperechogenicity (n=19) and without (n=69) |  |  |  |  |
| Mean width of third ventricle | Walter*,* 2007 [78] | ?‡ |  | r = 0.28 |  |  |  |  |
| Mean width of frontal horns of lateral ventricles | Walter*,* 2007 [78] | ?‡ |  | r = 0.22 |  |  |  |  |
| Common carotid artery intima-media thickness (IMT) | Hassin-Baer, 2006 [25] | 45 |  | NSA |  | NSA | NSA |  |
| Flow volume:  Net for vertebral arteries | Haktanir*,* 2006 [79] | 28 |  |  |  |  | No difference between stages |  |
| Net for internal carotid arteries | Haktanir*,* 2006 [79] | 28 |  |  |  |  | No difference between stages |  |

|  |  |  | **Association of ultrasound feature measured with:** | | | | | |
| --- | --- | --- | --- | --- | --- | --- | --- | --- |
| **Ultrasound feature measured** | **Reference**  **(first author, year)** | **n** | **Total UPDRS** | **UPDRS (III)** | **Contralateral UPDRS (III)** | **UPDRS**  **(I)** | **H&Y** | **CURS** |
| Peak systolic velocity:  Left vertebral artery | Haktanir*,* 2006 [79] | 28 |  |  |  |  | No difference between stages |  |
| Right vertebral artery | Haktanir*,* 2006 [79] | 28 |  |  |  |  | No difference between stages |  |
| Left internal carotid artery | Haktanir*,* 2006 [79] | 28 |  |  |  |  | No difference between stages |  |
| Right internal carotid artery | Haktanir*,* 2006 [79] | 28 |  |  |  |  | No difference between stages |  |
| Cross-sectional area:  Left vertebral artery | Haktanir*,* 2006 [79] | 28 |  |  |  |  | No difference between stages |  |
| Right vertebral artery | Haktanir*,* 2006 [79] | 28 |  |  |  |  | No difference between stages |  |
| Left internal carotid artery | Haktanir*,* 2006 [79] | 28 |  |  |  |  | No difference between stages |  |
| Right internal carotid artery | Haktanir*,* 2006 [79] | 28 |  |  |  |  | No difference between stages |  |
| Cerebral blood flow | Haktanir*,* 2006 [79] | 28 |  |  |  |  | No difference between stages |  |

**Key**

* Longitudinal study (measurement at baseline and 5 years), but no longitudinal correlations. Not clear how many of the original 27 were rescanned five years later. First entry is data and correlations from baseline, second entry from year five. Comparing baseline data with data five years later there was no significant difference in size of SN hyperechogenicity either for the right side (P = 0.98) or the left side (P = 0.53).

† For analysis PD patients split into two groups: (1) area of substantia nigra hyperechogenicity ≤0.19cm^2^ on both sides (n=9); (2) area of substantia nigra hyperechogenicity >0.19cm^2^ on one or both sides (n=94). This value was chosen as it represented the upper standard deviation of the control group.

‡ Whilst paper states the number of patients who were accessible for measuring other parameters, it does not for ventricular widths.

**Additional file 3 *5: Cardiac ^123^I-MIBG scintigraphy***

**Associations between putative cardiac ^123^I-MIBG scintigraphy biomarkers and clinical measures of disease severity, in cross-sectional studies included in the systemic review of biomarkers for disease progression in Parkinson’s disease**

|  | |  |  | **Association of ^123^I-MIBG myocardial scintigraphy measurement with:** | | |
| --- | --- | --- | --- | --- | --- | --- |
| **^123^I-MIBG myocardial scintigraphy measurement** | | **Reference**  **(first author, year)** | **n** | **Total UPDRS** | **UPDRS**  **(III)** | **H&Y** |
| Early H/M ratio of ^123^I-MIBG uptake: | |  |  |  |  |  |
|  | 15 minutes | Hamada*,* 2003 [80] | 88 |  |  | r = -0.30 |
|  | 15 minutes | Matsui*,* 2006 [81] | 40 |  | NSA | NSA |
|  | 15 minutes | Shindo*,* 2005 [82] | 14 |  |  | NSA |
|  | 20 minutes | Chun*,* 2009 [83] | 27 |  |  | NSA |
|  | 20 minutes | Orimo*,* 1999 [84] | 45 | r = -0.37 |  | Ratio in H&Y 1 significantly higher than in H&Y 3, 4 and 5 |
|  | 20 minutes | Saiki*,* 2004 [85] | 34 | r = - 0.004 |  | Ratio in H&Y 1 higher than in H&Y 2, 3 and 4 but these differences were not statistically significant |
| Delayed H/M ratio of ^123^I-MIBG uptake: | |  |  |  |  |  |
|  | 3 hours | Matsui*,* 2006 [81] | 40 |  | NSA | NSA |
|  | 3 hours | Shindo*,* 2005 [82] | 14 |  |  | NSA |
|  | 3 hours | Orimo*,* 1999 [84] | 45 | r = -0.35 |  | Ratio in H&Y 1 significantly higher than in H&Y 3, 4 and 5. |
|  | 3 hours | Saiki*,* 2004 [85] | 34 | NSA |  | Ratio in H&Y 1 higher than in H&Y 2, 3 and 4 but these differences were not statistically significant |
|  | 3.5 hours | Spiegel*,* 2005 [86] | 18 |  | NSA |  |
|  | 3 or 4 hours | Hamada*,* 2003 [80] | 88 |  |  | r = -0.32 |
|  | 4 hours | Nagayama*,* 2005 [87] | 122 |  |  | NEG |
|  | 4 hours | Chun*,* 2009 [83] | 27 |  |  | NSA |
|  | 4 hours | Satoh*,* 1999 [88] | 35 |  |  | Ratio in H&Y 2 significant higher than in H&Y 3, 4 and 5. No significant difference in H/M ratio was found in H&Y 3, 4 and 5. |
| MIBG Washout rate (WR) | | Chun*,* 2009 [83] | 27 |  |  | NSA |
|  | | Shindo*,* 2005 [82] | 14 |  |  | NSA |

**Key**

123I-MIBG ^123^I-metaiodobenzylguanidine H/M Heart to mediastinal ratio

**Additional file 3 *6: Brain MRI and brain MRS***

**Associations between putative brain MRI and brain MRS biomarkers and clinical measures of disease severity, in cross-sectional studies included in the systemic review of biomarkers for disease progression in Parkinson’s disease**

| **6.1 MRI - Substantia nigra (SN)** | | | | **Association of measurement with:** | | | | | | | | | | |
| --- | --- | --- | --- | --- | --- | --- | --- | --- | --- | --- | --- | --- | --- | --- |
| **MRI modality** | **Measurement** | **Reference**  **(first author, year)** | **n** | **Total UPDRS** | **UPDRS**  **(III)** | **Lateralized UPDRS (III) from side of body affected:** | | | **UPDRS**  **(II)** | **UPDRS**  **(I)** | **H&Y** | **MMSE** | **S&E** | **TCS** |
|  |  |  |  |  |  | **Most** | **Least** | **Either** |  |  |  |  |  |  |
| T2-weighted MRI | Width of SN pars compacta | Duguid*,* 1986 [89] | 6 |  |  |  |  |  |  |  | NSA |  |  |  |
| Whole brain T2-weighted 2D fast spin echo MRI | Mean width of SN pars compacta | Atasoy*,* 2004 [90] | 20 | NSA | NSA |  |  |  | NSA | NSA |  |  |  |  |
|  | Mean intensity score of SN pars compacta | Atasoy*,* 2004 [90] | 20 | r = 0.63 | NSA |  |  |  | NSA | NSA |  |  |  |  |
| DTI MRI | SN fractional anisotropy (FA) value | Chan*,* 2007 [91] | 73 |  |  |  |  |  |  |  | r = -0.02 |  |  |  |
|  | SN apparent diffusion coefficient (ADC) | Chan*,* 2007 [91] | 73 |  |  |  |  |  |  |  | NSA |  |  |  |
| Multiple gradient echo sequence MRI | Contralateral lateral SN pars compacta R_2_* | Martin*,* 2008 [92] | 22 | NSA |  | r = 0.52 |  |  |  |  |  |  |  |  |
|  | Contralateral medial SN pars compacta R_2_* | Martin*,* 2008 [92] | 22 | NSA |  | NSA |  |  |  |  |  |  |  |  |

| **6.1 MRI - Substantia nigra (SN) (continued)** | | | | **Association of measurement with:** | | | | | | | | | | |
| --- | --- | --- | --- | --- | --- | --- | --- | --- | --- | --- | --- | --- | --- | --- |
| **MRI modality** | **Measurement** | **Reference**  **(first author, year)** | **n** | **Total UPDRS** | **UPDRS**  **(III)** | **Lateralized UPDRS (III) from side of body affected:** | | | **UPDRS**  **(II)** | **UPDRS**  **(I)** | **H&Y** | **MMSE** | **S&E** | **TCS** |
|  |  |  |  |  |  | **Most** | **Least** | **Either** |  |  |  |  |  |  |
| Multiple gradient echo sequence MRI | Contralateral lateral SN reticulata R_2_* | Martin*,* 2008 [92] | 22 | NSA |  | NSA |  |  |  |  |  |  |  |  |
|  | Contralateral medial SN reticulata R_2_* | Martin*,* 2008 [92] | 22 | NSA |  | NSA |  |  |  |  |  |  |  |  |
| MRI with 3-tesla system | Mean SN R_2_ | Gorell*,* 1995 [93] | 8 |  |  |  |  | NSA |  |  | NSA |  |  |  |
|  | Mean SN R_2_* | Gorell*,* 1995 [93] | 8 |  |  |  |  | NSA |  |  | NSA |  |  |  |
|  | Mean SN R_2_ prime | Gorell*,* 1995 [93] | 8 |  |  |  |  | NSA |  |  | NSA |  |  |  |
|  | Contralateral SN R_2_ | Gorell*,* 1995 [93] | 8 |  |  |  |  | NSA |  |  |  |  |  |  |
|  | Contralateral SN R_2_* | Gorell*,* 1995 [93] | 8 |  |  |  |  | NSA |  |  |  |  |  |  |
|  | Contralateral SN R_2_ prime | Gorell*,* 1995 [93] | 8 |  |  |  |  | NSA |  |  |  |  |  |  |
| PRIME sequence MRI | Contralateral SN R_2_' relaxation rate | Wallis*,* 2008 [94] | 65 |  |  | r = 0.29 | NSA |  |  |  |  |  |  |  |
|  | Mean SN R_2_ | Graham*,* 2000 [95] | 21 |  | NSA |  |  |  | NSA |  |  |  |  |  |
|  | Mean SN R_2_* | Graham*,* 2000 [95] | 21 |  | NSA |  |  |  | NSA |  |  |  |  |  |
|  | Mean SN R_2_' | Graham*,* 2000 [95] | 21 |  | NSA |  |  |  | NSA |  |  |  |  |  |
| SWI MRI | Contralateral phase shift values of SN | Zhang*,* 2010 [96] | 40 |  |  | r = 0.41 | r = 0.36 |  |  |  |  |  |  |  |
| **6.1 MRI - Substantia nigra (SN) (continued)** | | | | **Association of measurement with:** | | | | | | | | | | |
| **MRI modality** | **Measurement** | **Reference**  **(first author, year)** | **n** | **Total UPDRS** | **UPDRS**  **(III)** | **Lateralized UPDRS (III) from side of body affected:** | | | **UPDRS**  **(II)** | **UPDRS**  **(I)** | **H&Y** | **MMSE** | **S&E** | **TCS** |
|  |  |  |  |  |  | **Most** | **Least** | **Either** |  |  |  |  |  |  |
| T2-weighted MRI | Bilateral SN T2 value | Antonini*,* 1993 [97] | 30 |  |  |  |  |  |  |  |  |  |  | NSA |
| SIRRIM MRI | Mean total nigral signal intensity | Hu*,* 2006 [98] | 9 |  | NSA |  |  |  |  |  | NSA |  |  |  |
|  | Mean nigral signal intensity for lower nigral slice alone | Hu*,* 2006 [98] | 9 |  | NSA |  |  |  |  |  | NSA |  |  |  |
| Whole brain T2-weighted 2D fast spin echo MRI | Mean intensity score pars reticulata | Atasoy*,* 2004 [90] | 20 | NSA | NSA |  |  |  | NSA | NSA |  |  |  |  |
|  | Mean intensity score red nucleus | Atasoy*,* 2004 [90] | 20 | NSA | NSA |  |  |  | NSA | NSA |  |  |  |  |
| Multiple gradient echo sequence MRI | Contralateral red nucleus R_2_* | Martin*,* 2008 [92] | 22 | NSA |  | NSA |  |  |  |  |  |  |  |  |

| **6.2 MRI - Putamen (PU)** | | | | **Association of measurement with:** | | | | | | | | | | |
| --- | --- | --- | --- | --- | --- | --- | --- | --- | --- | --- | --- | --- | --- | --- |
| **MRI modality** | **Measurement** | **Reference**  **(first author, year)** | **n** | **Total UPDRS** | **UPDRS**  **(III)** | **Lateralized UPDRS (III) from side of body affected:** | | | **UPDRS**  **(II)** | **UPDRS**  **(I)** | **H&Y** | **MMSE** | **S&E** | **TCS** |
|  |  |  |  |  |  | **Most** | **Least** | **Either** |  |  |  |  |  |  |
| Whole brain T2-weighted 2D fast spin echo MRI | Mean intensity score PU | Atasoy*,* 2004 [90] | 20 | NSA | NSA |  |  |  | NSA | NSA |  |  |  |  |
|  | Mean volume score PU | Atasoy*,* 2004 [90] | 20 | NCS | NSA |  |  |  | NSA | NSA |  |  |  |  |
| Multiple gradient echo sequence MRI | Contralateral posterior PU R_2_* | Martin*,* 2008 [92] | 19 | NSA |  | NSA |  |  |  |  |  |  |  |  |
|  | Contralateral anterior PU R_2_* | Martin*,* 2008 [92] | 19 | NSA |  | NSA |  |  |  |  |  |  |  |  |
| PRIME sequence MRI | Contralateral PU R_2_' relaxation rate | Wallis, 2008 [94] | 65 |  |  | NSA | NSA |  |  |  |  |  |  |  |
|  | Mean PU R_2_ | Graham, 2000 [95] | 21 |  | NSA |  |  |  | r = 0.47 |  |  |  |  |  |
|  | Mean PU R_2_* | Graham, 2000 [95] | 21 |  | NSA |  |  |  | NSA |  |  |  |  |  |
|  | Mean PU R_2_' | Graham, 2000 [95] | 21 |  | NSA |  |  |  | NSA |  |  |  |  |  |
| T2-weighted MRI | Total PU T2 value | Antonini, 1993 [97] | 30 |  |  |  |  |  |  |  |  |  |  | NSA |
|  | Total Posterior PU T2 value | Antonini, 1993 [97] | 30 |  |  |  |  |  |  |  |  |  |  | NSA |
| MRI brain – method to measure tissue iron | PU ∆R2' | Ye*,* 1996 [99] | 12 |  | NSA |  |  |  |  |  |  |  | r = -0.76 |  |

| **6.3 MRI - Caudate nucleus (CN)** | | | | **Association of measurement with:** | | | | | | | | | | |
| --- | --- | --- | --- | --- | --- | --- | --- | --- | --- | --- | --- | --- | --- | --- |
| **MRI modality** | **Measurement** | **Reference**  **(first author, year)** | **n** | **Total UPDRS** | **UPDRS**  **(III)** | **Lateralized UPDRS (III) from side of body affected:** | | | **UPDRS**  **(II)** | **UPDRS**  **(I)** | **H&Y** | **MMSE** | **S&E** | **TCS** |
|  |  |  |  |  |  | **Most** | **Least** | **Either** |  |  |  |  |  |  |
| Whole brain T2-weighted 2D fast spin echo MRI | Mean intensity score: CN | Atasoy*,* 2004 [90] | 20 | NSA | NSA |  |  |  | NSA | NSA |  |  |  |  |
| Multiple gradient echo sequence MRI | Contralateral head of CN R_2_* | Martin*,* 2008 [92] | 19 | NSA |  | NSA |  |  |  |  |  |  |  |  |
| PRIME sequence MRI | Mean CN R_2_ | Graham, 2000 [95] | 21 |  | NSA |  |  |  | NSA |  |  |  |  |  |
|  | Mean CN R_2_* | Graham, 2000 [95] | 21 |  | NSA |  |  |  | NSA |  |  |  |  |  |
|  | Mean CN R_2_' | Graham, 2000 [95] | 21 |  | NSA |  |  |  | NSA |  |  |  |  |  |
| T2-weighted MRI | Total CN T2 value | Antonini, 1993 [97] | 30 |  |  |  |  |  |  |  |  |  |  | NSA |

| **6.4 MRI - Globus pallidus (GP)** | | | | **Association of measurement with:** | | | | | | | | | | |
| --- | --- | --- | --- | --- | --- | --- | --- | --- | --- | --- | --- | --- | --- | --- |
| **MRI modality** | **Measurement** | **Reference**  **(first author, year)** | **n** | **Total UPDRS** | **UPDRS**  **(III)** | **Lateralized UPDRS (III) from side of body affected:** | | | **UPDRS**  **(II)** | **UPDRS**  **(I)** | **H&Y** | **MMSE** | **S&E** | **TCS** |
|  |  |  |  |  |  | **Most** | **Least** | **Either** |  |  |  |  |  |  |
| Whole brain T2-weighted 2D fast spin echo MRI | Mean intensity score: GP | Atasoy*,* 2004 [90] | 20 | NSA | NSA |  |  |  | NSA | NSA |  |  |  |  |
| Multiple gradient echo sequence MRI | Contralateral anterior GP R_2_* | Martin*,* 2008 [92] | 19 | NSA |  | NSA |  |  |  |  |  |  |  |  |
|  | Contralateral posterior GP R_2_* | Martin*,* 2008 [92] | 19 | NSA |  | r = 0.56 |  |  |  |  |  |  |  |  |
| PRIME sequence MRI | Mean GP R_2_ | Graham, 2000 [95] | 21 |  | NSA |  |  |  | NSA |  |  |  |  |  |
|  | Mean GP R_2_* | Graham, 2000 [95] | 21 |  | NSA |  |  |  | NSA |  |  |  |  |  |
|  | Mean GP R_2_' | Graham, 2000 [95] | 21 |  | NSA |  |  |  | NSA |  |  |  |  |  |
| T2-weighted scan | Bilateral total GP T2 value | Antonini, 1993 [97] | 30 |  |  |  |  |  |  |  |  |  |  | NSA |
|  | Bilateral GP posterior T2 value | Antonini, 1993 [97] | 30 |  |  |  |  |  |  |  |  |  |  | NSA |
| MRI brain – method to measure tissue iron | GP ∆R2' | Ye*,* 1996 [99] | 12 |  | r = 0.72 |  |  |  |  |  |  |  | r = -0.64 |  |

| **6.5 MRI - Frontal white matter (FWM)** | | | | **Association of measurement with:** | | | | | | | | | | |
| --- | --- | --- | --- | --- | --- | --- | --- | --- | --- | --- | --- | --- | --- | --- |
| **MRI modality** | **Measurement** | **Reference**  **(first author, year)** | **n** | **Total UPDRS** | **UPDRS**  **(III)** | **Lateralized UPDRS (III) from side of body affected:** | | | **UPDRS**  **(II)** | **UPDRS**  **(I)** | **H&Y** | **MMSE** | **S&E** | **TCS** |
|  |  |  |  |  |  | **Most** | **Least** | **Either** |  |  |  |  |  |  |
| PRIME sequence | Mean Frontal white matter R_2_ | Graham, 2000 [95] | 21 |  | NSA |  |  |  | NSA |  |  |  |  |  |
|  | Mean Frontal white matter R_2_* | Graham, 2000 [95] | 21 |  | NSA |  |  |  | NSA |  |  |  |  |  |
|  | Mean Frontal white matter R_2_' | Graham, 2000 [95] | 21 |  | NSA |  |  |  | NSA |  |  |  |  |  |
| T2-weighted scan | Bilateral frontal cortex T2 value | Antonini, 1993 [97] | 30 |  |  |  |  |  |  |  |  |  |  | NSA |

| **6.6 MRI - Other** | | | | **Association of measurement with:** | | | | |
| --- | --- | --- | --- | --- | --- | --- | --- | --- |
| **MRI modality** | **Measurement** | **Reference**  **(first author, year)** | **n** | **UPDRS**  **(III)** | **UPDRS**  **(II)** | **S&E** | **Total CAMCOG** | **TCS** |
|  |  |  |  |  |  |  |  |  |
| Volumetric MRI scan | Amygdala volume | Junque*,* 2005 [100] | 32 |  | r = 0.39 |  |  |  |
|  | Hippocampal volume | Junque*,* 2005 [100] | 32 |  | r = 0.35 |  |  |  |
| T2-weighted scan | Bilateral white matter T2 value | Antonini, 1993 [97] | 30 |  |  |  |  | NSA |
|  | Bilateral CSF T2 value | Antonini, 1993 [97] | 30 |  |  |  |  | NSA |
| T1-weighted 3D scan | Bilateral medial temporal lobe atrophy score (Scheltens Scale) | Tam*,* 2005 [101] | 33† |  | NSA |  | NSA |  |
|  |  | Tam*,* 2005 [101] | 31† |  | NSA |  | NSA |  |
| MRI brain | Appear to have examined 25 different MRI parameters | Linder*,* 2009 [102] | 66 | No difference between those with low and high scores‡ |  |  |  |  |
| MRI brain – method to measure tissue iron | Thalamus ∆R2' | Ye*,* 1996 [99] | 12 | NSA |  | NSA |  |  |

| **6.7 MRS** | | | | **Association of measurement with:** | | |
| --- | --- | --- | --- | --- | --- | --- |
| **MRS modality** | **Measurement** | **Reference**  **(first author, year)** | **n** | **UPDRS**  **(III)** | **H&Y** | **MMSE** |
|  |  |  |  |  |  |  |
| ^1^H – MRS | Adjusted N-acetylaspartate (NAA) concentration in occipital cortex | Summerfield*,* 2002 [103] | 26 | NSA | NSA | NSA |
|  | Adjusted Myoinositol (MI) concentration in occipital cortex | Summerfield*,* 2002 [103] | 26 | NSA | NSA | NSA |
|  | Adjusted Choline (Cho) containing compounds in occipital cortex | Summerfield*,* 2002 [103] | 26 | NSA | NSA | NSA |
|  | N-acetylaspartate /creatine + phosphocreatine ratio (NAA/Cr) in mean temporoparietal cortex | Hu*,* 1999 [104] | 17 | NSA |  |  |

**Key**

† Study examined patients with Parkinson’s disease without dementia (n=33) and Parkinson’s disease with dementia (n=31) separately.

‡ Study looked for any significant differences in MRI between patients with lower (≤20) UPDRS-III scores versus higher (≥21) scores. 25 MRI parameters were evaluated in the paper and although not entirely clear, it appears that no significant difference was found in any of these parameters between these two groups of patients.

^φ^ Study examined patients with Parkinson’s disease without dementia (n=18) and Parkinson’s disease with dementia (n=13) separately.

∆R2' MRI measure which provides an index of local tissue iron content

DTI Diffusion tensor imaging

MRI Magnetic resonance imaging

MRS Magnetic resonance spectroscopy

PRIME Partially refocused interleaved multiple echo MRI sequence

R_2_* Proton transverse relaxation rate

SIRRIM Magnetic resonance segmented inversion recovery ratio imaging

SWI Susceptibility weighted imaging

**Additional file 3 7: *Brain SPECT***

**Associations between putative brain SPECT biomarkers and clinical measures of disease severity, in cross-sectional studies included in the systemic review of biomarkers for disease progression in Parkinson’s disease**

| **7.1 Putamen (PU)** | | | | **Association of ligand binding with:** | | | | | | | | |
| --- | --- | --- | --- | --- | --- | --- | --- | --- | --- | --- | --- | --- |
| **SPECT ligand** | **Target region** | **Reference**  **(first author, year)** | **n** | **Total UPDRS** | **UPDRS**  **(III)** | **Lateralized UPDRS (III)** | **Contra.**  **UPDRS (III)** | **Ipsi. UPDRS (III)** | **UPDRS**  **(II)** | **UPDRS**  **(I)** | **H&Y** | **MMSE** |
| [^123^I]FP-CIT | PU | Benamer*,* 2000 [105] | 41 |  | r = -0.57 |  |  |  |  |  |  |  |
|  |  | Eshuis*,* 2006 [106] | 30 |  | r = -0.45 |  |  |  |  |  | r = -0.51 |  |
|  |  | Tissingh*,* 1998 [107] | 21 |  | NSA |  |  |  |  |  | NSA |  |
|  | Ipsilateral PU | Benamer*,* 2000 [105] | 41 |  | r = -0.62 |  |  |  |  |  |  |  |
|  |  | Eshuis*,* 2006 [106] | 30 |  | r = -0.48 |  |  |  |  |  | r = -0.54 |  |
|  |  | Booij*,* 1997 [108] | 18 |  | NSA |  |  |  |  |  | r = -0.48 |  |
|  | Contralateral PU | Benamer*,* 2000 [105] | 41 |  | r = -0.51 |  |  |  |  |  |  |  |
|  |  | Eshuis*,* 2006 [106] | 30 |  | NSA |  |  |  |  |  | NSA |  |
|  |  | Booij*,* 1997 [108] | 18 |  | NSA |  |  |  |  |  | r = -0.52 |  |
|  | Unilateral PU | Benamer*,* 2000 [105] | 41 |  |  |  | r = -0.39 | r = -0.44 |  |  |  |  |
|  | Less affected hemisphere | Nobili*,* 2010 [109] | 30 |  | r = -0.46 |  |  |  |  |  |  |  |
|  | More affected hemisphere | Nobili*,* 2010 [109] | 30 |  | r = -0.31 |  |  |  |  |  |  |  |
| [^123^I]β-CIT | PU | Winogrodzka*,* 2003 [110] | 50 |  | r = -0.46 |  |  |  |  |  |  |  |
|  |  | Tissingh*,* 1998 [111] | 16 | r = -0.69 | r = -0.71 |  |  |  |  |  |  |  |
|  |  | Muller*,* 2000 [112] | 20 | r = -0.41 |  |  |  |  |  |  | r = -0.46 |  |
|  | (read at 3 hours) | Shinotoh*,* 2000 [113] | 12 |  | NSA |  |  |  |  |  |  |  |
|  | (read at 24 hours) | Shinotoh*,* 2000 [113] | 12 |  | r = -0.82 |  |  |  |  |  |  |  |
|  | Ipsilateral PU | Seibyl*,* 1995 [114] | 28 | r = -0.55 | r = -0.53 |  |  |  |  |  | r = -0.58 |  |

| **7.1 Putamen (PU) (continued)** | | | | **Association of ligand binding with:** | | | | | | | | |
| --- | --- | --- | --- | --- | --- | --- | --- | --- | --- | --- | --- | --- |
| **SPECT ligand** | **Target region** | **Reference**  **(first author, year)** | **n** | **Total UPDRS** | **UPDRS**  **(III)** | **Lateralized UPDRS (III)** | **Contra.**  **UPDRS (III)** | **Ipsi. UPDRS (III)** | **UPDRS**  **(II)** | **UPDRS**  **(I)** | **H&Y** | **MMSE** |
| [^123^I]β-CIT  (continued) | Contralateral PU | Seibyl*,* 1995 [114] | 28 | r = -0.47 | r = -0.47 |  |  |  |  |  | r = -0.52 |  |
|  | Ant PU | Ichise*,* 1999 [115] | 10 |  |  | r = -0.50 |  |  |  |  |  |  |
|  | Post PU | Ichise*,* 1999 [115] | 10 |  |  | r = -0.59 |  |  |  |  |  |  |
| [^123^I]IPT | Ipsilateral PU | Tatsch*,* 1997 [116] | 26 |  |  |  |  |  |  |  | NEG |  |
|  | Contralateral PU | Tatsch*,* 1997 [116] | 26 |  |  |  |  |  |  |  | NEG |  |
| [^123^I]PE2I | PU | Prunier*,* 2003 [117] | 8 |  |  |  |  |  |  |  | r = -0.73 |  |
| [^123^I]IBF | Ant PU | Ichise*,* 1999 [115] | 10 |  |  | NSA |  |  |  |  |  |  |
|  | Post PU | Ichise*,* 1999 [115] | 10 |  |  | NCS |  |  |  |  |  |  |
| [^99m^Tc]  TRODAT-1 | PU | Geng*,* 2005 [118] | 38 | NEG |  |  |  |  |  |  | NEG |  |
|  |  | Huang*,* 2004 [119] | 188 |  |  |  |  |  |  |  | r = -0.93 |  |
|  |  | Siderowf*,* 2005 [120] | 24 |  | NSA |  |  |  |  |  |  |  |
|  |  | Huang*,* 2001 [121] | 34 |  |  |  |  |  |  |  | Uptake less in H&Y 2 than 1 |  |
|  | Contralateral PU | Weng*,* 2004 [122] | 78 | NEG |  |  |  |  |  |  | NEG |  |
|  | Ipsilateral PU | Weng*,* 2004 [122] | 78 | NEG |  |  |  |  |  |  | NEG |  |

| **7.2 Caudate nucleus (CN)** | | | | **Association of ligand binding with:** | | | | | | | | |
| --- | --- | --- | --- | --- | --- | --- | --- | --- | --- | --- | --- | --- |
| **SPECT ligand** | **Target region** | **Reference**  **(first author, year)** | **n** | **Total UPDRS** | **UPDRS**  **(III)** | **Lateralized UPDRS (III)** | **Contra.**  **UPDRS (III)** | **Ipsi. UPDRS (III)** | **UPDRS**  **(II)** | **UPDRS**  **(I)** | **H&Y** | **MMSE** |
| [^123^I]FP-CIT | CN | Benamer*,* 2000 [105] | 41 |  | r = -0.50 |  |  |  |  |  |  |  |
|  |  | Eshuis*,* 2006 [106] | 30 |  | r = -0.47 |  |  |  |  |  | r = -0.49 |  |
|  |  | Tissingh*,* 1998 [107] | 21 |  | NSA |  |  |  |  |  | NSA |  |
|  | Ipsilateral CN | Benamer*,* 2000 [105] | 41 |  | r = -0.50 |  |  |  |  |  |  |  |
|  |  | Eshuis*,* 2006 [106] | 30 |  | r = -0.43 |  |  |  |  |  | r = -0.51 |  |
|  |  | Booij*,* 1997 [108] | 18 |  | NSA |  |  |  |  |  | r = -0.49 |  |
|  | Contralateral CN | Benamer*,* 2000 [105] | 41 |  | r = -0.51 |  |  |  |  |  |  |  |
|  |  | Eshuis*,* 2006 [106] | 30 |  | NCS |  |  |  |  |  | NSA |  |
|  |  | Booij*,* 1997 [108] | 18 |  | NSA |  |  |  |  |  | r = -0.58 |  |
|  | Unilateral CN | Benamer*,* 2000 [105] | 41 |  |  |  | r = -0.36 | r = -0.34 |  |  |  |  |
|  | More affected hemisphere | Nobili*,* 2010 [109] | 30 |  | r = -0.31 |  |  |  |  |  |  |  |
| [^123^I]β-CIT | CN | Winogrodzka, 2003 [110] | 50 |  | r = -0.52 |  |  |  |  |  |  |  |
|  |  | Tissingh, 1998 [111] | 16 | r = -0.81 | r = -0.89 |  |  |  |  |  |  |  |
|  |  | Ichise, 1999 [115] | 10 |  |  | r = -0.49 |  |  |  |  |  |  |
|  | (read at 3 hours) | Shinotoh, 2000 [113] | 12 |  | NSA |  |  |  |  |  |  |  |
|  | (read at 24 hours) | Shinotoh, 2000 [113] | 12 |  | r = -0.76 |  |  |  |  |  |  |  |
|  | Head of CN | Muller, 2000 [112] | 20 | NSA |  |  |  |  |  |  | NSA |  |
|  | Ipsilateral CN | Seibyl*,* 1995 [114] | 28 | r = -0.48 | r = -0.47 |  |  |  |  |  | r = -0.51 |  |
|  | Contralateral CN | Seibyl*,* 1995 [114] | 28 | r = -0.49 | r = -0.48 |  |  |  |  |  | r = -0.48 |  |

| **7.2 Caudate nucleus (CN) (continued)** | | | | **Association of ligand binding with:** | | | | | | | | |
| --- | --- | --- | --- | --- | --- | --- | --- | --- | --- | --- | --- | --- |
| **SPECT ligand** | **Target region** | **Reference**  **(first author, year)** | **n** | **Total UPDRS** | **UPDRS**  **(III)** | **Lateralized UPDRS (III)** | **Contra.**  **UPDRS (III)** | **Ipsi. UPDRS (III)** | **UPDRS**  **(II)** | **UPDRS**  **(I)** | **H&Y** | **MMSE** |
| [^123^I]IPT | Ipsilateral CN | Tatsch, 1997 [116] | 26 |  |  |  |  |  |  |  | NEG |  |
|  | Contralateral CN | Tatsch, 1997 [116] | 26 |  |  |  |  |  |  |  | NEG |  |
| [^123^I]PE2I | CN | Prunier, 2003 [117] | 8 |  |  |  |  |  |  |  | NSA |  |
| [^123^I]IBF | CN | Ichise*,* 1999 [115] | 10 |  |  | NSA |  |  |  |  |  |  |
| [^99m^Tc]  TRODAT-1 | CN | Geng, 2005 [118] | 38 | NEG |  |  |  |  |  |  | NSA |  |
|  |  | Siderowf, 2005 [120] | 24 |  | NSA |  |  |  |  |  |  |  |
|  | Contralateral CN | Weng, 2004 [122] | 78 | NEG |  |  |  |  |  |  | NEG |  |
|  | Ipsilateral CN | Weng, 2004 [122] | 78 | NEG |  |  |  |  |  |  | NEG |  |

| **7.3 Striatum (ST)** | | | | **Association of ligand binding with:** | | | | | | | | |
| --- | --- | --- | --- | --- | --- | --- | --- | --- | --- | --- | --- | --- |
| **SPECT ligand** | **Target region** | **Reference**  **(first author, year)** | **n** | **Total UPDRS** | **UPDRS**  **(III)** | **Lateralized UPDRS (III)** | **Contra.**  **UPDRS (III)** | **Ipsi. UPDRS (III)** | **UPDRS**  **(II)** | **UPDRS**  **(I)** | **H&Y** | **MMSE** |
| [^123^I]FP-CIT | ST | Benamer, 2000 [105] | 41 |  | r = -0.54 |  |  |  |  |  |  |  |
|  |  | Eshuis, 2006 [106] | 30 |  | r = -0.45 |  |  |  |  |  | r = -0.52 |  |
|  |  | Ottaviani, 2006 [123] | 85 | r = -0.28 |  |  |  |  |  |  | r = -0.43 |  |
|  |  | Tissingh, 1998 [107] | 21 |  | NSA |  |  |  |  |  | NSA |  |
|  | Ipsilateral ST | Benamer, 2000 [105] | 41 |  | r = -0.57 |  |  |  |  |  |  |  |
|  |  | Eshuis, 2006 [106] | 30 |  | r = -0.48 |  |  |  |  |  | r = -0.55 |  |
|  |  | Booij, 1997 [108] | 18 |  | NSA |  |  |  |  |  | r = -0.50 |  |
|  | Contralateral ST | Benamer, 2000 [105] | 41 |  | r = -0.50 |  |  |  |  |  |  |  |
|  |  | Eshuis, 2006 [106] | 30 |  | NSA |  |  |  |  |  | NSA |  |
|  |  | Booij, 1997 [108] | 18 |  | NSA |  |  |  |  |  | r = -0.59 |  |
|  |  | Spiegel, 2005 [86] | 18 |  | r = -0.51 |  |  |  |  |  |  |  |
| [^123^I]β-CIT | ST | Weise, 2009 [75] | 50 |  |  |  |  |  |  |  | r = -0.50 |  |
|  |  | Haapaniemi*,* 2001 [124]† | 29 | r = -0.56 | r = -0.58 |  |  |  | r = -0.47 |  | r = -0.49 |  |
|  |  | Shinotoh, 2000 [113] | 12 |  | r = -0.68 |  |  |  |  |  |  |  |
|  |  | Brucke*,* 1997 [125] | 80 |  | r = -0.42 |  |  |  | r = -0.55 |  | r = -0.66 |  |
|  |  | Kim*,* 1999 [126] | 46 | r = -0.51 | r = -0.47 |  |  |  | r = -0.53 | NSA | r = -0.50 |  |
|  |  | Tissingh, 1998 [111] | 16 | r = -0.79 | r = -0.81 |  |  |  |  |  |  |  |
|  |  | Winogrodzka, 2003 [110] | 50 |  | r = -0.51 |  |  |  |  |  |  |  |
|  | Ipsilateral ST | Tissingh, 1998 [111] | 16 |  | r = -0.79 |  |  |  |  |  |  |  |
|  | Contralateral ST | Tissingh, 1998 [111] | 16 |  | r = -0.86 |  |  |  |  |  |  |  |
|  | Unilateral ST | Kim*,* 1999 [126] | 46 |  |  |  | r = -0.42 |  |  |  |  |  |

| **7.3 Striatum (ST) (continued)** | | | | **Association of ligand binding with:** | | | | | | | | |
| --- | --- | --- | --- | --- | --- | --- | --- | --- | --- | --- | --- | --- |
| **SPECT ligand** | **Target region** | **Reference**  **(first author, year)** | **n** | **Total UPDRS** | **UPDRS**  **(III)** | **Lateralized UPDRS (III)** | **Contra.**  **UPDRS (III)** | **Ipsi. UPDRS (III)** | **UPDRS**  **(II)** | **UPDRS**  **(I)** | **H&Y** | **MMSE** |
| [^123^I]IPT | Ipsilateral ST | Tatsch, 1997 [116] | 26 |  |  |  |  |  |  |  | NEG |  |
|  | Contralateral ST | Tatsch, 1997 [116] | 26 |  |  |  |  |  |  |  | NEG |  |
| [^123^I]PE2I | ST | Prunier, 2003 [117] | 8 |  |  |  |  |  |  |  | NSA |  |
| [^99m^Tc]  TRODAT-1 | ST | Geng, 2005 [118] | 38 | NEG |  |  |  |  |  |  | NSA |  |
|  |  | Siderowf, 2005 [120] | 24 |  | NSA |  |  |  |  |  |  |  |
|  |  | Hwang*,* 2004 [127]‡ | 20 |  | r = -0.53 |  |  |  |  |  |  |  |
|  |  | Hwang*,* 2004 [127]‡ | 20 |  | r = -0.48 |  |  |  |  |  |  |  |
|  |  | Bao*,* 2000 [128] | 27 | r = -0.60 | r = -0.44 |  |  |  |  |  | r = -0.74 |  |
|  |  | Huang, 2004 [119] | 188 |  |  |  |  |  |  |  | r = -0.89 |  |
|  |  | Huang, 2001 [121] | 34 |  |  |  |  |  |  |  | Uptake less in H&Y 2 than 1 |  |
| [^99m^Tc]ECD | Right ST | Derejko*,* 2006 [129] | 60 |  |  |  |  |  |  |  | τ = -0.20 |  |

| **7.4 Other** | | | | **Association of ligand binding with:** | | | | | | | | |
| --- | --- | --- | --- | --- | --- | --- | --- | --- | --- | --- | --- | --- |
| **SPECT ligand** | **Target region** | **Reference**  **(first author, year)** | **n** | **Total UPDRS** | **UPDRS**  **(III)** | **Lateralized UPDRS (III)** | **Contra.**  **UPDRS (III)** | **Ipsi. UPDRS (III)** | **UPDRS**  **(II)** | **UPDRS**  **(I)** | **H&Y** | **MMSE** |
| [^123^I]FP-CIT | Ipsilateral PU/CN ratio | Booij,1997 [108] | 18 |  | NSA |  |  |  |  |  | NSA |  |
|  | Contralateral PU/CN ratio | Booij,1997 [108] | 18 |  | NSA |  |  |  |  |  | NSA |  |
| [^123^I]β-CIT | Ipsilateral PU/CN ratio | Seibyl, 1995 [114] | 28 | r = -0.39 | NSA |  |  |  |  |  | NSA |  |
|  | Contralateral PU/CN ratio | Seibyl, 1995 [114] | 28 | NSA | NSA |  |  |  |  |  | NSA |  |
|  | Medial frontal region (SERT uptake) | Haapaniemi, 2001 [124]† | 29 |  |  |  |  |  |  | r = -0.42 |  |  |
|  | Hypothalamic/midbrain region | Kim, 1999 [126] | 46 | NSA | NSA |  |  |  | NSA | NSA | NSA |  |
| [^123^I]IMP | Brodmann area 11 | Matsui*,* 2005 [130] | 55 |  | NSA |  |  |  |  |  | NSA | NSA |
| [^99m^Tc]ECD | Prefrontal cortex  Right  Left | Derejko*,* 2006 [129] | 60 |  |  |  |  |  |  |  | τ = -0.24 |  |
|  |  | Derejko*,* 2006 [129] | 60 |  |  |  |  |  |  |  | τ = -0.27 |  |
|  | Frontal cortex  Right  Left | Derejko*,* 2006 [129] | 60 |  |  |  |  |  |  |  | τ = -0.23 |  |
|  |  | Derejko*,* 2006 [129] | 60 |  |  |  |  |  |  |  | τ = -0.26 |  |
|  | Left parietal cortex | Derejko*,* 2006 [129] | 60 |  |  |  |  |  |  |  | τ = -0.34 |  |
|  | Left temporal cortex | Derejko*,* 2006 [129] | 60 |  |  |  |  |  |  |  | τ = -0.22 |  |

| **7.4 Other (continued)** | | | | **Association of ligand binding with:** | | | | | | | | |
| --- | --- | --- | --- | --- | --- | --- | --- | --- | --- | --- | --- | --- |
| **SPECT ligand** | **Target region** | **Reference**  **(first author, year)** | **n** | **Total UPDRS** | **UPDRS**  **(III)** | **Lateralized UPDRS (III)** | **Contra.**  **UPDRS (III)** | **Ipsi. UPDRS (III)** | **UPDRS**  **(II)** | **UPDRS**  **(I)** | **H&Y** | **MMSE** |
| [^99m^Tc]ECD (continued) | Left primary motor cortex and supplementary motor area | Paschali*,* 2010 [131] | 53 |  | r = -0.61 |  |  |  |  |  |  |  |
|  | Expression of SPECT-derived Parkinson's disease related pattern (PDRP) | Feigin*,* 2002 [132] | 23 |  | r = 0.44 |  |  |  |  |  |  |  |
|  | Expression of PET-derived Parkinson's disease related pattern (PET-derived PDRP) | Feigin*,* 2002 [132] | 23 |  | r = 0.52 |  |  |  |  |  |  |  |
| HMPAO | Ant cingulate gyrus  Right  Left | Hsu*,* 2007 [133] | 27 |  | NEG |  |  |  |  |  |  |  |
|  |  | Hsu*,* 2007 [133] | 27 |  | r = -0.75 |  |  |  |  |  |  |  |
|  | Cingulate gyrus  Right  Left | Hsu*,* 2007 [133] | 27 |  | NEG |  |  |  |  |  |  |  |
|  |  | Hsu*,* 2007 [133] | 27 |  | NEG |  |  |  |  |  |  |  |
|  | Postcentral gyrus  Right  Left | Hsu*,* 2007 [133] | 27 |  | NEG |  |  |  |  |  |  |  |
|  |  | Hsu*,* 2007 [133] | 27 |  | NEG |  |  |  |  |  |  |  |
|  | Parahippocampal gyrus  Right  Left | Hsu*,* 2007 [133] | 27 |  | NEG |  |  |  |  |  |  |  |
|  |  | Hsu*,* 2007 [133] | 27 |  | NEG |  |  |  |  |  |  |  |

| **7.4 Other (continued)** | | | | **Association of ligand binding with:** | | | | | | | | |
| --- | --- | --- | --- | --- | --- | --- | --- | --- | --- | --- | --- | --- |
| **SPECT ligand** | **Target region** | **Reference**  **(first author, year)** | **n** | **Total UPDRS** | **UPDRS**  **(III)** | **Lateralized UPDRS (III)** | **Contra.**  **UPDRS (III)** | **Ipsi. UPDRS (III)** | **UPDRS**  **(II)** | **UPDRS**  **(I)** | **H&Y** | **MMSE** |
| HMPAO (continued) | Right insula | Hsu*,* 2007 [133] | 27 |  | NEG |  |  |  |  |  |  |  |
|  | Right inferior parietal lobule | Hsu*,* 2007 [133] | 27 |  | NEG |  |  |  |  |  |  |  |
|  | Supplementary motor area  Right | Kikuchi*,* 2001 [134] | 18 | NSA |  |  |  |  |  |  | No significant difference (H&Y 1-2 versus 3-4) |  |
|  | Left | Kikuchi*,* 2001 [134] | 18 | NSA |  |  |  |  |  |  | No significant difference (H&Y 1-2 versus 3-4) |  |
|  | Dorsolateral prefrontal cortex  Right | Kikuchi*,* 2001 [134] | 18 | NSA |  |  |  |  |  |  | Lower rCBF in H&Y 3-4 than 1-2 |  |
|  | Left | Kikuchi*,* 2001 [134] | 18 | r = -0.66 |  |  |  |  |  |  | Lower rCBF in H&Y 3-4 than 1-2 |  |
|  | Insular cortex  Right    Left | Kikuchi*,* 2001 [134] | 18 | NSA  r = -0.74 |  |  |  |  |  |  | Lower rCBF in H&Y 3-4 than 1-2  Lower rCBF in H&Y 3-4 than 1-2 |  |

Uptake values on the same half of the body as the dominant (and generally initial) side of motor symptoms were called ipsilateral values and those opposite to that side contralateral.

In many papers it was difficult, if not impossible, to tell whether Associations with clinical measures of disease severity were examined using the total value of uptake of a structure present bilaterally (i.e. uptake in right and left caudate summed together to give value for the whole caudate), or to the mean value of the structure (i.e. mean of uptake in right and left caudate). Therefore for simplicity these values are simply grouped together in the above tables without separating mean or total values. Therefore where a value is neither described as being ipsilateral, contralateral or unilateral then it can be assumed it is either a mean or a total value for the structure in question.

**Key**

† This study calculated specific striatal dopamine active transporter (DAT) binding when a state of equilibrium was reached at 20-24 hours post-injection of ligand. In addition regions of interest for monoamine transporters, mainly reflecting serotonin transporters (SERT), were examined, including the medial prefrontal area at the striatal level. At four hours post injection using these regions of interest SERT specific-to-non-displaceable binding in the medial frontal area was calculated.

‡ Longitudinal study but no relevant longitudinal correlations. Also interval between scans only two to three weeks as study was undertaken to look at reproducibility of scan results. Therefore two cross-sectional correlations for the initial and second scan are given rather than a longitudinal correlation.

**SPECT ligands**

[^123^I]FP-CIT [^123^I]-2β-carbomethoxy-3β-(4-iodophenyl)-N-(3-fluoropropyl)-N-tropane

[^123^I]IBF [^123^I]-(5-iodo-7-N-((1-ethyl-2pyrrolidinyl)methyl)carboxamido-2, 3-dihydrobenzofuran)

[^123^I]IPT [^123^I]-N-(3-iodopropen-2-yl)-2β-carbomethoxy-3β-(4-chlorophenyl) tropane

[^123^I]PE2I [^123^I]- N-(3-iodoprop-2E-enyl)-2β-carbomethoxy-3β-(4-methylphenyl) nortropane

[^123^I]β-CIT [^123^I]-2β-carbomethoxy-3β-(4-iodophenyl tropane)

[^99m^Tc]ECD [^99m^Tc]-ethyl cysteinate dimer

[^99m^Tc]TRODAT-1 [^99m^Tc]-2β-((N,N’-bis(2-mercaptoethyl)ethylene diamino)methyl), 3β-(4-chlorphenyl)tropane)

HMPAO [^99m^Tc]-hexamethylpropylene amine oxidase

**Additional file 3 *8: Brain PET***

**Associations between putative brain PET biomarkers and clinical measures of disease severity, in cross-sectional studies included in the systemic review of biomarkers for disease progression in Parkinson’s disease**

| **8.1 Putamen (PU)** | | | | **Association of ligand binding with:** | | | | | |
| --- | --- | --- | --- | --- | --- | --- | --- | --- | --- |
| **PET ligand** | **Target region** | **Reference**  **(first author, year)** | **n** | **Total UPDRS** | **UPDRS**  **(III)** | **Contra.**  **UPDRS (III)** | **H&Y** | **S&E** | **CURS** |
| FDOPA | PU | Eshuis, 2006 [106] | 30 |  | NSA |  | r = -0.54 |  |  |
|  |  | Ribeiro*,* 2002 [135] | 18 |  | r = -0.78 |  |  |  |  |
|  |  | Antonini*,* 1995 [136] | 20 |  |  |  | r = -0.77 |  |  |
|  |  | Morrish*,* 1995 [137] | 27 | r = -0.41 | r = -0.39 |  |  |  |  |
|  |  | Nagasawa*,* 1993 [138] | 10 |  |  |  | NEG |  |  |
|  |  | Vingerhoets*,* 1997 [139] | 35 |  |  |  |  |  | r = -0.61 |
|  |  | Broussolle*,* 1999 [140] | 27 |  | r = -0.47 |  | r = -0.53 |  |  |
|  |  | Brooks*,* 1990 [141] | 16 |  |  |  | r = -0.56 |  |  |
|  |  | Hu, 2006 [98] | 9 |  | NSA |  | NSA |  |  |
|  |  | Otsuka*,* 1996 [142]‡ | 17 |  |  |  | Lower uptake in H&Y 3 than 1 |  |  |
|  |  | Nagasawa*,* 1996 [143] | 20 |  |  |  | NEG (more marked in late than early onset (both n = 10) group) |  |  |
|  |  |  |  |  |  |  |  |  |  |
|  | Unilateral PU | Morrish*,* 1995 [137] | 27 |  |  | r = -0.49 |  |  |  |
|  | Ipsilateral PU | Eshuis, 2006 [106] | 30 |  | r = -0.46 |  | r = -0.62 |  |  |
|  | Contralateral PU | Eshuis, 2006 [106] | 30 |  | NSA |  | NSA |  |  |
|  | Left PU | Holthoff-Detto*,* 1997 [144] | 20 |  |  |  | r = -0.6 |  |  |
|  | Right PU | Holthoff-Detto*,* 1997 [144] | 20 |  |  |  | r = -0.6 |  |  |
|  | Ant PU | Hu, 2006 [98] | 9 |  | NSA |  | NSA |  |  |
|  | Post PU | Hu, 2006 [98] | 9 |  | NSA |  | NSA |  |  |

| **8.1 Putamen (PU) (continued)** | | | | **Association of ligand binding with:** | | | | | |
| --- | --- | --- | --- | --- | --- | --- | --- | --- | --- |
| **PET ligand** | **Target region** | **Reference**  **(first author, year)** | **n** | **Total UPDRS** | **UPDRS**  **(III)** | **Contra.**  **UPDRS (III)** | **H&Y** | **S&E** | **CURS** |
| FDOPA (continued) | Dorsal PU | Hu, 2006 [98] | 9 |  | NSA |  | NSA |  |  |
|  | Ventral PU | Hu, 2006 [98] | 9 |  | NSA |  | NSA |  |  |
|  | Unilateral PU  Most affected PU | Nandhagopal*,* 2009 [145] | 78 |  | r = -0.43† |  |  |  |  |
|  | Least affected PU | Nandhagopal*,* 2009 [145] | 78 |  | r = -0.49† |  |  |  |  |
| [^18^F]FP-CIT | Ant PU | Wang*,* 2007 [146] | 41 |  | r = -0.53 |  |  |  |  |
|  | Post PU | Wang*,* 2007 [146] | 41 |  | r = -0.61 |  |  |  |  |
| [^11^C]DTBZ | Ant PU | Bohnen*,* 2006 [147] | 31 |  | NSA |  | NSA | r = -0.37 |  |
|  | Post PU | Bohnen*,* 2006 [147] | 31 |  | NSA |  | NSA | NSA |  |
|  | Ipsilateral Ant PU | Martin*,* 2008 [148] | 27 |  | NSA | r = -0.46 |  |  |  |
|  | Ipsilateral Midputamen | Martin*,* 2008 [148] | 27 |  | r = -0.51 | r = -0.58 |  |  |  |
|  | Ipsilateral Post PU | Martin*,* 2008 [148] | 27 |  | r = -0.43 | r = -0.48 |  |  |  |
|  | Unilateral PU  Most affected PU | Nandhagopal*,* 2009 [145] | 78 |  | NSA† |  |  |  |  |
|  | Least affected PU | Nandhagopal*,* 2009 [145] | 78 |  | r = -0.29† |  |  |  |  |
| [^11^C]dMP | PU | Breit*,* 2006 [149] | 20 |  | r = -0.79 |  |  |  |  |

| **8.1 Putamen (PU) (continued)** | | | | **Association of ligand binding with:** | | | | | |
| --- | --- | --- | --- | --- | --- | --- | --- | --- | --- |
| **PET ligand** | **Target region** | **Reference**  **(first author, year)** | **n** | **Total UPDRS** | **UPDRS**  **(III)** | **Contra.**  **UPDRS (III)** | **H&Y** | **S&E** | **CURS** |
| 2-[^18^F]fluoro-A-85380 | PU | Kas*,* 2009 [150] | 13 |  | NSA |  |  |  |  |
| [^76^Br]FE-CBT | PU | Ribeiro, 2002 [135] | 18 |  | NSA |  |  |  |  |
| [^11^C]RAC | PU | Antonini, 1995 [136] | 20 |  |  |  | r = -0.52 |  |  |
|  |  | Rinne*,* 1995 [151] | 10 |  | NSA |  |  |  |  |
| FDG | PU | Antonini, 1995 [136] | 20 |  |  |  | NSA |  |  |
|  |  | Holthoff-Detto, 1997 [144] | 20 |  |  |  | NSA |  |  |

| **8.2 Caudate nucleus (CN)** | | | | **Association of ligand binding with:** | | | | | |
| --- | --- | --- | --- | --- | --- | --- | --- | --- | --- |
| **PET ligand** | **Target region** | **Reference**  **(first author, year)** | **n** | **Total UPDRS** | **UPDRS**  **(III)** | **Contra.**  **UPDRS (III)** | **H&Y** | **S&E** | **CURS** |
| FDOPA | CN | Eshuis, 2006 [106] | 30 |  | NSA |  | r = -0.44 |  |  |
|  |  | Antonini, 1995 [136] | 20 |  |  |  | r = -0.57 |  |  |
|  |  | Nagasawa, 1993 [138] | 10 |  |  |  | NEG |  |  |
|  |  | Vingerhoets, 1997 [139] | 35 |  |  |  |  |  | r = -0.53 |
|  |  | Broussolle, 1999 [140] | 27 |  | NSA |  | r = -0.51 |  |  |
|  |  | Brooks, 1990 [141] | 16 |  |  |  | r = -0.65 |  |  |
|  |  | Hu, 2006 [98] | 9 |  | NSA |  | NSA |  |  |
|  |  | Otsuka, 1996 [142]‡ | 17 |  |  |  | Lower uptake in H&Y 3 than 1 |  |  |
|  |  | Nagasawa, 1996 [143] | 20 |  |  |  | NEG (more marked in late than early onset (both n = 10) group) |  |  |
|  | Ipsilateral CN | Eshuis, 2006 [106] | 30 |  | r = -0.43 |  | r = -0.53 |  |  |
|  | Contralateral CN | Eshuis, 2006 [106] | 30 |  | NSA |  | NSA |  |  |

| **8.2 Caudate nucleus (CN) (continued)** | | | | **Association of ligand binding with:** | | | | | |
| --- | --- | --- | --- | --- | --- | --- | --- | --- | --- |
| **PET ligand** | **Target region** | **Reference**  **(first author, year)** | **n** | **Total UPDRS** | **UPDRS**  **(III)** | **Contra.**  **UPDRS (III)** | **H&Y** | **S&E** | **CURS** |
| FDOPA  (continued) | Left CN | Holthoff-Detto, 1997 [144] | 20 |  |  |  | NSA |  |  |
|  | Right CN | Holthoff-Detto, 1997 [144] | 20 |  |  |  | NSA |  |  |
| [^18^F]FP-CIT | CN | Wang, 2007 [146] | 41 |  | r = -0.53 |  |  |  |  |
| [^11^C]DTBZ | CN | Bohnen, 2006 [147] | 31 |  | NSA |  | NSA | r = -0.40 |  |
|  | Ipsilateral CN | Martin, 2008 [148] | 27 |  | NSA | r = -0.41 |  |  |  |
| [^11^C]dMP | CN | Breit, 2006 [149] | 20 |  | r = -0.77 |  |  |  |  |
| 2-[^18^F]fluoro-A-85380 | CN | Kas, 2009 [150] | 13 |  | NSA |  |  |  |  |
| [^11^C]RAC | CN | Antonini, 1995 [136] | 20 |  |  |  | r = -0.63 |  |  |
|  |  | Rinne, 1995 [151] | 10 |  | NSA |  |  |  |  |
| FDG | CN | Antonini, 1995 [136] | 20 |  |  |  | NSA |  |  |
|  |  | Holthoff-Detto, 1997 [144] | 20 |  |  |  | NSA |  |  |

| **8.3 Striatum (ST)** | | | | **Association of ligand binding with:** | | | | | |
| --- | --- | --- | --- | --- | --- | --- | --- | --- | --- |
| **PET ligand** | **Target region** | **Reference**  **(first author, year)** | **n** | **Total UPDRS** | **UPDRS**  **(III)** | **Contra.**  **UPDRS (III)** | **H&Y** | **S&E** | **CURS** |
| FDOPA | ST | Eshuis, 2006 [106] | 30 |  | NSA |  | r = -0.52 |  |  |
|  |  | Vingerhoets, 1997 [139] | 35 |  |  |  |  |  | r = -0.64 |
|  | ST (uptake calculated using plasma FDOPA time-activity curve) | Takikawa*,* 1994 [152] | 12 |  | r = -0.60 |  | r = -0.68 |  |  |
|  | ST (uptake calculated using occipital time-activity curve) | Takikawa*,* 1994 [152] | 12 |  | NSA |  | r = -0.61 |  |  |
|  | ST (uptake calculated using a simplified population-derived FDOPA input function) | Takikawa*,* 1994 [152] | 12 |  | NSA |  | r = -0.63 |  |  |
|  | ST (uptake calculated using plasma ^18^F time-activity curve) | Takikawa*,* 1994 [152] | 12 |  | NSA |  | NSA |  |  |
|  | Ipsilateral ST | Eshuis, 2006 [106] | 30 |  | r = -0.47 |  | r = -0.62 |  |  |
|  | Contralateral ST | Eshuis, 2006 [106] | 30 |  | NSA |  | NSA |  |  |
| [^11^C]DTBZ | ST | Bohnen, 2006 [147] | 31 |  | NSA |  | NSA | r = -0.38 |  |

| **8.4 Other** | | | | **Association of ligand binding with:** | | | | | |
| --- | --- | --- | --- | --- | --- | --- | --- | --- | --- |
| **PET ligand** | **Target region** | **Reference**  **(first author, year)** | **n** | **Total UPDRS** | **UPDRS**  **(III)** | **Contra.**  **UPDRS (III)** | **H&Y** | **S&E** | **CURS** |
| FDOPA | Caudate-putamen index | Otsuka, 1996 [142]‡ | 17 |  |  |  | NSA |  |  |
|  | Striato-occipital ratio | Takikawa, 1994 [152] | 12 |  | NSA |  | NSA |  |  |
| 2-[^18^F]fluoro-A-85380 | Frontal cortex | Kas, 2009 [150] | 13 |  | NSA |  |  |  |  |
|  | Parietal cortex | Kas, 2009 [150] | 13 |  | NSA |  |  |  |  |
|  | Occipital cortex | Kas, 2009 [150] | 13 |  | NSA |  |  |  |  |
|  | Temporal cortex | Kas, 2009 [150] | 13 |  | NSA |  |  |  |  |
|  | Cerebellum | Kas, 2009 [150] | 13 |  | NSA |  |  |  |  |
|  | Corpus callosum | Kas, 2009 [150] | 13 |  | NSA |  |  |  |  |
|  | Thalamus | Kas, 2009 [150] | 13 |  | NSA |  |  |  |  |
|  | Substantia nigra | Kas, 2009 [150] | 13 |  | NSA |  |  |  |  |
|  | Pons | Kas, 2009 [150] | 13 |  | NSA |  |  |  |  |
|  | Hippocampus | Kas, 2009 [150] | 13 |  | NSA |  |  |  |  |
|  | Amygdala | Kas, 2009 [150] | 13 |  | NSA |  |  |  |  |
| [^11^C]RAC | Hypothalamus | Politis*,* 2008 [153] | 14 |  | NSA |  |  |  |  |

| **8.4 Other (continued)** | | | | **Association of ligand binding with:** | | | | | |
| --- | --- | --- | --- | --- | --- | --- | --- | --- | --- |
| **PET ligand** | **Target region** | **Reference**  **(first author, year)** | **n** | **Total UPDRS** | **UPDRS**  **(III)** | **Contra.**  **UPDRS (III)** | **H&Y** | **S&E** | **CURS** |
| FDG | Expression of principle component 1 pattern | Lozza*,* 2004 [154] | 15 |  |  |  |  |  | r = 0.64 |
|  | Thalamus | Holthoff-Detto, 1997 [144] | 20 |  |  |  | NSA |  |  |
|  | Hippocampus | Holthoff-Detto, 1997 [144] | 20 |  |  |  | NSA |  |  |
|  | Precentral cortex | Holthoff-Detto, 1997 [144] | 20 |  |  |  | NSA |  |  |
|  | Prefrontal cortex | Holthoff-Detto, 1997 [144] | 20 |  |  |  | NSA |  |  |
|  | Dorsolateral prefrontal  Cortex | Holthoff-Detto, 1997 [144] | 20 |  |  |  | NSA |  |  |
|  | Orbitofrontal cortex | Holthoff-Detto, 1997 [144] | 20 |  |  |  | NSA |  |  |
| [^11^C]WAY 100635 | Raphe nucleus | Doder*,* 2003 [155] | 23 |  | NSA |  |  |  |  |

Uptake values on the same half of the body as the dominant (and generally initial) side of motor symptoms were called ipsilateral values and those opposite to that side contralateral.

In many papers it was difficult, if not impossible, to tell whether Associations with clinical measures of disease severity were examined using the total value of uptake of a structure present bilaterally (i.e. uptake in right and left caudate summed together to give value for the whole caudate), or to the mean value of the structure (i.e. mean of uptake in right and left caudate). Therefore for simplicity these values are simply grouped together in the above tables without separating mean or total values. Therefore where a value is neither described as being ipsilateral, contralateral or unilateral then it can be assumed it is either a mean or a total value for the structure in question.

**Key**

† This study was longitudinal in design, with multi-tracer PET at three time points. However, the only relevant correlations are cross-sectional in nature, examining the Association between the appearances of the first scan and UPDRS (III). The article simply states that on the more affected side, these correlations were not maintained across visits.

‡ This study examined for correlations between H&Y and uptake in intracranial structures on the side contralateral to the affected body side in eight cases demonstrating asymmetry, and the mean uptake over both sides in the other nine cases.

**PET ligands**

[^11^C]dMP [^11^C]d-threo-methylphenidate

[^11^C]DTBZ [^11^C]-dihydroxytetrabenazine

[^11^C]RAC [^11^C]-raclopride

[^11^C]WAY 100635 [*Carbonyl*-^11^C]*N*-(2-(1-(4-(2-Methoxyphenyl)-piperazinyl)ethyl)-*N*-pyridinyl)cyclohexanecarboxamide

[^18^F]FP-CIT [^18^F]-2β-carbomethoxy-3β-(4-iodophenyl)-N-(3-fluoropropyl)-N-tropane

[^76^Br]FE-CBT [^76^Br]-(fluorethyl-methyl-2β-carboxymethoxy-3β-4-bromophenyl-tropane)

2-[18F]fluoro-A-85380 2-[^18^F]-fluoro-3-[2(*S*)-2-azetidinylmethoxy]pyridine

FDG [^18^F]-2-fluoro-2-deoxyglucose

FDOPA [^18^F]6-fluoro-L-3,4-dihydroxyphenylalanine

**Additional file 3 *9: Electrophysiology***

**Associations between putative electrophysiological biomarkers and clinical measures of disease severity, in cross-sectional studies included in the systemic review of biomarkers for disease progression in Parkinson’s disease**

| **Electrophysiological** | |  |  | **Association of electrophysiological feature measured with:** | | | | | |
| --- | --- | --- | --- | --- | --- | --- | --- | --- | --- |
| **Modality** | **Feature measured** | **Reference**  **(first author, year)** | **n** | **Total UPDRS** | **UPDRS**  **(III)** | **H&Y** | **MMSE** | **WDS** | **SCOPA-AUT** |
| Electroencephalography (EEG) with recording of event-related potentials (ERP) using auditory P3 ‘oddball paradigm’ | P3 amplitude | Tanaka*,* 2000 [156] | 29 |  |  |  | r = 0.40 |  |  |
|  | Mean amplitude after rare stimuli | Tanaka*,* 2000 [156] | 29 |  |  |  | r = 0.38 |  |  |
|  | EEG total power | Tanaka*,* 2000 [156] | 27 |  |  |  | r = 0.43 |  |  |
|  | P3 latency | Tanaka*,* 2000 [156] | 29 |  |  |  | r = -0.43 |  |  |
|  | N2 latency | Broussolle, 1999 [140] | 27 |  |  | No significance difference across studied groups † |  |  |  |
|  | P3 latency | Broussolle, 1999 [140] | 27 |  |  | No significance difference across studied groups † |  |  |  |
| Transcranial magnetic stimulation | Threshold intensity (TI) | Bhatia*,* 2003 [157] | 19 |  |  | No significant difference (H&Y 1-1.5 versus 2) |  |  |  |
|  | Cortical latency (CL) | Bhatia*,* 2003 [157] | 19 |  |  | No significant difference (H&Y 1-1.5 versus 2) |  |  |  |
|  | Central conduction time (CCT) | Bhatia*,* 2003 [157] | 19 |  |  | No significant difference (H&Y 1-1.5 versus 2) |  |  |  |

| **Electrophysiological** | |  |  | **Association of electrophysiological feature measured with:** | | | | | |
| --- | --- | --- | --- | --- | --- | --- | --- | --- | --- |
| **Modality** | **Feature measured** | **Reference**  **(first author, year)** | **n** | **Total UPDRS** | **UPDRS**  **(III)** | **H&Y** | **MMSE** | **WDS** | **SCOPA-AUT** |
| Electromyography (EMG) | Biceps burst duration variability | Robichaud*,* 2009 [158] | 30 |  | r = 0.41 |  |  |  |  |
|  | Percentage of short 1^st^ agonist burst durations | Robichaud*,* 2009 [158] | 30 |  | r = 0.66 |  |  |  |  |
|  | Laryngeal relocation time (0-2 interval) | Ertekin*,* 2002 [159] | 58 |  |  | NSA |  | NSA |  |
|  | Duration of the submental EMG  (A-C interval) | Ertekin*,* 2002 [159] | 58 |  |  | r = 0.29 |  | NSA |  |
|  | Triggering time of swallowing reflex  (A-0 time) | Ertekin*,* 2002 [159] | 58 |  |  | NSA |  | NSA |  |
| Muscle sympathetic nerve activity (MSNA) | MSNA of peroneal nerve fascicles in right popliteal fossa | Shindo, 2005 [82] | 14 |  |  | r = -0.86 |  |  |  |
| Sympathetic skin response (SSR) | SSR of palm | Papapetropoulos*,* 2006 [160] | 22 |  |  |  |  |  | No difference between those with severe and less severe scores |
|  | SSR of sole | Papapetropoulos*,* 2006 [160] | 22 |  |  |  |  |  | No difference between those with severe and less severe scores |

| **Electrophysiological** | |  |  | **Association of electrophysiological feature measured with:** | | | | | |
| --- | --- | --- | --- | --- | --- | --- | --- | --- | --- |
| **Modality** | **Feature measured** | **Reference**  **(first author, year)** | **n** | **Total UPDRS** | **UPDRS**  **(III)** | **H&Y** | **MMSE** | **WDS** | **SCOPA-AUT** |
| Electrocardiography  (ECG) | R-R interval variation (RRIV) when breathing normally | Papapetropoulos*,* 2006 [160] | 22 |  |  |  |  |  | No difference between those with severe and less severe scores |
|  | R-R interval variation (RRIV) when breathing deeply | Papapetropoulos*,* 2006 [160] | 22 |  |  |  |  |  | No difference between those with severe and less severe scores |
| 24 hour ambulatory Electrocardiography  (ECG) | RR interval (RRI) | Haapaniemi*,* 2001 [161] | 54 | NSA | NSA | NSA |  |  |  |
|  | Standard deviation of all RRIs (SDNN) | Haapaniemi*,* 2001 [161] | 54 | NSA | NSA | NSA |  |  |  |
|  | Heart rate variability: very low frequency (VLF) power spectrum densities | Haapaniemi*,* 2001 [161] | 54 | r = -0.34 | r = -0.38 | NSA |  |  |  |
|  | Heart rate variability: low frequency (LF) power spectrum densities | Haapaniemi*,* 2001 [161] | 54 | r = -0.34 | r = -0.37 | NSA |  |  |  |
|  | Heart rate variability: high frequency (HF) power spectrum densities | Haapaniemi*,* 2001 [161] | 54 | NSA | NSA | NSA |  |  |  |

| **Electrophysiological** | |  |  | **Association of electrophysiological feature measured with:** | | | | | |
| --- | --- | --- | --- | --- | --- | --- | --- | --- | --- |
| **Modality** | **Feature measured** | **Reference**  **(first author, year)** | **n** | **Total UPDRS** | **UPDRS**  **(III)** | **H&Y** | **MMSE** | **WDS** | **SCOPA-AUT** |
| 24 hour ambulatory Electrocardiography  (ECG) [continued] | Instantaneous beat to beat RRI variability (SD1) | Haapaniemi*,* 2001 [161] | 54 | NSA | NSA | NSA |  |  |  |
|  | Long term continuous RRI variability (SD2) | Haapaniemi*,* 2001 [161] | 54 | NSA | NSA | NSA |  |  |  |
|  | Slope of power law relation (slope of HRV) | Haapaniemi*,* 2001 [161] | 54 | r = -0.30 | r = -0.29 | NSA |  |  |  |

**Key**

† Patients were assigned to three groups:

(1) Untreated patients with Parkinson’s disease, diagnosis made in previous six months, H&Y 1-2 (n=8);

(2) Patents with Parkinson’s disease treated with levodopa and a dopa decarboxylase inhibitor for at least one year,

signs of motor fluctuations and mild to moderate dyskinesias, H&Y 2-3 (n=11);

(3) Marked on-off motor fluctuations and moderate to pronounced dyskinesias, H&Y 4-5.

**Additional file 3 *10: Other***

**Associations between miscellaneous putative biomarkers and clinical measures of disease severity, in cross-sectional studies included in the systemic review of biomarkers for disease progression in Parkinson’s disease**

|  |  |  |  | **Association of concentration of substance measured with:** | | |
| --- | --- | --- | --- | --- | --- | --- |
| **Modality** | **Feature measured** | **Reference**  **(first author, year)** | **n** | **UPDRS**  **(III)** | **UPDRS**  **(II)** | **H&Y** |
| Hair sample | Calcium | Forte*,* 2005 [162] | 81 |  |  | NSA |
|  | Copper | Forte*,* 2005 [162] | 81 |  |  | NSA |
|  | Iron | Forte*,* 2005 [162] | 81 |  |  | NSA |
|  | Magnesium | Forte*,* 2005 [162] | 81 |  |  | NSA |
|  | Silicon | Forte*,* 2005 [162] | 81 |  |  | NSA |
|  | Zinc | Forte*,* 2005 [162] | 81 |  |  | NSA |
| Modified barium swallow with video fluoroscopy | Oral transit time (OTT) | Monte*,* 2005 [163] | 27 | NSA | NSA | NSA |
|  | Pharyngeal transit time (PTT) | Monte*,* 2005 [163] | 27 | NSA | NSA | NSA |
|  | Oropharyngeal swallow efficiency (OPSE) | Monte*,* 2005 [163] | 27 | NSA | NSA | NSA |
|  | Liquid and solid pharyngeal residue | Monte*,* 2005 [163] | 27 | NSA | NSA | NSA |

**Overall key**

Superscript numbers correspond to the list of references

**Correlations**

In many cases it was unclear whether a given correlation coefficient was a Pearson’s or Spearman’s correlation coefficient. Therefore, for simplicity all correlation coefficients are simply denoted as ‘r’

NSA No significant association

POS Positive association, correlation coefficient not stated

NEG Negative association, correlation coefficient not stated

All other associations where r value is stated are significant (P < 0.05)

**Clinical rating scales**

Blessed IMC Blessed Dementia Information-Memory-Concentration test [164]

Contralateral UPDRS (III) Motor component of the unified Parkinson’s disease rating scale for the side of the body contralateral to

the side in which an area of the brain was measured

CURS Columbia University Rating Scale [165]

DRS Dementia Rating Scale [166]

GDS Global Deterioration Scale [167]

H&Y Hoehn and Yahr staging scale [168]

HDSR Hasegawa’s Dementia Scale Revised [169]

Ipsi. UPRS (III) Motor component of the Unified Parkinson’s Disease Rating Scale for the side of body ipsilateral to

the side in which an area of the brain was measured

Lateralized UPDRS (III) Motor component of the Unified Parkinson’s Disease Rating Scale for either side of the body alone,

for the most severely affected side, or for the least severely affected side

MMSE Mini-Mental State Examination [170]

NUDS Northwestern University Disability Scale [171]

S&E Schwab and England activities of daily living (ADL) scale [172]

SCOPA-AUT Questionnaire assessment of autonomic symptoms in Parkinson’s disease [173]

TCS Total Clinical Score. A semi-quantitative five-point scale for bradykinesia, tremor and rigidity

in all four limbs (0=absent and 4=very marked)

Total CAMCOG The cognitive and self-contained part of the Cambridge Examination for Mental disorders of the Elderly [174]

Total UPDRS Total score derived from the Unified Parkinson’s Disease Rating Scale [175]

UPDRS (I) Mentation component of the Unified Parkinson’s Disease Rating Scale

UPDRS (II) Activities of daily living component of the Unified Parkinson’s Disease Rating Scale

UPDRS (III) Motor component of the Unified Parkinson’s Disease Rating Scale

WDS Webster’s Disability scale [176]

**Areas of the brain**

CN Caudate nucleus

FWM Frontal white matter

GP Globus pallidus

PU Putamen

SN Substantia nigra

ST Striatum

**Other**

Ant Anterior

Post Posterior

APC Annual percentage change

rCBF Regional cerebral blood flow

**References**

1. Shi M, Zabetian CP, Hancock AM, Ginghina C, Hong Z, Yearout D, [Chung KA](http://www.ncbi.nlm.nih.gov/pubmed?term=Chung%20KA%5BAuthor%5D&cauthor=true&cauthor_uid=20540987), [Quinn JF](http://www.ncbi.nlm.nih.gov/pubmed?term=Quinn%20JF%5BAuthor%5D&cauthor=true&cauthor_uid=20540987), [Peskind ER](http://www.ncbi.nlm.nih.gov/pubmed?term=Peskind%20ER%5BAuthor%5D&cauthor=true&cauthor_uid=20540987), [Galasko D](http://www.ncbi.nlm.nih.gov/pubmed?term=Galasko%20D%5BAuthor%5D&cauthor=true&cauthor_uid=20540987), [Jankovic J](http://www.ncbi.nlm.nih.gov/pubmed?term=Jankovic%20J%5BAuthor%5D&cauthor=true&cauthor_uid=20540987), [Leverenz JB](http://www.ncbi.nlm.nih.gov/pubmed?term=Leverenz%20JB%5BAuthor%5D&cauthor=true&cauthor_uid=20540987), [Zhang J](http://www.ncbi.nlm.nih.gov/pubmed?term=Zhang%20J%5BAuthor%5D&cauthor=true&cauthor_uid=20540987): **Significance and confounders of peripheral DJ-1 and alpha-synuclein in Parkinson's disease.** *Neurosci Lett* 2010, **480(1):**78-82.

2. Waragai M, Nakai M, Wei J, Fujita M, Mizuno H, Ho G, [Masliah E](http://www.ncbi.nlm.nih.gov/pubmed?term=Masliah%20E%5BAuthor%5D&cauthor=true&cauthor_uid=17720313), [Akatsu H](http://www.ncbi.nlm.nih.gov/pubmed?term=Akatsu%20H%5BAuthor%5D&cauthor=true&cauthor_uid=17720313), [Yokochi F](http://www.ncbi.nlm.nih.gov/pubmed?term=Yokochi%20F%5BAuthor%5D&cauthor=true&cauthor_uid=17720313), [Hashimoto M](http://www.ncbi.nlm.nih.gov/pubmed?term=Hashimoto%20M%5BAuthor%5D&cauthor=true&cauthor_uid=17720313): **Plasma levels of DJ-1 as a possible marker for progression of sporadic Parkinson's disease.** *Neurosci Lett* 2007, **425(1):**18-22.

3. Eldrup E, Mogensen P, Jacobsen J, Pakkenberg H, Christensen NJ: **CSF and plasma concentrations of free norepinephrine, dopamine, 3,4-dihydroxyphenylacetic acid (DOPAC), 3,4-dihydroxyphenylalanine (DOPA), and epinephrine in Parkinson's disease.** *Acta Neurol Scand* 1995, **92(2):**116-121.

4. Chen CM, Liu JL, Wu YR, Chen YC, Cheng HS, Cheng ML, Chiu DT: **Increased oxidative damage in peripheral blood correlates with severity of Parkinson's disease.** *Neurobiol Dis* 2009, **33(3):**429-435.

5. Ilic TV, Jovanovic M, Jovicic A, Tomovic M: **Oxidative stress indicators are elevated in de novo Parkinson's disease patients.** *Funct Neurol* 1999, **14(3):**141-147.

6. Seet RC, Lee CY, Lim EC, Tan JJ, Quek AM, Chong WL, [Looi WF](http://www.ncbi.nlm.nih.gov/pubmed?term=Looi%20WF%5BAuthor%5D&cauthor=true&cauthor_uid=19969070), [Huang SH](http://www.ncbi.nlm.nih.gov/pubmed?term=Huang%20SH%5BAuthor%5D&cauthor=true&cauthor_uid=19969070), [Wang H](http://www.ncbi.nlm.nih.gov/pubmed?term=Wang%20H%5BAuthor%5D&cauthor=true&cauthor_uid=19969070), [Chan YH](http://www.ncbi.nlm.nih.gov/pubmed?term=Chan%20YH%5BAuthor%5D&cauthor=true&cauthor_uid=19969070), [Halliwell B](http://www.ncbi.nlm.nih.gov/pubmed?term=Halliwell%20B%5BAuthor%5D&cauthor=true&cauthor_uid=19969070): **Oxidative damage in Parkinson disease: Measurement using accurate biomarkers.** *Free Radic Biol Med* 2010, **48(4):**560-566.

7. Song IU, Kim JS, Chung SW, Lee KS: **Is there an association between the level of high-sensitivity C-reactive protein and idiopathic Parkinson's disease? A comparison of Parkinson's disease patients, disease controls and healthy individuals.** *Eur Neurol*  2009, **62(2):**99-104.

8. Ihara Y, Chuda M, Kuroda S, Hayabara T: **Hydroxyl radical and superoxide dismutase in blood of patients with Parkinson's disease: relationship to clinical data.** *J Neurol Sci* 1999, **170(2):**90-95.

9. Irizarry MC, Yao Y, Hyman BT, Growdon JH, Pratico D: **Plasma F2A isoprostane levels in Alzheimer's and Parkinson's disease.** *Neurodegener Dis* 2007, **4(6):**403-405.

10. Connolly J, Siderowf A, Clark CM, Mu D, Pratico D: **F2 isoprostane levels in plasma and urine do not support increased lipid peroxidation in cognitively impaired Parkinson disease patients.** *Cogn Behav Neurol* 2008, **21(2):**83-86.

11. Sanyal J, Bandyopadhyay SK, Banerjee TK, Mukherjee SC, Chakraborty DP, Ray BC, Rao VR: **Plasma levels of lipid peroxides in patients with Parkinson's disease.** *Eur Rev Med Pharmacol Sci* 2009, **13(2):**129-132.

12. Molina JA, Jimenez-Jimenez FJ, Fernandez-Calle P, Lalinde L, Tenias JM, Pondal M, [Vazquez A](http://www.ncbi.nlm.nih.gov/pubmed?term=Vazquez%20A%5BAuthor%5D&cauthor=true&cauthor_uid=1641180), [Codoceo R](http://www.ncbi.nlm.nih.gov/pubmed?term=Codoceo%20R%5BAuthor%5D&cauthor=true&cauthor_uid=1641180): **Serum lipid peroxides in patients with Parkinson's disease.** *Neurosci Lett* 1992, **136(2):**137-140.

13. Sanyal J, Sarkar BN, Banerjee TK, Mukherjee SC, Ray BC, Rao VR: **Plasma level of nitrates in patients with parkinson's disease in west bengal.** *Neurology Asia* 2010, **15(1):**55-59.

14. Molina JA, Jimenez-Jimenez FJ, Navarro JA, Ruiz E, Arenas J, Cabrera-Valdivia F, Vazquez A, Fernandez-Calle P, Ayuso-Peralta L, Rabasa M, Bermejo F: **Plasma levels of nitrates in patients with Parkinson's disease.** *J Neurol Sci* 1994, **127(1):**87-89.

15. Molina JA, Jimenez-Jimenez FJ, Navarro JA, Vargas C, Gomez P, Benito-Leon J, [Ortí-Pareja M](http://www.ncbi.nlm.nih.gov/pubmed?term=Ort%C3%AD-Pareja%20M%5BAuthor%5D&cauthor=true&cauthor_uid=8741130), [Cisneros E](http://www.ncbi.nlm.nih.gov/pubmed?term=Cisneros%20E%5BAuthor%5D&cauthor=true&cauthor_uid=8741130), [Arenas J](http://www.ncbi.nlm.nih.gov/pubmed?term=Arenas%20J%5BAuthor%5D&cauthor=true&cauthor_uid=8741130): **Cerebrospinal fluid nitrate levels in patients with Parkinson's disease.** *Acta Neurol Scand* 1996, **93(2-3):**123-126.

16. Benecke R, Strumper P, Weiss H: **Electron transfer complexes I and IV of platelets are abnormal in Parkinson's disease but normal in Parkinson-plus syndromes.** *Brain* 1993, **116(6):**1451-1463.

17. Gotz ME, Gerstner A, Harth R, Dirr A, Janetzky B, Kuhn W, [Riederer P](http://www.ncbi.nlm.nih.gov/pubmed?term=Riederer%20P%5BAuthor%5D&cauthor=true&cauthor_uid=10809402), [Gerlach M](http://www.ncbi.nlm.nih.gov/pubmed?term=Gerlach%20M%5BAuthor%5D&cauthor=true&cauthor_uid=10809402): **Altered redox state of platelet coenzyme Q10 in Parkinson's disease.** *J Neural Transm* 2000, **107(1):**41-48.

18. Husain M, Shukla R, Dikshit M, Maheshwari PK, Nag D, Srimal RC, Seth PK, Khanna VK: **Altered platelet monoamine oxidase-B activity in idiopathic Parkinson's disease.** *Neurochem Res* 2009, **34(8):**1427-1432.

19. Bongioanni P, Mondino C, Boccardi B, Borgna M, Castagna M: **Monoamine oxidase molecular activity in platelets of parkinsonian and demented patients.** *Neurodegeneration* 1996, **5(4):**351-357.

20. Kuiper MA, Konings CH, Bergmans PL, Wolters EC: **Whole blood monoamine oxidase activity in Parkinson's disease and multiple system atrophy patients.** *J Neurol Sci* 1993, **117(1-2):**41-45.

21. D'Andrea G, Nordera G, Pizzolato G, Bolner A, Colavito D, Flaibani R, Leon A: **Trace amine metabolism in Parkinson's disease: low circulating levels of octopamine in early disease stages.** *Neurosci Lett* 2010, **469(3):**348-351.

22. Jimenez-Jimenez FJ, Rubio JC, Molina JA, Martin MA, Campos Y, Benito-Leon J, [Ortí-Pareja M](http://www.ncbi.nlm.nih.gov/pubmed?term=Ort%C3%AD-Pareja%20M%5BAuthor%5D&cauthor=true&cauthor_uid=9094047), [Gasalla T](http://www.ncbi.nlm.nih.gov/pubmed?term=Gasalla%20T%5BAuthor%5D&cauthor=true&cauthor_uid=9094047), [Arenas J](http://www.ncbi.nlm.nih.gov/pubmed?term=Arenas%20J%5BAuthor%5D&cauthor=true&cauthor_uid=9094047): **Cerebrospinal fluid carnitine levels in patients with Parkinson's disease.** *J Neurol Sci* 1997, **145(2):**183-185.

23. Jimenez-Jimenez FJ, Molina JA, Vargas C, Gomez P, Navarro JA, Benito-Leon J, [Ortí-Pareja M](http://www.ncbi.nlm.nih.gov/pubmed?term=Ort%C3%AD-Pareja%20M%5BAuthor%5D&cauthor=true&cauthor_uid=8880690), [Gasalla T](http://www.ncbi.nlm.nih.gov/pubmed?term=Gasalla%20T%5BAuthor%5D&cauthor=true&cauthor_uid=8880690), [Cisneros E](http://www.ncbi.nlm.nih.gov/pubmed?term=Cisneros%20E%5BAuthor%5D&cauthor=true&cauthor_uid=8880690), [Arenas J](http://www.ncbi.nlm.nih.gov/pubmed?term=Arenas%20J%5BAuthor%5D&cauthor=true&cauthor_uid=8880690): **Neurotransmitter amino acids in cerebrospinal fluid of patients with Parkinson's disease.** *J Neurol Sci* 1996, **141(1-2):**39-44.

24. Camicioli RM, Bouchard TP, Somerville MJ: **Homocysteine is not associated with global motor or cognitive measures in nondemented older Parkinson's disease patients.** *Mov Disord* 2009, **24(2):**176-182.

25. Hassin-Baer S, Cohen O, Vakil E, Sela BA, Nitsan Z, Schwartz R, Chapman J Tanne D: **Plasma homocysteine levels and Parkinson disease: disease progression, carotid intima-media thickness and neuropsychiatric complications.** *Clin Neuropharmacol* 2006, **29(6):**305-311.

26. O'Suilleabhain PE, Sung V, Hernandez C, Lacritz L, Dewey RB, Jr., Bottiglieri T, Diaz-Arrastia R: **Elevated plasma homocysteine level in patients with Parkinson disease: motor, affective, and cognitive associations.** *Arch Neurol* 2004, **61(6):**865-868.

27. Rodriguez-Oroz MC, Lage PM, Sanchez-Mut J, Lamet I, Pagonabarraga J, Toledo JB, [García-Garcia D](http://www.ncbi.nlm.nih.gov/pubmed?term=Garc%C3%ADa-Garcia%20D%5BAuthor%5D&cauthor=true&cauthor_uid=19452554), [Clavero P](http://www.ncbi.nlm.nih.gov/pubmed?term=Clavero%20P%5BAuthor%5D&cauthor=true&cauthor_uid=19452554), [Samaranch L](http://www.ncbi.nlm.nih.gov/pubmed?term=Samaranch%20L%5BAuthor%5D&cauthor=true&cauthor_uid=19452554), [Irurzun C](http://www.ncbi.nlm.nih.gov/pubmed?term=Irurzun%20C%5BAuthor%5D&cauthor=true&cauthor_uid=19452554), [Matsubara JM](http://www.ncbi.nlm.nih.gov/pubmed?term=Matsubara%20JM%5BAuthor%5D&cauthor=true&cauthor_uid=19452554), [Irigoien J](http://www.ncbi.nlm.nih.gov/pubmed?term=Irigoien%20J%5BAuthor%5D&cauthor=true&cauthor_uid=19452554), [Bescos E](http://www.ncbi.nlm.nih.gov/pubmed?term=Bescos%20E%5BAuthor%5D&cauthor=true&cauthor_uid=19452554), [Kulisevsky J](http://www.ncbi.nlm.nih.gov/pubmed?term=Kulisevsky%20J%5BAuthor%5D&cauthor=true&cauthor_uid=19452554), [Pérez-Tur J](http://www.ncbi.nlm.nih.gov/pubmed?term=P%C3%A9rez-Tur%20J%5BAuthor%5D&cauthor=true&cauthor_uid=19452554), [Obeso JA](http://www.ncbi.nlm.nih.gov/pubmed?term=Obeso%20JA%5BAuthor%5D&cauthor=true&cauthor_uid=19452554): **Homocysteine and cognitive impairment in Parkinson's disease: a biochemical, neuroimaging, and genetic study.** *Mov Disord* 2009, **24(10):**1437-1444.

28. Irizarry MC, Gurol ME, Raju S, Diaz-Arrastia R, Locascio JJ, Tennis M, [Hyman BT](http://www.ncbi.nlm.nih.gov/pubmed?term=Hyman%20BT%5BAuthor%5D&cauthor=true&cauthor_uid=16275827), [Growdon JH](http://www.ncbi.nlm.nih.gov/pubmed?term=Growdon%20JH%5BAuthor%5D&cauthor=true&cauthor_uid=16275827), [Greenberg SM](http://www.ncbi.nlm.nih.gov/pubmed?term=Greenberg%20SM%5BAuthor%5D&cauthor=true&cauthor_uid=16275827), [Bottiglieri T](http://www.ncbi.nlm.nih.gov/pubmed?term=Bottiglieri%20T%5BAuthor%5D&cauthor=true&cauthor_uid=16275827): **Association of homocysteine with plasma amyloid beta protein in aging and neurodegenerative disease.** *Neurology* 2005, **65(9):**1402-1408.

29. Andreadou E, Nikolaou C, Gournaras F, Rentzos M, Boufidou F, Tsoutsou A, [Zournas C](http://www.ncbi.nlm.nih.gov/pubmed?term=Zournas%20C%5BAuthor%5D&cauthor=true&cauthor_uid=19632030), [Zissimopoulos V](http://www.ncbi.nlm.nih.gov/pubmed?term=Zissimopoulos%20V%5BAuthor%5D&cauthor=true&cauthor_uid=19632030), [Vassilopoulos D](http://www.ncbi.nlm.nih.gov/pubmed?term=Vassilopoulos%20D%5BAuthor%5D&cauthor=true&cauthor_uid=19632030): **Serum uric acid levels in patients with Parkinson's disease: their relationship to treatment and disease duration.** *Clin Neurol Neurosurg* 2009, **111(9):**724-728.

30. Hegde ML, Shanmugavelu P, Vengamma B, Rao TS, Menon RB, Rao RV, Rao KS: **Serum trace element levels and the complexity of inter-element relations in patients with Parkinson's disease.** *J Trace Elem Med Biol* 2004, **18(2):**163-171.

31. Arnal N, Cristalli DO, de Alaniz MJ, Marra CA: **Clinical utility of copper, ceruloplasmin, and metallothionein plasma determinations in human neurodegenerative patients and their first-degree relatives.** *Brain Res* 2010, **1319:**118-130.

32. Jimenez-Jimenez FJ, Fernandez-Calle P, Martinez-Vanaclocha M, Herrero E, Molina JA, Vazquez A, *Codoceo R:* **Serum levels of zinc and copper in patients with Parkinson's disease.** *J Neurol Sci* 1992, **112(1-2):**30-33.

33. Jimenez-Jimenez FJ, Molina JA, Aguilar MV, Meseguer I, Mateos-Vega CJ, Gonzalez-Munoz MJ, [de Bustos F](http://www.ncbi.nlm.nih.gov/pubmed?term=de%20Bustos%20F%5BAuthor%5D&cauthor=true&cauthor_uid=9720977), [Martínez-Salio A](http://www.ncbi.nlm.nih.gov/pubmed?term=Mart%C3%ADnez-Salio%20A%5BAuthor%5D&cauthor=true&cauthor_uid=9720977), [Ortí-Pareja M](http://www.ncbi.nlm.nih.gov/pubmed?term=Ort%C3%AD-Pareja%20M%5BAuthor%5D&cauthor=true&cauthor_uid=9720977), [Zurdo M](http://www.ncbi.nlm.nih.gov/pubmed?term=Zurdo%20M%5BAuthor%5D&cauthor=true&cauthor_uid=9720977), [Martínez-Para MC](http://www.ncbi.nlm.nih.gov/pubmed?term=Mart%C3%ADnez-Para%20MC%5BAuthor%5D&cauthor=true&cauthor_uid=9720977): **Cerebrospinal fluid levels of transition metals in patients with Parkinson's disease.** *J Neural Transm* 1998, **105(4-5):**497-505.

34. Torsdottir G, Sveinbjornsdottir S, Kristinsson J, Snaedal J, Johannesson T: **Ceruloplasmin and superoxide dismutase (SOD1) in Parkinson's disease: a follow-up study.** *J Neurol Sci* 2006, **241(1-2):**53-58.

35. Cabrera-Valdivia F, Jimenez-Jimenez FJ, Molina JA, Fernandez-Calle P, Vazquez A, Canizares-Liebana F, [Larumbe-Lobalde S](http://www.ncbi.nlm.nih.gov/pubmed?term=Larumbe-Lobalde%20S%5BAuthor%5D&cauthor=true&cauthor_uid=7964893), [Ayuso-Peralta L](http://www.ncbi.nlm.nih.gov/pubmed?term=Ayuso-Peralta%20L%5BAuthor%5D&cauthor=true&cauthor_uid=7964893), [Rabasa M](http://www.ncbi.nlm.nih.gov/pubmed?term=Rabasa%20M%5BAuthor%5D&cauthor=true&cauthor_uid=7964893), [Codoceo R](http://www.ncbi.nlm.nih.gov/pubmed?term=Codoceo%20R%5BAuthor%5D&cauthor=true&cauthor_uid=7964893): **Peripheral iron metabolism in patients with Parkinson's disease.** *J Neurol Sci* 1994, **125(1):**82-86.

36. Aguilar MV, Jimenez-Jimenez FJ, Molina JA, Meseguer I, Mateos-Vega CJ, Gonzalez-Munoz MJ, [de Bustos F](http://www.ncbi.nlm.nih.gov/pubmed?term=de%20Bustos%20F%5BAuthor%5D&cauthor=true&cauthor_uid=9928893), [Gómez-Escalonilla C](http://www.ncbi.nlm.nih.gov/pubmed?term=G%C3%B3mez-Escalonilla%20C%5BAuthor%5D&cauthor=true&cauthor_uid=9928893), [Ort-Pareja M](http://www.ncbi.nlm.nih.gov/pubmed?term=Ort-Pareja%20M%5BAuthor%5D&cauthor=true&cauthor_uid=9928893), [Zurdo M](http://www.ncbi.nlm.nih.gov/pubmed?term=Zurdo%20M%5BAuthor%5D&cauthor=true&cauthor_uid=9928893), [Martínez-Para MC](http://www.ncbi.nlm.nih.gov/pubmed?term=Mart%C3%ADnez-Para%20MC%5BAuthor%5D&cauthor=true&cauthor_uid=9928893): **Cerebrospinal fluid selenium and chromium levels in patients with Parkinson's disease.** *J Neural Transm* 1998, **105(10-12):**1245-1251.

37. Jimenez-Jimenez FJ, Molina JA, Fernandez-Calle P, Vazquez A, Pondal M, del ST, [Gómez-Pastor A](http://www.ncbi.nlm.nih.gov/pubmed?term=G%C3%B3mez-Pastor%20A%5BAuthor%5D&cauthor=true&cauthor_uid=1403000), [Codoceo R](http://www.ncbi.nlm.nih.gov/pubmed?term=Codoceo%20R%5BAuthor%5D&cauthor=true&cauthor_uid=1403000): **Serum levels of vitamin A in Parkinson's disease.** *J Neurol Sci* 1992, **111(1):**73-76.

38. King D, Playfer JR, Roberts NB: **Concentrations of vitamins A, C and E in elderly patients with Parkinson's disease.** *Postgrad Med J* 1992, **68(802):**634-637.

39. Jimenez-Jimenez FJ, Molina JA, Fernandez-Calle P, Vazquez A, Cabrera-Valdivia F, Catalan MJ, [García-Albea E](http://www.ncbi.nlm.nih.gov/pubmed?term=Garc%C3%ADa-Albea%20E%5BAuthor%5D&cauthor=true&cauthor_uid=8233018), [Bermejo F](http://www.ncbi.nlm.nih.gov/pubmed?term=Bermejo%20F%5BAuthor%5D&cauthor=true&cauthor_uid=8233018), [Codoceo R](http://www.ncbi.nlm.nih.gov/pubmed?term=Codoceo%20R%5BAuthor%5D&cauthor=true&cauthor_uid=8233018): **Serum levels of beta-carotene and other carotenoids in Parkinson's disease.** *Neurosci Lett* 1993, **157(1):**103-106.

40. Fernandez-Calle P, Jimenez-Jimenez FJ, Molina JA, Cabrera-Valdivia F, Vazquez A, Garcia UD, [Bermejo F](http://www.ncbi.nlm.nih.gov/pubmed?term=Bermejo%20F%5BAuthor%5D&cauthor=true&cauthor_uid=8229047), [Cruz Matallana M](http://www.ncbi.nlm.nih.gov/pubmed?term=Cruz%20Matallana%20M%5BAuthor%5D&cauthor=true&cauthor_uid=8229047), [Codoceo R](http://www.ncbi.nlm.nih.gov/pubmed?term=Codoceo%20R%5BAuthor%5D&cauthor=true&cauthor_uid=8229047): **Serum levels of ascorbic acid (vitamin C) in patients with Parkinson's disease.** *J Neurol Sci* 1993, **118(1):**25-28.

41. Fernandez-Calle P, Molina JA, Jimenez-Jimenez FJ, Vazquez A, Pondal M, Garcia-Ruiz PJ, [Urra DG](http://www.ncbi.nlm.nih.gov/pubmed?term=Urra%20DG%5BAuthor%5D&cauthor=true&cauthor_uid=1579230), [Domingo J](http://www.ncbi.nlm.nih.gov/pubmed?term=Domingo%20J%5BAuthor%5D&cauthor=true&cauthor_uid=1579230), [Codoceo R](http://www.ncbi.nlm.nih.gov/pubmed?term=Codoceo%20R%5BAuthor%5D&cauthor=true&cauthor_uid=1579230): **Serum levels of alpha-tocopherol (vitamin E) in Parkinson's disease.** *Neurology* 1992, **42(5):**1064-1066.

42. Molina JA, De BF, Jimenez-Jimenez FJ, Benito-Leon J, Orti-Pareja M, Gasalla T, [Tallón-Barranco A](http://www.ncbi.nlm.nih.gov/pubmed?term=Tall%C3%B3n-Barranco%20A%5BAuthor%5D&cauthor=true&cauthor_uid=9503274), [Navarro JA](http://www.ncbi.nlm.nih.gov/pubmed?term=Navarro%20JA%5BAuthor%5D&cauthor=true&cauthor_uid=9503274), [Arenas J](http://www.ncbi.nlm.nih.gov/pubmed?term=Arenas%20J%5BAuthor%5D&cauthor=true&cauthor_uid=9503274), [Enríquez-de-Salamanca R](http://www.ncbi.nlm.nih.gov/pubmed?term=Enr%C3%ADquez-de-Salamanca%20R%5BAuthor%5D&cauthor=true&cauthor_uid=9503274): **Cerebrospinal fluid levels of alpha-tocopherol (vitamin E) in Parkinson's disease.** *J Neural Transm* 1997, **104(11-12):**1287-1293.

43. Jimenez-Jimenez FJ, Molina JA, De BF, Garcia-Redondo A, Gomez-Escalonilla C, Martinez-Salio A, [Berbel A](http://www.ncbi.nlm.nih.gov/pubmed?term=Berbel%20A%5BAuthor%5D&cauthor=true&cauthor_uid=10847558), [Camacho A](http://www.ncbi.nlm.nih.gov/pubmed?term=Camacho%20A%5BAuthor%5D&cauthor=true&cauthor_uid=10847558), [Zurdo M](http://www.ncbi.nlm.nih.gov/pubmed?term=Zurdo%20M%5BAuthor%5D&cauthor=true&cauthor_uid=10847558), [Barcenilla B](http://www.ncbi.nlm.nih.gov/pubmed?term=Barcenilla%20B%5BAuthor%5D&cauthor=true&cauthor_uid=10847558), [Enríquez de Salamanca R](http://www.ncbi.nlm.nih.gov/pubmed?term=Enr%C3%ADquez%20de%20Salamanca%20R%5BAuthor%5D&cauthor=true&cauthor_uid=10847558), [Arenas J](http://www.ncbi.nlm.nih.gov/pubmed?term=Arenas%20J%5BAuthor%5D&cauthor=true&cauthor_uid=10847558): **Serum levels of coenzyme Q10 in patients with Parkinson's disease.** *J Neural Transm* 2000, **107(2):**177-181.

44. Hofmann KW, Schuh AF, Saute J, Townsend R, Fricke D, Leke R, [Souza DO](http://www.ncbi.nlm.nih.gov/pubmed?term=Souza%20DO%5BAuthor%5D&cauthor=true&cauthor_uid=19214748), [Portela LV](http://www.ncbi.nlm.nih.gov/pubmed?term=Portela%20LV%5BAuthor%5D&cauthor=true&cauthor_uid=19214748), [Chaves ML](http://www.ncbi.nlm.nih.gov/pubmed?term=Chaves%20ML%5BAuthor%5D&cauthor=true&cauthor_uid=19214748), [Rieder CR](http://www.ncbi.nlm.nih.gov/pubmed?term=Rieder%20CR%5BAuthor%5D&cauthor=true&cauthor_uid=19214748): **Interleukin-6 serum levels in patients with Parkinson's disease.** *Neurochem Res* 2009, **34(8):**1401-1404.

45. Rentzos M, Nikolaou C, Andreadou E, Paraskevas GP, Rombos A, Zoga M, [Tsoutsou A](http://www.ncbi.nlm.nih.gov/pubmed?term=Tsoutsou%20A%5BAuthor%5D&cauthor=true&cauthor_uid=18976327), [Boufidou F](http://www.ncbi.nlm.nih.gov/pubmed?term=Boufidou%20F%5BAuthor%5D&cauthor=true&cauthor_uid=18976327), [Kapaki E](http://www.ncbi.nlm.nih.gov/pubmed?term=Kapaki%20E%5BAuthor%5D&cauthor=true&cauthor_uid=18976327), [Vassilopoulos D](http://www.ncbi.nlm.nih.gov/pubmed?term=Vassilopoulos%20D%5BAuthor%5D&cauthor=true&cauthor_uid=18976327): **Circulating interleukin-10 and interleukin-12 in Parkinson's disease.** *Acta Neurol Scand* 2009, **119(5):**332-337.

46. Rentzos M, Nikolaou C, Andreadou E, Paraskevas GP, Rombos A, Zoga M, [Tsoutsou A](http://www.ncbi.nlm.nih.gov/pubmed?term=Tsoutsou%20A%5BAuthor%5D&cauthor=true&cauthor_uid=17986095), [Boufidou F](http://www.ncbi.nlm.nih.gov/pubmed?term=Boufidou%20F%5BAuthor%5D&cauthor=true&cauthor_uid=17986095), [Kapaki E](http://www.ncbi.nlm.nih.gov/pubmed?term=Kapaki%20E%5BAuthor%5D&cauthor=true&cauthor_uid=17986095), [Vassilopoulos D](http://www.ncbi.nlm.nih.gov/pubmed?term=Vassilopoulos%20D%5BAuthor%5D&cauthor=true&cauthor_uid=17986095): **Circulating interleukin-15 and RANTES chemokine in Parkinson's disease.** *Acta Neurol Scand* 2007, **116(6):**374-379.

47. Contin M, Riva R, Martinelli P, Cortelli P, Albani F, Baruzzi A: **A levodopa kinetic-dynamic study of the rate of progression in Parkinson's disease.** *Neurology* 1998, **51(4):**1075-1080.

48. Takubo H, Shimoda-Matsubayashi S, Mizuno Y: **Serum creatine kinase is elevated in patients with Parkinson's disease: a case controlled study.** *Parkinsonism Relat Disord* 2003, **9(Suppl 1):**S43-46.

49. Navarro JA, Jimenez-Jimenez FJ, Molina JA, Benito-Leon J, Cisneros E, Gasalla T, [Ortí-Pareja M](http://www.ncbi.nlm.nih.gov/pubmed?term=Ort%C3%AD-Pareja%20M%5BAuthor%5D&cauthor=true&cauthor_uid=9562329), [Tallón-Barranco A](http://www.ncbi.nlm.nih.gov/pubmed?term=Tall%C3%B3n-Barranco%20A%5BAuthor%5D&cauthor=true&cauthor_uid=9562329), [de Bustos F](http://www.ncbi.nlm.nih.gov/pubmed?term=de%20Bustos%20F%5BAuthor%5D&cauthor=true&cauthor_uid=9562329), [Arenas J](http://www.ncbi.nlm.nih.gov/pubmed?term=Arenas%20J%5BAuthor%5D&cauthor=true&cauthor_uid=9562329): **Cerebrospinal fluid cyclic guanosine 3'5' monophosphate levels in Parkinson's disease.** *J Neurol Sci* 1998, **155(1):**92-94.

50. Nagai Y, Ueno S, Saeki Y, Soga F, Hirano M, Yanagihara T: **Decrease of the D3 dopamine receptor mRNA expression in lymphocytes from patients with Parkinson's disease.** *Neurology* 1996, **46(3):**791-795.

51. Schaf DV, Tort AB, Fricke D, Schestatsky P, Portela LV, Souza DO, Rieder CR: **S100B and NSE serum levels in patients with Parkinson's disease.** *Parkinsonism Relat Disord* 2005, **11(1):**39-43.

52. Hong Z, Shi M, Chung KA, Quinn JF, Peskind ER, Galasko D, [Jankovic J](http://www.ncbi.nlm.nih.gov/pubmed?term=Jankovic%20J%5BAuthor%5D&cauthor=true&cauthor_uid=20157014), [Zabetian CP](http://www.ncbi.nlm.nih.gov/pubmed?term=Zabetian%20CP%5BAuthor%5D&cauthor=true&cauthor_uid=20157014), [Leverenz JB](http://www.ncbi.nlm.nih.gov/pubmed?term=Leverenz%20JB%5BAuthor%5D&cauthor=true&cauthor_uid=20157014), [Baird G](http://www.ncbi.nlm.nih.gov/pubmed?term=Baird%20G%5BAuthor%5D&cauthor=true&cauthor_uid=20157014), [Montine TJ](http://www.ncbi.nlm.nih.gov/pubmed?term=Montine%20TJ%5BAuthor%5D&cauthor=true&cauthor_uid=20157014), [Hancock AM](http://www.ncbi.nlm.nih.gov/pubmed?term=Hancock%20AM%5BAuthor%5D&cauthor=true&cauthor_uid=20157014), [Hwang H](http://www.ncbi.nlm.nih.gov/pubmed?term=Hwang%20H%5BAuthor%5D&cauthor=true&cauthor_uid=20157014), [Pan C](http://www.ncbi.nlm.nih.gov/pubmed?term=Pan%20C%5BAuthor%5D&cauthor=true&cauthor_uid=20157014), [Bradner J](http://www.ncbi.nlm.nih.gov/pubmed?term=Bradner%20J%5BAuthor%5D&cauthor=true&cauthor_uid=20157014), [Kang UJ](http://www.ncbi.nlm.nih.gov/pubmed?term=Kang%20UJ%5BAuthor%5D&cauthor=true&cauthor_uid=20157014), [Jensen PH](http://www.ncbi.nlm.nih.gov/pubmed?term=Jensen%20PH%5BAuthor%5D&cauthor=true&cauthor_uid=20157014), [Zhang J](http://www.ncbi.nlm.nih.gov/pubmed?term=Zhang%20J%5BAuthor%5D&cauthor=true&cauthor_uid=20157014): **DJ-1 and alpha-synuclein in human cerebrospinal fluid as biomarkers of Parkinson's disease.** *Brain* 2010, **133(3):**3-26.

53. Compta Y, Marti MJ, Ibarretxe-Bilbao N, Junque C, Valldeoriola F, Munoz E, [Ezquerra M](http://www.ncbi.nlm.nih.gov/pubmed?term=Ezquerra%20M%5BAuthor%5D&cauthor=true&cauthor_uid=19795497), [Ríos J](http://www.ncbi.nlm.nih.gov/pubmed?term=R%C3%ADos%20J%5BAuthor%5D&cauthor=true&cauthor_uid=19795497), [Tolosa E](http://www.ncbi.nlm.nih.gov/pubmed?term=Tolosa%20E%5BAuthor%5D&cauthor=true&cauthor_uid=19795497): **Cerebrospinal tau, phospho-tau, and beta-amyloid and neuropsychological functions in Parkinson's disease.** *Mov Disord* 2009, **24(15):**2203-2210.

54. Mollenhauer B, Trenkwalder C, von AN, Bibl M, Steinacker P, Brechlin P, [Schindehuette J](http://www.ncbi.nlm.nih.gov/pubmed?term=Schindehuette%20J%5BAuthor%5D&cauthor=true&cauthor_uid=16899997), [Poser S](http://www.ncbi.nlm.nih.gov/pubmed?term=Poser%20S%5BAuthor%5D&cauthor=true&cauthor_uid=16899997), [Wiltfang J](http://www.ncbi.nlm.nih.gov/pubmed?term=Wiltfang%20J%5BAuthor%5D&cauthor=true&cauthor_uid=16899997), [Otto M](http://www.ncbi.nlm.nih.gov/pubmed?term=Otto%20M%5BAuthor%5D&cauthor=true&cauthor_uid=16899997): **Beta-amlyoid 1-42 and tau-protein in cerebrospinal fluid of patients with Parkinson's disease dementia.** *Dement Geriatr Cogn Disord* 2006, **22(3):**200-208.

55. Waragai M, Wei J, Fujita M, Nakai M, Ho GJ, Masliah E, [Akatsu H](http://www.ncbi.nlm.nih.gov/pubmed?term=Akatsu%20H%5BAuthor%5D&cauthor=true&cauthor_uid=16707095), [Yamada T](http://www.ncbi.nlm.nih.gov/pubmed?term=Yamada%20T%5BAuthor%5D&cauthor=true&cauthor_uid=16707095), [Hashimoto M](http://www.ncbi.nlm.nih.gov/pubmed?term=Hashimoto%20M%5BAuthor%5D&cauthor=true&cauthor_uid=16707095): **Increased level of DJ-1 in the cerebrospinal fluids of sporadic Parkinson's disease.** *Biochem Biophys Res Commun* 2006, **345(3):**967-972.

56. Yamada T, Chong JK, Asahina M, Koguchi Y, Hirayama K: **Concentration of neural thread protein in cerebrospinal fluid from progressive supranuclear palsy and Parkinson's disease.** *Jpn J Psychiatry Neurol* 1993, **47(3):**631-635.

Molina JA, Benito-Leon J, Jimenez-Jimenez FJ, Orti-Pareja M, Berbel A, Tallon-Barranco A, [de Bustos F](http://www.ncbi.nlm.nih.gov/pubmed?term=de%20Bustos%20F%5BAuthor%5D&cauthor=true&cauthor_uid=9464639), [Hernánz A](http://www.ncbi.nlm.nih.gov/pubmed?term=Hern%C3%A1nz%20A%5BAuthor%5D&cauthor=true&cauthor_uid=9464639): **Tau protein concentrations in cerebrospinal fluid of non-demented Parkinson's disease patients.** *Neurosci Lett* 1997, **238(3):**139-141.

58. Konings CH, Kuiper MA, Mulder C, Calliauw J, Wolters EC: **CSF acetylcholinesterase in Parkinson disease: decreased enzyme activity and immunoreactivity in demented patients.** *Clin Chim Acta* 1995, **235(1):**101-105.

59. Lunardi G, Galati S, Tropepi D, Moschella V, Brusa L, Pierantozzi M, [Stefani A](http://www.ncbi.nlm.nih.gov/pubmed?term=Stefani%20A%5BAuthor%5D&cauthor=true&cauthor_uid=19010710), [Rossi S](http://www.ncbi.nlm.nih.gov/pubmed?term=Rossi%20S%5BAuthor%5D&cauthor=true&cauthor_uid=19010710), [Fornai F](http://www.ncbi.nlm.nih.gov/pubmed?term=Fornai%20F%5BAuthor%5D&cauthor=true&cauthor_uid=19010710), [Fedele E](http://www.ncbi.nlm.nih.gov/pubmed?term=Fedele%20E%5BAuthor%5D&cauthor=true&cauthor_uid=19010710), [Stanzione P](http://www.ncbi.nlm.nih.gov/pubmed?term=Stanzione%20P%5BAuthor%5D&cauthor=true&cauthor_uid=19010710), [Hainsworth AH](http://www.ncbi.nlm.nih.gov/pubmed?term=Hainsworth%20AH%5BAuthor%5D&cauthor=true&cauthor_uid=19010710), [Pisani A](http://www.ncbi.nlm.nih.gov/pubmed?term=Pisani%20A%5BAuthor%5D&cauthor=true&cauthor_uid=19010710): **Correlation between changes in CSF dopamine turnover and development of dyskinesia in Parkinson's disease.** *Parkinsonism Relat Disord* 2009, **15(5):**383-389.

60. Tohgi H, Abe T, Takahashi S, Ueno M, Nozaki Y: **Cerebrospinal fluid dopamine, norepinephrine, and epinephrine concentrations in Parkinson's disease correlated with clinical symptoms.** *Adv Neurol* 1990, **53:**277-282.

61. Tohgi H, Abe T, Takahashi S, Takahashi J, Nozaki Y, Ueno M, [Kikuchi T](http://www.ncbi.nlm.nih.gov/pubmed?term=Kikuchi%20T%5BAuthor%5D&cauthor=true&cauthor_uid=8094960): **Monoamine metabolism in the cerebrospinal fluid in Parkinson's disease: relationship to clinical symptoms and subsequent therapeutic outcomes.** *J Neural Transm - Parkinsons Disease & Dementia Section* 1993, **5(1):**17-26.

62. Tohgi H, Abe T, Saheki M, Yamazaki K, Murata T: **Concentration of catecholamines and indoleamines in the cerebrospinal fluid of patients with vascular parkinsonism compared to Parkinson's disease patients.** *J Neural Transm* 1997, **104(4-5):**441-449.

63. Strittmatter M, Hamann GF, Strubel D, Cramer H, Schimrigk K: **Somatostatin-like immunoreactivity, its molecular forms and monoaminergic metabolites in aged and demented patients with Parkinson's disease--effect of L-Dopa.** *J Neural Transm* 1996, **103(5):**591-602.

64. Shukla R, Rajani M, Srivastava N, Barthwal MK, Dikshit M: **Nitrite and malondialdehyde content in cerebrospinal fluid of patients with Parkinson's disease.** *Int J Neurosci* 2006, **116(12):**1391-1402.

65. Pall HS, Williams AC, Blake DR, Lunec J, Gutteridge JM, Hall M, Taylor A: **Raised cerebrospinal-fluid copper concentration in Parkinson's disease.** *Lancet* 1987, **2(8553):** 238-241.

66. Jimenez-Jimenez FJ, Molina JA, Hernanz A, Fernandez-Vivancos E, De BF, Barcenilla B, [Gómez-Escalonilla C](http://www.ncbi.nlm.nih.gov/pubmed?term=G%C3%B3mez-Escalonilla%20C%5BAuthor%5D&cauthor=true&cauthor_uid=10471207), [Zurdo M](http://www.ncbi.nlm.nih.gov/pubmed?term=Zurdo%20M%5BAuthor%5D&cauthor=true&cauthor_uid=10471207), [Berbel A](http://www.ncbi.nlm.nih.gov/pubmed?term=Berbel%20A%5BAuthor%5D&cauthor=true&cauthor_uid=10471207), [Villanueva C](http://www.ncbi.nlm.nih.gov/pubmed?term=Villanueva%20C%5BAuthor%5D&cauthor=true&cauthor_uid=10471207): **Cerebrospinal fluid levels of thiamine in patients with Parkinson's disease.** *Neurosci Lett* 1999, **271(1):**33-36.

67. Muller T, Blum-Degen D, Przuntek H, Kuhn W: **Interleukin-6 levels in cerebrospinal fluid inversely correlate to severity of Parkinson's disease.** *Acta Neurol Scand* 1998, **98(2):**142-144.

68. Rainero I, Kaye JA, May C, Durso R, Katz DI, Albert ML, [Wolfe N](http://www.ncbi.nlm.nih.gov/pubmed?term=Wolfe%20N%5BAuthor%5D&cauthor=true&cauthor_uid=2847695), [Pinessi L](http://www.ncbi.nlm.nih.gov/pubmed?term=Pinessi%20L%5BAuthor%5D&cauthor=true&cauthor_uid=2847695), [Friedland RP](http://www.ncbi.nlm.nih.gov/pubmed?term=Friedland%20RP%5BAuthor%5D&cauthor=true&cauthor_uid=2847695), [Rapoport SI](http://www.ncbi.nlm.nih.gov/pubmed?term=Rapoport%20SI%5BAuthor%5D&cauthor=true&cauthor_uid=2847695): **Alpha-melanocyte-stimulating hormonelike immunoreactivity is increased in cerebrospinal fluid of patients with Parkinson's disease.** *Arch Neurol* 1988, **45(11):**1224-1227.

69. Konings CH, Kuiper MA, Bergmans PL, Grijpma AM, van Kamp GJ, Wolters EC: **Increased angiotensin-converting enzyme activity in cerebrospinal fluid of treated patients with Parkinson's disease.** *Clin Chim Acta* 1994, **231(1):**101-106.

70. Jimenez-Jimenez FJ, Molina JA, Vargas C, Gomez P, De BF, Zurdo M, [Gómez-Escalonilla C](http://www.ncbi.nlm.nih.gov/pubmed?term=G%C3%B3mez-Escalonilla%20C%5BAuthor%5D&cauthor=true&cauthor_uid=11215755), [Barcenilla B](http://www.ncbi.nlm.nih.gov/pubmed?term=Barcenilla%20B%5BAuthor%5D&cauthor=true&cauthor_uid=11215755), [Berbel A](http://www.ncbi.nlm.nih.gov/pubmed?term=Berbel%20A%5BAuthor%5D&cauthor=true&cauthor_uid=11215755), [Camacho A](http://www.ncbi.nlm.nih.gov/pubmed?term=Camacho%20A%5BAuthor%5D&cauthor=true&cauthor_uid=11215755), [Arenas J](http://www.ncbi.nlm.nih.gov/pubmed?term=Arenas%20J%5BAuthor%5D&cauthor=true&cauthor_uid=11215755): **Normal cerebrospinal fluid levels of insulin in patients with Parkinson's disease.** *J Neural Transm* 2000, **107(4):**445-449.

71. Vermes I, Steur EN, Jirikowski GF, Haanen C: **Elevated concentration of cerebrospinal fluid tissue transglutaminase in Parkinson's disease indicating apoptosis.** *Mov Disord* 2004, **19(10):**1252-1254.

72. Sato S, Mizuno Y, Hattori N: **Urinary 8-hydroxydeoxyguanosine levels as a biomarker for progression of Parkinson disease.** *Neurology* 2005, **64(6):**1081-1083.

73. Scholz J, Klingemann I, Moser A: **Increased systemic levels of norsalsolinol derivatives are induced by levodopa treatment and do not represent biological markers of Parkinson's disease.** *J Neurol Neurosurg Psychiatry* 2004, **75(4):**634-636.

74. Kim JY, Kim ST, Jeon SH, Lee WY: **Midbrain transcranial sonography in Korean patients with Parkinson's disease.** *Mov Disord* 2007, **22(13):**1922-1926.

75. Weise D, Lorenz R, Schliesser M, Schirbel A, Reiners K, Classen J: **Substantia nigra echogenicity: A structural correlate of functional impairment of the dopaminergic striatal projection in Parkinson's disease.** *Mov Disord* 2009, **24(11):**1669-1675.

76. Berg D, Merz B, Reiners K, Naumann M, Becker G: **Five-year follow-up study of hyperechogenicity of the substantia nigra in Parkinson's disease.** *Mov Disord* 2005, **20(3):**383-385.

77. Berg D, Siefker C, Becker G: **Echogenicity of the substantia nigra in Parkinson's disease and its relation to clinical findings.** *J Neurol* 2001, **248(8):**684-689.

78. Walter U, Dressler D, Wolters A, Wittstock M, Benecke R: **Transcranial brain sonography findings in clinical subgroups of idiopathic Parkinson's disease.** *Mov Disord* 2007, **22(1):**48-54.

79. Haktanir A, Yaman M, Acar M, Gecici O, Demirel R, Albayrak R, [Demirkirkan K](http://www.ncbi.nlm.nih.gov/pubmed?term=Demirkirkan%20K%5BAuthor%5D&cauthor=true&cauthor_uid=16183201): **Evaluation of extracranial blood flow in Parkinson disease.** *Neurosci Lett* 2006, **391(3):**131-135.

80. Hamada K, Hirayama M, Watanabe H, Kobayashi R, Ito H, Ieda T, [Koike Y](http://www.ncbi.nlm.nih.gov/pubmed?term=Koike%20Y%5BAuthor%5D&cauthor=true&cauthor_uid=12640054), [Sobue G](http://www.ncbi.nlm.nih.gov/pubmed?term=Sobue%20G%5BAuthor%5D&cauthor=true&cauthor_uid=12640054): **Onset age and severity of motor impairment are associated with reduction of myocardial 123I-MIBG uptake in Parkinson's disease.** *J Neurol Neurosurg Psychiatry* 2003, **74(4):**423-426.

81. Matsui H, Nishinaka K, Oda M, Komatsu K, Kubori T, Udaka F: **Does cardiac metaiodobenzylguanidine (MIBG) uptake in Parkinson's disease correlate with major autonomic symptoms?** *Parkinsonism Relat Disord* 2006, **12(5):**284-288.

82. Shindo K, Kaneko E, Watanabe H, Sugimoto T, Ohta E, Ohashi K, [Nagasaka T](http://www.ncbi.nlm.nih.gov/pubmed?term=Nagasaka%20T%5BAuthor%5D&cauthor=true&cauthor_uid=16007621), [Shiozawa Z](http://www.ncbi.nlm.nih.gov/pubmed?term=Shiozawa%20Z%5BAuthor%5D&cauthor=true&cauthor_uid=16007621): **Analysis of the relationship between muscle sympathetic nerve activity and cardiac 123I-metaiodobenzylguanidine uptake in patients with Parkinson's disease.** *Mov Disord* 2005, **20(11):**1419-1424.

83. Chun EJ, Lee WY, Yoon WT, Kim BJ, Lee GH: **MIBG scintigraphy for differentiating Parkinson's disease with autonomic dysfunction from parkinsonism-predominant multiple system atrophy.** *Mov Disord* 2009, **24(11):**1650-1655.

84. Orimo S, Ozawa E, Nakade S, Sugimoto T, Mizusawa H: **(123)I-metaiodobenzylguanidine myocardial scintigraphy in Parkinson's disease.** *J Neurol Neurosurg Psychiatry* 1999, **67(2):**189-194.

85. Saiki S, Hirose G, Sakai K, Kataoka S, Hori A, Saiki M, [Kaito M](http://www.ncbi.nlm.nih.gov/pubmed?term=Kaito%20M%5BAuthor%5D&cauthor=true&cauthor_uid=15140615), [Higashi K](http://www.ncbi.nlm.nih.gov/pubmed?term=Higashi%20K%5BAuthor%5D&cauthor=true&cauthor_uid=15140615), [Taki S](http://www.ncbi.nlm.nih.gov/pubmed?term=Taki%20S%5BAuthor%5D&cauthor=true&cauthor_uid=15140615), [Kakeshita K](http://www.ncbi.nlm.nih.gov/pubmed?term=Kakeshita%20K%5BAuthor%5D&cauthor=true&cauthor_uid=15140615), [Fujino S](http://www.ncbi.nlm.nih.gov/pubmed?term=Fujino%20S%5BAuthor%5D&cauthor=true&cauthor_uid=15140615), [Miaki M](http://www.ncbi.nlm.nih.gov/pubmed?term=Miaki%20M%5BAuthor%5D&cauthor=true&cauthor_uid=15140615): **Cardiac 123I-MIBG scintigraphy can assess the disease severity and phenotype of PD.** *J Neurol Sci* 2004, **220(1-2):**105-111.

86. Spiegel J, Mollers M-O, Jost WH, Fuss G, Samnick S, Dillmann U, [Becker G](http://www.ncbi.nlm.nih.gov/pubmed?term=Becker%20G%5BAuthor%5D&cauthor=true&cauthor_uid=15645531), [Kirsch CM](http://www.ncbi.nlm.nih.gov/pubmed?term=Kirsch%20CM%5BAuthor%5D&cauthor=true&cauthor_uid=15645531): **FP-CIT and MIBG scintigraphy in early Parkinson's disease.** *Mov Disord* 2005, **20(5):**552-561.

87. Nagayama H, Hamamoto M, Ueda M, Nagashima J, Katayama Y: **Reliability of MIBG myocardial scintigraphy in the diagnosis of Parkinson's disease.** *J Neurol Neurosurg Psychiatry* 2005, **76(2):**249-251.

88. Satoh A, Serita T, Seto M, Tomita I, Satoh H, Iwanaga K, [Takashima H](http://www.ncbi.nlm.nih.gov/pubmed?term=Takashima%20H%5BAuthor%5D&cauthor=true&cauthor_uid=10086697), [Tsujihata M](http://www.ncbi.nlm.nih.gov/pubmed?term=Tsujihata%20M%5BAuthor%5D&cauthor=true&cauthor_uid=10086697): **Loss of 123I-MIBG uptake by the heart in Parkinson's disease: assessment of cardiac sympathetic denervation and diagnostic value.** *J Nucl Med* 1999, **40(3):**371-375.

89. Duguid JR, De La Paz R, DeGroot J: **Magnetic resonance imaging of the midbrain in Parkinson's disease.** *Ann Neurol* 1986, **20(6):**744-747.

90. Atasoy HT, Nuyan O, Tunc T, Yorubulut M, Unal AE, Inan LE: **T2-weighted MRI in Parkinson's disease; substantia nigra pars compacta hypointensity correlates with the clinical scores.** *Neurol India* 2004, **52(3):**332-337.

91. Chan LL, Rumpel H, Yap K, Lee E, Loo HV, Ho GL, [Fook-Chong S](http://www.ncbi.nlm.nih.gov/pubmed?term=Fook-Chong%20S%5BAuthor%5D&cauthor=true&cauthor_uid=17615165), [Yuen Y](http://www.ncbi.nlm.nih.gov/pubmed?term=Yuen%20Y%5BAuthor%5D&cauthor=true&cauthor_uid=17615165), [Tan EK](http://www.ncbi.nlm.nih.gov/pubmed?term=Tan%20EK%5BAuthor%5D&cauthor=true&cauthor_uid=17615165): **Case control study of diffusion tensor imaging in Parkinson's disease.** *J Neurol Neurosurg Psychiatry* 2007, **78(12):**1383-1386.

92. Martin WR, Wieler M, Gee M: **Midbrain iron content in early Parkinson disease: a potential biomarker of disease status.** *Neurology* 2008, **70(16 Pt 2):**1411-1417.

93. Gorell JM, Ordidge RJ, Brown GG, Deniau JC, Buderer NM, Helpern JA: **Increased iron-related MRI contrast in the substantia nigra in Parkinson's disease.** *Neurology* 1995, **45(6):**1138-1143.

94. Wallis LI, Paley MNJ, Graham JM, Grunewald RA, Wignall EL, Joy HM, Griffiths PD: **MRI assessment of basal ganglia iron deposition in Parkinson's disease.** *J Magn Reson Imaging* 2008, **28(5):**1061-1067.

95. Graham JM, Paley MN, Grunewald RA, Hoggard N, Griffiths PD: **Brain iron deposition in Parkinson's disease imaged using the PRIME magnetic resonance sequence.** *Brain* 2000, **123(12):** 2423-2431.

96. Zhang J, Zhang Y, Wang J, Cai P, Luo C, Qian Z, Dai Y, Feng H: **Characterizing iron deposition in Parkinson's disease using susceptibility-weighted imaging: an in vivo MR study.** *Brain Res* 2010, **1330:**124-130.

97. Antonini A, Leenders KL, Meier D, Oertel WH, Boesiger P, Anliker M: **T2 relaxation time in patients with Parkinson's disease.** *Neurology* 1993, **43(4):**697-700.

98. Hu MT, Scherfler C, Khan NL, Hajnal JV, Lees AJ, Quinn N, Wood NW, Brooks DJ: **Nigral degeneration and striatal dopaminergic dysfunction in idiopathic and Parkin-linked Parkinson's disease.** *Mov Disord* 2006, **21(3):**299-305.

99. Ye FQ, Allen PS, Martin WR: **Basal ganglia iron content in Parkinson's disease measured with magnetic resonance.** *Mov Disord* 1996, **11(3):**243-249.

100. Junque C, Ramirez-Ruiz B, Tolosa E, Summerfield C, Marti MJ, Pastor P, [Gómez-Ansón B](http://www.ncbi.nlm.nih.gov/pubmed?term=G%C3%B3mez-Ans%C3%B3n%20B%5BAuthor%5D&cauthor=true&cauthor_uid=15645532), [Mercader JM](http://www.ncbi.nlm.nih.gov/pubmed?term=Mercader%20JM%5BAuthor%5D&cauthor=true&cauthor_uid=15645532): **Amygdalar and hippocampal MRI volumetric reductions in Parkinson's disease with dementia.** *Mov Disord* 2005, **20(5):**540-544.

101. Tam CW, Burton EJ, McKeith IG, Burn DJ, O'Brien JT: **Temporal lobe atrophy on MRI in Parkinson disease with dementia: a comparison with Alzheimer disease and dementia with Lewy bodies.** *Neurology* 2005, **64(5):**861-865.

102. Linder J, Birgander R, Olsson I, Riklund K, Larsson AK, Edstrom M, [Stenlund H](http://www.ncbi.nlm.nih.gov/pubmed?term=Stenlund%20H%5BAuthor%5D&cauthor=true&cauthor_uid=19484399), [Forsgren L](http://www.ncbi.nlm.nih.gov/pubmed?term=Forsgren%20L%5BAuthor%5D&cauthor=true&cauthor_uid=19484399): **Degenerative changes were common in brain magnetic resonance imaging in patients with newly diagnosed Parkinson's disease in a population-based cohort.** *J Neurol* 2009, **256(10):**1671-1680.

103. Summerfield C, Gomez-Anson B, Tolosa E, Mercader JM, Marti MJ, Pastor P, [Junqué C](http://www.ncbi.nlm.nih.gov/pubmed?term=Junqu%C3%A9%20C%5BAuthor%5D&cauthor=true&cauthor_uid=12223027): **Dementia in Parkinson disease: a proton magnetic resonance spectroscopy study.** *Arch Neurol* 2002, **59(9):**1415-1420.

104. Hu MT, Taylor-Robinson SD, Chaudhuri KR, Bell JD, Morris RG, Clough C, [Brooks DJ](http://www.ncbi.nlm.nih.gov/pubmed?term=Brooks%20DJ%5BAuthor%5D&cauthor=true&cauthor_uid=10369817), [Turjanski N](http://www.ncbi.nlm.nih.gov/pubmed?term=Turjanski%20N%5BAuthor%5D&cauthor=true&cauthor_uid=10369817): **Evidence for cortical dysfunction in clinically non-demented patients with Parkinson's disease: a proton MR spectroscopy study.** *J Neurol Neurosurg Psychiatry* 1999, **67(1):**20-26.

105. Benamer HTS, Patterson J, Wyper DJ, Hadley DM, Macphee GJA, Grosset DG: **Correlation of Parkinson's disease severity and duration with 123I-FP-CIT SPECT striatal uptake.** *Mov Disord* 2000, **15(4):**692-698.

106. Eshuis SA, Maguire RP, Leenders KL, Jonkman S, Jager PL: **Comparison of FP-CIT SPECT with F-DOPA PET in patients with de novo and advanced Parkinson's disease.** *Eur J Nucl Med Mol Imaging* 2006, **33(2):**200-209.

107. Tissingh G, Booij J, Bergmans P, Winogrodzka A, Janssen AG, van Royen EA, [Stoof JC](http://www.ncbi.nlm.nih.gov/pubmed?term=Stoof%20JC%5BAuthor%5D&cauthor=true&cauthor_uid=9669384), [Wolters EC](http://www.ncbi.nlm.nih.gov/pubmed?term=Wolters%20EC%5BAuthor%5D&cauthor=true&cauthor_uid=9669384): **Iodine-123-N-omega-fluoropropyl-2beta-carbomethoxy-3beta-(4-iod ophenyl)tropane SPECT in healthy controls and early-stage, drug-naive Parkinson's disease.** *J Nucl Med* 1998, **39(7):**1143-1148.

108. Booij J, Tissingh G, Boer GJ, Speelman JD, Stoof JC, Janssen AG, Wolters EC, van Royen EA: **[123I]FP-CIT SPECT shows a pronounced decline of striatal dopamine transporter labelling in early and advanced Parkinson's disease.** *J Neurol Neurosurg Psychiatry* 1997, **62(2):**133-140.

109. Nobili F, Campus C, Arnaldi D, De CF, Cabassi G, Brugnolo A, [Dessi B](http://www.ncbi.nlm.nih.gov/pubmed?term=Dessi%20B%5BAuthor%5D&cauthor=true&cauthor_uid=20058228), [Morbelli S](http://www.ncbi.nlm.nih.gov/pubmed?term=Morbelli%20S%5BAuthor%5D&cauthor=true&cauthor_uid=20058228), [Sambuceti G](http://www.ncbi.nlm.nih.gov/pubmed?term=Sambuceti%20G%5BAuthor%5D&cauthor=true&cauthor_uid=20058228), [Abbruzzese G](http://www.ncbi.nlm.nih.gov/pubmed?term=Abbruzzese%20G%5BAuthor%5D&cauthor=true&cauthor_uid=20058228), [Rodriguez G](http://www.ncbi.nlm.nih.gov/pubmed?term=Rodriguez%20G%5BAuthor%5D&cauthor=true&cauthor_uid=20058228): **Cognitive-nigrostriatal relationships in de novo, drug-naive Parkinson's disease patients: a [I-123]FP-CIT SPECT study.** *Mov Disord* 2010, **25(1):**35-43.

110. Winogrodzka A, Bergmans P, Booij J, van Royen EA, Stoof JC, Wolters EC: **[(123)I]beta-CIT SPECT is a useful method for monitoring dopaminergic degeneration in early stage Parkinson's disease.** *J Neurol Neurosurg Psychiatry* 2003, **74(3):**294-298.

111. Tissingh G, Bergmans P, Booij J, Winogrodzka A, van Royen EA, Stoof JC, Wolters EC: **Drug-naive patients with Parkinson's disease in Hoehn and Yahr stages I and II show a bilateral decrease in striatal dopamine transporters as revealed by [123I]beta-CIT SPECT.** *J Neurol* 1998, **245(1):**14-20.

112. Muller U, Wachter T, Barthel H, Reuter M, von Cramon DY: **Striatal [123I]beta-CIT SPECT and prefrontal cognitive functions in Parkinson's disease.** *J Neural Transm* 2000, **107(3):**303-319.

113. Shinotoh H, Uchida Y, Ito H, Harrori T: **Relationship between striatal [123I]beta-CIT binding and four major clinical signs in Parkinson's disease.** *Ann Nucl Med* 2000, **14(3):**199-203.

114. Seibyl JP, Marchek KL, Quinlan D, Sheff K, Zoghbi S, Zea-Ponce Y, [Baldwin RM](http://www.ncbi.nlm.nih.gov/pubmed?term=Baldwin%20RM%5BAuthor%5D&cauthor=true&cauthor_uid=7574455), [Fussell B](http://www.ncbi.nlm.nih.gov/pubmed?term=Fussell%20B%5BAuthor%5D&cauthor=true&cauthor_uid=7574455), [Smith EO](http://www.ncbi.nlm.nih.gov/pubmed?term=Smith%20EO%5BAuthor%5D&cauthor=true&cauthor_uid=7574455), [Charney DS](http://www.ncbi.nlm.nih.gov/pubmed?term=Charney%20DS%5BAuthor%5D&cauthor=true&cauthor_uid=7574455), [Hoffer](http://www.ncbi.nlm.nih.gov/pubmed?term=van%20Dyck%20C%5BAuthor%5D&cauthor=true&cauthor_uid=7574455) PB, Innis RB: **Decreased single-photon emission computed tomographic [123I]beta-CIT striatal uptake correlates with symptom severity in Parkinson's disease.** *Ann Neurol* 1995, **38(4):**589-598.

115. Ichise M, Kim YJ, Ballinger JR, Vines D, Erami SS, Tanaka F, Lang AE. **SPECT imaging of pre- and postsynaptic dopaminergic alterations in L-dopa-untreated PD.** *Neurology* 1999, **52(6):**1206-1214.

116. Tatsch K, Schwarz J, Mozley PD, Linke R, Pogarell O, Oertel WH, [Fieber RS](http://www.ncbi.nlm.nih.gov/pubmed?term=Fieber%20RS%5BAuthor%5D&cauthor=true&cauthor_uid=9096093), [Hahn K](http://www.ncbi.nlm.nih.gov/pubmed?term=Hahn%20K%5BAuthor%5D&cauthor=true&cauthor_uid=9096093), [Kung HF](http://www.ncbi.nlm.nih.gov/pubmed?term=Kung%20HF%5BAuthor%5D&cauthor=true&cauthor_uid=9096093): **Relationship between clinical features of Parkinson's disease and presynaptic dopamine transporter binding assessed with [123I]IPT and single-photon emission tomography.** *European J Nucl Med* 1997, **24(4):**415-421.

117. Prunier C, Payoux P, Guilloteau D, Chalon S, Giraudeau B, Majorel C, [Tafani M](http://www.ncbi.nlm.nih.gov/pubmed?term=Tafani%20M%5BAuthor%5D&cauthor=true&cauthor_uid=12732666), [Bezard E](http://www.ncbi.nlm.nih.gov/pubmed?term=Bezard%20E%5BAuthor%5D&cauthor=true&cauthor_uid=12732666), [Esquerré JP](http://www.ncbi.nlm.nih.gov/pubmed?term=Esquerr%C3%A9%20JP%5BAuthor%5D&cauthor=true&cauthor_uid=12732666), [Baulieu JL](http://www.ncbi.nlm.nih.gov/pubmed?term=Baulieu%20JL%5BAuthor%5D&cauthor=true&cauthor_uid=12732666): **Quantification of dopamine transporter by 123I-PE2I SPECT and the noninvasive Logan graphical method in Parkinson's disease.** *J Nucl Med* 2003, **44(5):**663-670.

118. Geng Y, Shi GH, Jiang Y, Xu LX, Hu XY, Shao YQ: **Investigating the role of 99mTc-TRODAT-1 SPECT imaging in idiopathic Parkinson's disease.** *J Zhejiang Univ Sci B* 2005, **6(1):** 22-27.

119. Huang WS, Lee MS, Lin JC, Chen CY, Yang YW, Lin SZ, Wey SP: **Usefulness of brain 99mTc-TRODAT-1 SPET for the evaluation of Parkinson's disease.** *Eur J Nucl Med Mol Imaging* 2004, **31(2):**155-161.

120. Siderowf A, Newberg A, Chou KL, Lloyd M, Colcher A, Hurtig HI, [Stern MB](http://www.ncbi.nlm.nih.gov/pubmed?term=Stern%20MB%5BAuthor%5D&cauthor=true&cauthor_uid=15911797), [Doty RL](http://www.ncbi.nlm.nih.gov/pubmed?term=Doty%20RL%5BAuthor%5D&cauthor=true&cauthor_uid=15911797), [Mozley PD](http://www.ncbi.nlm.nih.gov/pubmed?term=Mozley%20PD%5BAuthor%5D&cauthor=true&cauthor_uid=15911797), [Wintering N](http://www.ncbi.nlm.nih.gov/pubmed?term=Wintering%20N%5BAuthor%5D&cauthor=true&cauthor_uid=15911797), [Duda JE](http://www.ncbi.nlm.nih.gov/pubmed?term=Duda%20JE%5BAuthor%5D&cauthor=true&cauthor_uid=15911797), [Weintraub D](http://www.ncbi.nlm.nih.gov/pubmed?term=Weintraub%20D%5BAuthor%5D&cauthor=true&cauthor_uid=15911797), [Moberg PJ](http://www.ncbi.nlm.nih.gov/pubmed?term=Moberg%20PJ%5BAuthor%5D&cauthor=true&cauthor_uid=15911797): **[99mTc]TRODAT-1 SPECT imaging correlates with odor identification in early Parkinson disease.** *Neurology* 2005, **64(10):**1716-1720.

121. Huang WS, Lin SZ, Lin JC, Wey SP, Ting G, Liu RS: **Evaluation of early-stage Parkinson's disease with 99mTc-TRODAT-1 imaging.** *J Nucl Med* 2001, **42(9):**1303-1308.

122. Weng YH, Yen TC, Chen MC, Kao PF, Tzen KY, Chen RS, [Wey SP](http://www.ncbi.nlm.nih.gov/pubmed?term=Wey%20SP%5BAuthor%5D&cauthor=true&cauthor_uid=15001678), [Ting G](http://www.ncbi.nlm.nih.gov/pubmed?term=Ting%20G%5BAuthor%5D&cauthor=true&cauthor_uid=15001678), [Lu CS](http://www.ncbi.nlm.nih.gov/pubmed?term=Lu%20CS%5BAuthor%5D&cauthor=true&cauthor_uid=15001678): **Sensitivity and specificity of 99mTc-TRODAT-1 SPECT imaging in differentiating patients with idiopathic Parkinson's disease from healthy subjects.** *J Nucl Med* 2004, **45(3):**393-401.

123. Ottaviani S, Tinazzi M, Pasquin I, Nothdurfter W, Tomelleri G, Fincati E, [Smania N](http://www.ncbi.nlm.nih.gov/pubmed?term=Smania%20N%5BAuthor%5D&cauthor=true&cauthor_uid=17205224), [Giorgetti P](http://www.ncbi.nlm.nih.gov/pubmed?term=Giorgetti%20P%5BAuthor%5D&cauthor=true&cauthor_uid=17205224), [Antonini A](http://www.ncbi.nlm.nih.gov/pubmed?term=Antonini%20A%5BAuthor%5D&cauthor=true&cauthor_uid=17205224): **Comparative analysis of visual and semi-quantitative assessment of striatal [123I]FP-CIT-SPET binding in Parkinson's disease.** *Neurol Sci* 2006, **27(6):**397-401.

124. Haapaniemi TH, Ahonen A, Torniainen P, Sotaniemi KA, Myllyla VV: **[123]beta-CIT SPECT demonstrates decreased brain dopamine and serotonin transporter levels in untreated parkinsonian patients.** *Mov Disord* 2001, **16(1):**124-130.

125. Brucke T, Asenbaum S, Pirker W, Djamshidian S, Wenger S, Wober C, [Müller C](http://www.ncbi.nlm.nih.gov/pubmed?term=M%C3%BCller%20C%5BAuthor%5D&cauthor=true&cauthor_uid=9120429), [Podreka I](http://www.ncbi.nlm.nih.gov/pubmed?term=Podreka%20I%5BAuthor%5D&cauthor=true&cauthor_uid=9120429): **Measurement of the dopaminergic degeneration in Parkinson's disease with [123I] beta-CIT and SPECT. Correlation with clinical findings and comparison with multiple system atrophy and progressive supranuclear palsy.** *J Neural Transm Suppl* 1997, **50:**9-24.

126. Kim SE, Lee WY, Choe YS, Kim JH: **SPECT measurement of iodine-123-beta-CIT binding to dopamine and serotonin transporters in Parkinson's disease: correlation with symptom severity.** *Neurol Res* 1999, **21(3):**255-261.

127. Hwang WJ, Yao WJ, Wey SP, Ting G: **Reproducibility of 99mTc-TRODAT-1 SPECT measurement of dopamine transporters in Parkinson's disease.** *J Nucl Med* 2004, **45(2):**207-213.

128. Bao SY, Wu JC, Luo WF, Fang P, Liu ZL, Tang J: **Imaging of dopamine transporters with technetium-99m TRODAT-1 and single photon emission computed tomography.** *J Neuroimaging* 2000, **10(4):** 200-203.

129. Derejko M, Slawek J, Wieczorek D, Brockhuis B, Dubaniewicz M, Lass P: **Regional cerebral blood flow in Parkinson's disease as an indicator of cognitive impairment.** *Nucl Med Commun* 2006, **27(12):**945-951.

130. Matsui H, Udaka F, Miyoshi T, Hara N, Tamaura A, Oda M, [Kubori T](http://www.ncbi.nlm.nih.gov/pubmed?term=Kubori%20T%5BAuthor%5D&cauthor=true&cauthor_uid=16007622), [Nishinaka K](http://www.ncbi.nlm.nih.gov/pubmed?term=Nishinaka%20K%5BAuthor%5D&cauthor=true&cauthor_uid=16007622), [Kameyama M](http://www.ncbi.nlm.nih.gov/pubmed?term=Kameyama%20M%5BAuthor%5D&cauthor=true&cauthor_uid=16007622): **Three-dimensional stereotactic surface projection study of freezing of gait and brain perfusion image in Parkinson's disease.** *Mov Disord* 2005, **20(10):**1272-1277.

131. Paschali A, Messinis L, Kargiotis O, Lakiotis V, Kefalopoulou Z, Constantoyannis C, [Papathanasopoulos P](http://www.ncbi.nlm.nih.gov/pubmed?term=Papathanasopoulos%20P%5BAuthor%5D&cauthor=true&cauthor_uid=20145921), [Vassilakos P](http://www.ncbi.nlm.nih.gov/pubmed?term=Vassilakos%20P%5BAuthor%5D&cauthor=true&cauthor_uid=20145921): **SPECT neuroimaging and neuropsychological functions in different stages of Parkinson's disease.** *Eur J Nucl Med Mol Imaging* 2010, **37(6):**1128-1140.

132. Feigin A, Antonini A, Fukuda M, De NR, Benti R, Pezzoli G, [Mentis MJ](http://www.ncbi.nlm.nih.gov/pubmed?term=Mentis%20MJ%5BAuthor%5D&cauthor=true&cauthor_uid=12465066), [Moeller JR](http://www.ncbi.nlm.nih.gov/pubmed?term=Moeller%20JR%5BAuthor%5D&cauthor=true&cauthor_uid=12465066), [Eidelberg D](http://www.ncbi.nlm.nih.gov/pubmed?term=Eidelberg%20D%5BAuthor%5D&cauthor=true&cauthor_uid=12465066): **Tc-99m ethylene cysteinate dimer SPECT in the differential diagnosis of parkinsonism.** *Mov Disord* 2002, **17(6):**1265-1270.

133. Hsu JL, Jung TP, Hsu CY, Hsu WC, Chen YK, Duann JR, [Wang HC](http://www.ncbi.nlm.nih.gov/pubmed?term=Wang%20HC%5BAuthor%5D&cauthor=true&cauthor_uid=17437108), [Makeig S](http://www.ncbi.nlm.nih.gov/pubmed?term=Makeig%20S%5BAuthor%5D&cauthor=true&cauthor_uid=17437108): **Regional CBF changes in Parkinson's disease: a correlation with motor dysfunction.** *Eur J Nucl Med Mol Imaging* 2007, **34(9):**1458-1466.

134. Kikuchi A, Takeda A, Kimpara T, Nakagawa M, Kawashima R, Sugiura M [Kinomura S](http://www.ncbi.nlm.nih.gov/pubmed?term=Kinomura%20S%5BAuthor%5D&cauthor=true&cauthor_uid=11718747), [Fukuda H](http://www.ncbi.nlm.nih.gov/pubmed?term=Fukuda%20H%5BAuthor%5D&cauthor=true&cauthor_uid=11718747), [Chida K](http://www.ncbi.nlm.nih.gov/pubmed?term=Chida%20K%5BAuthor%5D&cauthor=true&cauthor_uid=11718747), [Okita N](http://www.ncbi.nlm.nih.gov/pubmed?term=Okita%20N%5BAuthor%5D&cauthor=true&cauthor_uid=11718747), [Takase S](http://www.ncbi.nlm.nih.gov/pubmed?term=Takase%20S%5BAuthor%5D&cauthor=true&cauthor_uid=11718747), [Itoyama Y](http://www.ncbi.nlm.nih.gov/pubmed?term=Itoyama%20Y%5BAuthor%5D&cauthor=true&cauthor_uid=11718747): **Hypoperfusion in the supplementary motor area, dorsolateral prefrontal cortex and insular cortex in Parkinson's disease.** *J Neurol Sci* 2001, **193(1):**29-36.

135. Ribeiro MJ, Vidailhet M, Loc'h C, Dupel C, Nguyen JP, Ponchant M, [Dollé F](http://www.ncbi.nlm.nih.gov/pubmed?term=Doll%C3%A9%20F%5BAuthor%5D&cauthor=true&cauthor_uid=11939892), [Peschanski M](http://www.ncbi.nlm.nih.gov/pubmed?term=Peschanski%20M%5BAuthor%5D&cauthor=true&cauthor_uid=11939892), [Hantraye P](http://www.ncbi.nlm.nih.gov/pubmed?term=Hantraye%20P%5BAuthor%5D&cauthor=true&cauthor_uid=11939892), [Cesaro P](http://www.ncbi.nlm.nih.gov/pubmed?term=Cesaro%20P%5BAuthor%5D&cauthor=true&cauthor_uid=11939892), [Samson Y](http://www.ncbi.nlm.nih.gov/pubmed?term=Samson%20Y%5BAuthor%5D&cauthor=true&cauthor_uid=11939892), [Remy P](http://www.ncbi.nlm.nih.gov/pubmed?term=Remy%20P%5BAuthor%5D&cauthor=true&cauthor_uid=11939892): **Dopaminergic function and dopamine transporter binding assessed with positron emission tomography in Parkinson disease.** *Arch Neurol* 2002, **59(4):**580-586.

136. Antonini A, Vontobel P, Psylla M, Gunther I, Maguire PR, Missimer J, [Leenders KL](http://www.ncbi.nlm.nih.gov/pubmed?term=Leenders%20KL%5BAuthor%5D&cauthor=true&cauthor_uid=7492293): **Complementary positron emission tomographic studies of the striatal dopaminergic system in Parkinson's disease.** *Arch Neurol* 1995, **52(12):**1183-1190.

137. Morrish PK, Sawle GV, Brooks DJ: **Clinical and [18F] dopa PET findings in early Parkinson's disease.** *J Neurol Neurosurg Psychiatry* 1995, **59(6):**597-600.

138. Nagasawa H, Saito H, Kogure K, Hatazawa J, Itoh M, Fujiwara T, [Watanuki S](http://www.ncbi.nlm.nih.gov/pubmed?term=Watanuki%20S%5BAuthor%5D&cauthor=true&cauthor_uid=8482975), [Seo S](http://www.ncbi.nlm.nih.gov/pubmed?term=Seo%20S%5BAuthor%5D&cauthor=true&cauthor_uid=8482975), [Iwata R](http://www.ncbi.nlm.nih.gov/pubmed?term=Iwata%20R%5BAuthor%5D&cauthor=true&cauthor_uid=8482975), [Ido T](http://www.ncbi.nlm.nih.gov/pubmed?term=Ido%20T%5BAuthor%5D&cauthor=true&cauthor_uid=8482975): **6-[18F]fluorodopa metabolism in patients with hemiparkinsonism studied by positron emission tomography.** *J Neurol Sci* 1993, **115(2):**136-143.

139. Vingerhoets FJ, Schulzer M, Calne DB, Snow BJ: **Which clinical sign of Parkinson's disease best reflects the nigrostriatal lesion?** *Ann Neurol* 1997, **41(1):**58-64.

140. Broussolle E, Dentresangle C, Landais P, Garcia-Larrea L, Pollak P, Croisile B, [Hibert O](http://www.ncbi.nlm.nih.gov/pubmed?term=Hibert%20O%5BAuthor%5D&cauthor=true&cauthor_uid=10475108), [Bonnefoi F](http://www.ncbi.nlm.nih.gov/pubmed?term=Bonnefoi%20F%5BAuthor%5D&cauthor=true&cauthor_uid=10475108), [Galy G](http://www.ncbi.nlm.nih.gov/pubmed?term=Galy%20G%5BAuthor%5D&cauthor=true&cauthor_uid=10475108), [Froment JC](http://www.ncbi.nlm.nih.gov/pubmed?term=Froment%20JC%5BAuthor%5D&cauthor=true&cauthor_uid=10475108), [Comar D](http://www.ncbi.nlm.nih.gov/pubmed?term=Comar%20D%5BAuthor%5D&cauthor=true&cauthor_uid=10475108): **The relation of putamen and caudate nucleus 18F-Dopa uptake to motor and cognitive performances in Parkinson's disease.** *J Neurol Sci* 1999, **166(2):**141-151.

141. Brooks DJ, Ibanez V, Sawle GV, Quinn N, Lees AJ, Mathias CJ, [Bannister R](http://www.ncbi.nlm.nih.gov/pubmed?term=Bannister%20R%5BAuthor%5D&cauthor=true&cauthor_uid=2132742), [Marsden CD](http://www.ncbi.nlm.nih.gov/pubmed?term=Marsden%20CD%5BAuthor%5D&cauthor=true&cauthor_uid=2132742), [Frackowiak RS](http://www.ncbi.nlm.nih.gov/pubmed?term=Frackowiak%20RS%5BAuthor%5D&cauthor=true&cauthor_uid=2132742): **Differing patterns of striatal 18F-dopa uptake in Parkinson's disease, multiple system atrophy, and progressive supranuclear palsy.** *Ann Neurol* 1990, **28(4):**547-555.

142. Otsuka M, Ichiya Y, Kuwabara Y, Hosokawa S, Sasaki M, Yoshida T, [Fukumura T](http://www.ncbi.nlm.nih.gov/pubmed?term=Fukumura%20T%5BAuthor%5D&cauthor=true&cauthor_uid=8815166), [Masuda K](http://www.ncbi.nlm.nih.gov/pubmed?term=Masuda%20K%5BAuthor%5D&cauthor=true&cauthor_uid=8815166), [Kato M](http://www.ncbi.nlm.nih.gov/pubmed?term=Kato%20M%5BAuthor%5D&cauthor=true&cauthor_uid=8815166): **Differences in the reduced 18F-Dopa uptakes of the caudate and the putamen in Parkinson's disease: correlations with the three main symptoms.** *J Neurol Sci* 1996, **136(1-2):**169-173.

143. Nagasawa H, Tanji H, Itoyama Y, Saito H, Kimura I, Fujiwara T, [Iwata R](http://www.ncbi.nlm.nih.gov/pubmed?term=Iwata%20R%5BAuthor%5D&cauthor=true&cauthor_uid=8994106), [Itoh M](http://www.ncbi.nlm.nih.gov/pubmed?term=Itoh%20M%5BAuthor%5D&cauthor=true&cauthor_uid=8994106), [Ido T](http://www.ncbi.nlm.nih.gov/pubmed?term=Ido%20T%5BAuthor%5D&cauthor=true&cauthor_uid=8994106): **Brain 6-[18F]fluorodopa metabolism in early and late onset of Parkinson's disease studied by positron emission tomography.** *J Neurol Sci* 1996, **144(1-2):**70-76.

144. Holthoff-Detto VA, Kessler J, Herholz K, Bonner H, Pietrzyk U, Wurker M, [Ghaemi M](http://www.ncbi.nlm.nih.gov/pubmed?term=Ghaemi%20M%5BAuthor%5D&cauthor=true&cauthor_uid=9041855), [Wienhard K](http://www.ncbi.nlm.nih.gov/pubmed?term=Wienhard%20K%5BAuthor%5D&cauthor=true&cauthor_uid=9041855), [Wagner R](http://www.ncbi.nlm.nih.gov/pubmed?term=Wagner%20R%5BAuthor%5D&cauthor=true&cauthor_uid=9041855), [Heiss WD](http://www.ncbi.nlm.nih.gov/pubmed?term=Heiss%20WD%5BAuthor%5D&cauthor=true&cauthor_uid=9041855): **Functional effects of striatal dysfunction in Parkinson disease.** *Arch Neurol* 1997, **54(2):**145-150.

145. Nandhagopal R, Kuramoto L, Schulzer M, Mak E, Cragg J, Lee CS, [McKenzie J](http://www.ncbi.nlm.nih.gov/pubmed?term=McKenzie%20J%5BAuthor%5D&cauthor=true&cauthor_uid=19690093), [McCormick S](http://www.ncbi.nlm.nih.gov/pubmed?term=McCormick%20S%5BAuthor%5D&cauthor=true&cauthor_uid=19690093), [Samii A](http://www.ncbi.nlm.nih.gov/pubmed?term=Samii%20A%5BAuthor%5D&cauthor=true&cauthor_uid=19690093), [Troiano A](http://www.ncbi.nlm.nih.gov/pubmed?term=Troiano%20A%5BAuthor%5D&cauthor=true&cauthor_uid=19690093), [Ruth TJ](http://www.ncbi.nlm.nih.gov/pubmed?term=Ruth%20TJ%5BAuthor%5D&cauthor=true&cauthor_uid=19690093), [Sossi V](http://www.ncbi.nlm.nih.gov/pubmed?term=Sossi%20V%5BAuthor%5D&cauthor=true&cauthor_uid=19690093), [de la Fuente-Fernandez R](http://www.ncbi.nlm.nih.gov/pubmed?term=de%20la%20Fuente-Fernandez%20R%5BAuthor%5D&cauthor=true&cauthor_uid=19690093), [Calne DB](http://www.ncbi.nlm.nih.gov/pubmed?term=Calne%20DB%5BAuthor%5D&cauthor=true&cauthor_uid=19690093), [Stoessl AJ](http://www.ncbi.nlm.nih.gov/pubmed?term=Stoessl%20AJ%5BAuthor%5D&cauthor=true&cauthor_uid=19690093): **Longitudinal progression of sporadic Parkinson's disease: a multi-tracer positron emission tomography study.** *Brain* 2009, **132(11):**11-19.

146. Wang J, Zuo CT, Jiang YP, Guan YH, Chen ZP, Xiang JD, [Yang LQ](http://www.ncbi.nlm.nih.gov/pubmed?term=Yang%20LQ%5BAuthor%5D&cauthor=true&cauthor_uid=17334953), [Ding ZT](http://www.ncbi.nlm.nih.gov/pubmed?term=Ding%20ZT%5BAuthor%5D&cauthor=true&cauthor_uid=17334953), [Wu JJ](http://www.ncbi.nlm.nih.gov/pubmed?term=Wu%20JJ%5BAuthor%5D&cauthor=true&cauthor_uid=17334953), [Su HL](http://www.ncbi.nlm.nih.gov/pubmed?term=Su%20HL%5BAuthor%5D&cauthor=true&cauthor_uid=17334953): **18F-FP-CIT PET imaging and SPM analysis of dopamine transporters in Parkinson's disease in various Hoehn & Yahr stages.** *J Neurol* 2007, **254(2):**185-190.

147. Bohnen NI, Albin RL, Koeppe RA, Wernette KA, Kilbourn MR, Minoshima S, Frey KA: **Positron emission tomography of monoaminergic vesicular binding in aging and Parkinson disease.** *Journal of Cerebral Blood Flow & Metabolism* 2006, **26(9):**1198-1212.

148. Martin WR, Wieler M, Stoessl AJ, Schulzer M: **Dihydrotetrabenazine positron emission tomography imaging in early, untreated Parkinson's disease.** *Ann Neurol* 2008, **63(3):**388-394.

149. Breit S, Reimold M, Reischl G, Klockgether T, Wullner U: **[(11)C]d-threo-methylphenidate PET in patients with Parkinson's disease and essential tremor.** *J Neural Transm* 2006, **113(2):**187-193.

150. Kas A, Bottlaender M, Gallezot JD, Vidailhet M, Villafane G, Gregoire MC [Coulon C](http://www.ncbi.nlm.nih.gov/pubmed?term=Coulon%20C%5BAuthor%5D&cauthor=true&cauthor_uid=19491921), [Valette H](http://www.ncbi.nlm.nih.gov/pubmed?term=Valette%20H%5BAuthor%5D&cauthor=true&cauthor_uid=19491921), [Dollé F](http://www.ncbi.nlm.nih.gov/pubmed?term=Doll%C3%A9%20F%5BAuthor%5D&cauthor=true&cauthor_uid=19491921), [Ribeiro MJ](http://www.ncbi.nlm.nih.gov/pubmed?term=Ribeiro%20MJ%5BAuthor%5D&cauthor=true&cauthor_uid=19491921), [Hantraye P](http://www.ncbi.nlm.nih.gov/pubmed?term=Hantraye%20P%5BAuthor%5D&cauthor=true&cauthor_uid=19491921), [Remy P](http://www.ncbi.nlm.nih.gov/pubmed?term=Remy%20P%5BAuthor%5D&cauthor=true&cauthor_uid=19491921): **Decrease of nicotinic receptors in the nigrostriatal system in Parkinson's disease.** *Journal of Cerebral Blood Flow & Metabolism* 2009, **29(9):**1601-1608.

151. Rinne JO, Laihinen A, Ruottinen H, Ruotsalainen U, Nagren K, Lehikoinen P, [Oikonen V](http://www.ncbi.nlm.nih.gov/pubmed?term=Oikonen%20V%5BAuthor%5D&cauthor=true&cauthor_uid=8543941), [Rinne UK](http://www.ncbi.nlm.nih.gov/pubmed?term=Rinne%20UK%5BAuthor%5D&cauthor=true&cauthor_uid=8543941): **Increased density of dopamine D2 receptors in the putamen, but not in the caudate nucleus in early Parkinson's disease: a PET study with [11C]raclopride.** *J Neurol Sci* 1995, **132(2):**156-161.

152. Takikawa S, Dhawan V, Chaly T, Robeson W, Dahl R, Zanzi I, [Mandel F](http://www.ncbi.nlm.nih.gov/pubmed?term=Mandel%20F%5BAuthor%5D&cauthor=true&cauthor_uid=8195882), [Spetsieris P](http://www.ncbi.nlm.nih.gov/pubmed?term=Spetsieris%20P%5BAuthor%5D&cauthor=true&cauthor_uid=8195882), [Eidelberg D](http://www.ncbi.nlm.nih.gov/pubmed?term=Eidelberg%20D%5BAuthor%5D&cauthor=true&cauthor_uid=8195882): **Input functions for 6-[fluorine-18]fluorodopa quantitation in parkinsonism: comparative studies and clinical correlations.** *J Nucl Med* 1994, **35(6):**955-963.

153. Politis M, Piccini P, Pavese N, Koh SB, Brooks DJ: **Evidence of dopamine dysfunction in the hypothalamus of patients with Parkinson's disease: an in vivo 11C-raclopride PET study.** *Experimental Neurology* 2008, **214(1):**112-116.

154. Lozza C, Baron JC, Eidelberg D, Mentis MJ, Carbon M, Marie RM: **Executive processes in Parkinson's disease: FDG-PET and network analysis.** *Hum Brain Mapp* 2004, **22(3):**236-245.

155. Doder M, Rabiner EA, Turjanski N, Lees AJ, Brooks DJ: **Tremor in Parkinson's disease and serotonergic dysfunction: an 11C-WAY 100635 PET study.** *Neurology* 2003, **60(4):**601-605.

156. Tanaka H, Koenig T, Pascual-Marqui RD, Hirata K, Kochi K, Lehmann D: **Event-related potential and EEG measures in Parkinson's disease without and with dementia.** *Dement Geriatr Cogn Disord* 2000, **11(1):**39-45.

157. Bhatia M, Johri S, Behari M: **Increased cortical excitability with longer duration of Parkinson's disease as evaluated by transcranial magnetic stimulation.** *Neurol India* 2003, **51(1):**13-15.

158. Robichaud JA, Pfann KD, Leurgans S, Vaillancourt DE, Comella CL, Corcos DM: **Variability of EMG patterns: a potential neurophysiological marker of Parkinson's disease?** *Clin Neurophysiol* 2009, **120(2):**390-397.

159. Ertekin C, Tarlaci S, Aydogdu I, Kiylioglu N, Yuceyar N, Turman AB, [Secil Y](http://www.ncbi.nlm.nih.gov/pubmed?term=Secil%20Y%5BAuthor%5D&cauthor=true&cauthor_uid=12360543), [Esmeli F](http://www.ncbi.nlm.nih.gov/pubmed?term=Esmeli%20F%5BAuthor%5D&cauthor=true&cauthor_uid=12360543): **Electrophysiological evaluation of pharyngeal phase of swallowing in patients with Parkinson's disease.** *Mov Disord* 2002, **17(5):**942-949.

160. Papapetropoulos S, Argyriou AA, Chroni E: **No correlation between the clinical severity of autonomic symptoms (SCOPA-AUT) and electrophysiological test abnormalities in advanced Parkinson's disease.** *Mov Disord* 2006, **21(3):**430-431.

161. Haapaniemi TH, Pursiainen V, Korpelainen JT, Huikuri HV, Sotaniemi KA, Myllyla VV: **Ambulatory ECG and analysis of heart rate variability in Parkinson's disease.** *J Neurol Neurosurg Psychiatry* 2001, **70(3):**305-310.

162. Forte G, Alimonti A, Violante N, Di GM, Senofonte O, Petrucci F, [Sancesario G](http://www.ncbi.nlm.nih.gov/pubmed?term=Sancesario%20G%5BAuthor%5D&cauthor=true&cauthor_uid=16325536), [Bocca B](http://www.ncbi.nlm.nih.gov/pubmed?term=Bocca%20B%5BAuthor%5D&cauthor=true&cauthor_uid=16325536): **Calcium, copper, iron, magnesium, silicon and zinc content of hair in Parkinson's disease.** *J Trace Elem Med Biol* 2005, **19(2-3):**195-201.

163. Monte FS, da Silva-Junior FP, Braga-Neto P, Nobre e Souza MA, de Bruin VM: **Swallowing abnormalities and dyskinesia in Parkinson's disease.** *Mov Disord* 2005, **20(4):**457-462.

164. Blessed G, Tomlinson BE, Roth M: **The association between quantitative measures of dementia and of senile change in the cerebral grey matter of elderly subjects.** *Br J Psychiatry* 1968, **114(512):**797-811.

165. Hely MA, Chey T, Wilson A, Williamson PM, O'Sullivan DJ, Rail D, Morris JG: **Reliability of the Columbia scale for assessing signs of Parkinson's disease.** *Mov Disord* 1993, **8(4):**466-472.

166. Brown GG, Rahill AA, Gorell JM, McDonald C, Brown SJ, Sillanpaa M, Shults C: **Validity of the Dementia Rating Scale in assessing cognitive function in Parkinson's disease.** *J Geriatr Psychiatry Neurol* 1999, **12(4):**180-188.

167. Reisberg B, Ferris SH, de Leon MJ, Crook T: **The Global Deterioration Scale for assessment of primary degenerative dementia.** *Am J Psychiatry* 1982, **139(9):**1136-1139.

168. Goetz CG, Poewe W, Rascol O, Sampaio C, Stebbins GT, Counsell C, [Giladi N](http://www.ncbi.nlm.nih.gov/pubmed?term=Giladi%20N%5BAuthor%5D&cauthor=true&cauthor_uid=15372591), [Holloway RG](http://www.ncbi.nlm.nih.gov/pubmed?term=Holloway%20RG%5BAuthor%5D&cauthor=true&cauthor_uid=15372591), [Moore CG](http://www.ncbi.nlm.nih.gov/pubmed?term=Moore%20CG%5BAuthor%5D&cauthor=true&cauthor_uid=15372591), [Wenning GK](http://www.ncbi.nlm.nih.gov/pubmed?term=Wenning%20GK%5BAuthor%5D&cauthor=true&cauthor_uid=15372591), [Yahr MD](http://www.ncbi.nlm.nih.gov/pubmed?term=Yahr%20MD%5BAuthor%5D&cauthor=true&cauthor_uid=15372591), [Seidl L](http://www.ncbi.nlm.nih.gov/pubmed?term=Seidl%20L%5BAuthor%5D&cauthor=true&cauthor_uid=15372591); [Movement Disorder Society Task Force on Rating Scales for Parkinson's Disease](http://www.ncbi.nlm.nih.gov/pubmed?term=Movement%20Disorder%20Society%20Task%20Force%20on%20Rating%20Scales%20for%20Parkinson's%20Disease%5BCorporate%20Author%5D): **Movement Disorder Society Task Force report on the Hoehn and Yahr staging scale: status and recommendations.** *Mov Disord* 2004, **19(9):**1020-1028.

169. Yukimichi I, Hasegawa K: **The revised Hasegawa's Dementia Scale (HDS-R) - Evaluation of its usefulness as a screening test for dementia.** *The Hong Kong Journal of Psychiatry* 1994, **4:**20-24.

170. Folstein MF, Folstein SE, McHugh PR: **"Mini-mental state". A practical method for grading the cognitive state of patients for the clinician.** *J Psychiatr Res* 1975, **12(3):**189-198.

171. Canter GJ, De La Torre R, Mier M: **A method for evaluating disability in patients with Parkinson's disease.** *J Nerv Ment Dis* 1961, **133:**143-147.

172. Schwab R, England A: **Projection technique for evaluating surgery in Parkinson's disease.** In *Third Symposium on Parkinson's Disease*. Edited by Gillingham F, Donaldson I. Edinburgh: *E&S* Livingstone: 1969.

173. Visser M, Marinus J, Stiggelbout AM, Van Hilten JJ: **Assessment of autonomic dysfunction in Parkinson's disease: the SCOPA-AUT.** *Mov Disord* 2004, **19(11):**1306-1312.

174. Roth M, Tym E, Mountjoy CQ, Huppert FA, Hendrie H, Verma S, Goddard R: **CAMDEX. A standardised instrument for the diagnosis of mental disorder in the elderly with special reference to the early detection of dementia.** *Br J Psychiatry* 1986, **149:**698-709.

175. Fahn S, Eton RL, UPDRS Development Committee: **The Unified Parkinson's Disease Rating Scale.** In *Recent Developments in Parkinson's Disease. Volume 2.* Edited by Fahn S, Marsden CD, Goldstein M, Calne DB. Florham Park, New Jersey: Macmillan Healthcare Information; 1987:153-163.

176. Webster DD: **Critical analysis of the disability in Parkinson's disease.** *Mod Treat* 1968, **5(2):**257-282.
